# Supplementary figures and images for: The deubiquitinase Ubp3/Usp10 constrains glucose-mediated mitochondrial repression via phosphate budgeting
Source: eLife. 2024 Sep 26;12:RP90293. doi: 10.7554/eLife.90293 (PMC11426969; doi:10.7554/eLife.90293)

Figure 1E-Cox2 levels in WT and *ubp3Δ* cells

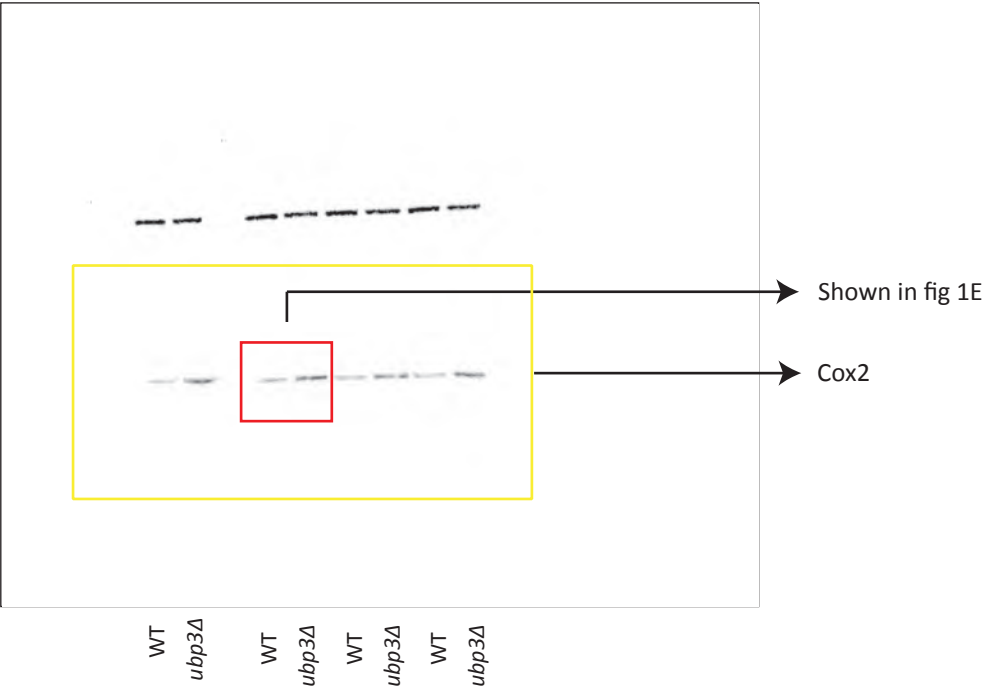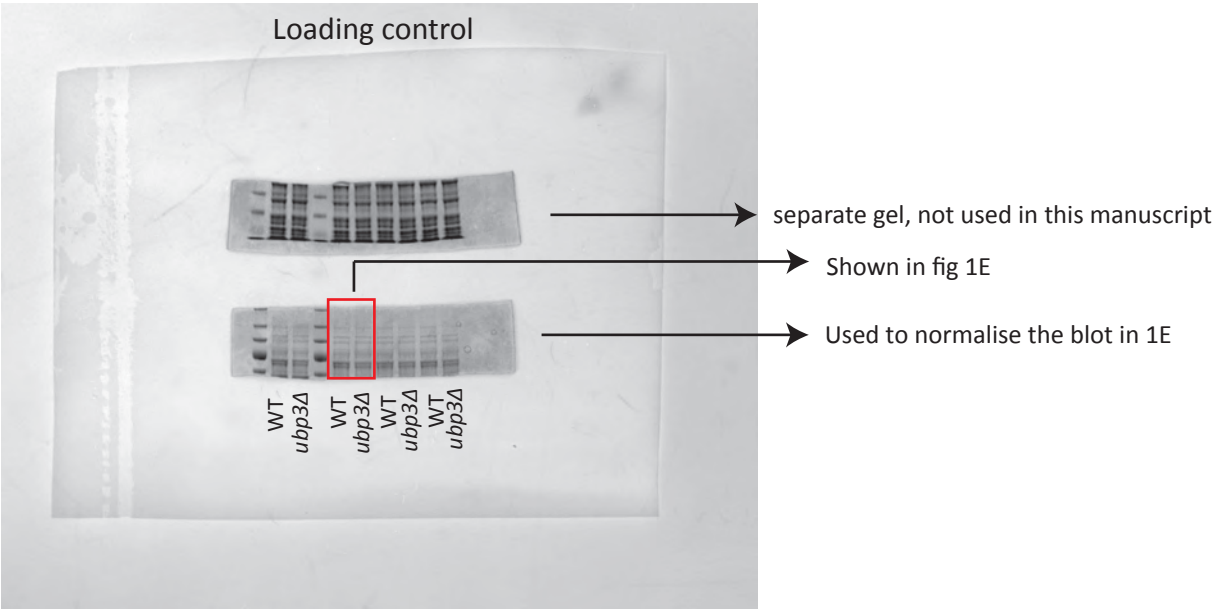

Supplement: Figure 1—source data 1. [file elife-90293-fig1-data1.zip › Figure 1/Figure 1-source data, uncropped and labelled gels.pdf]

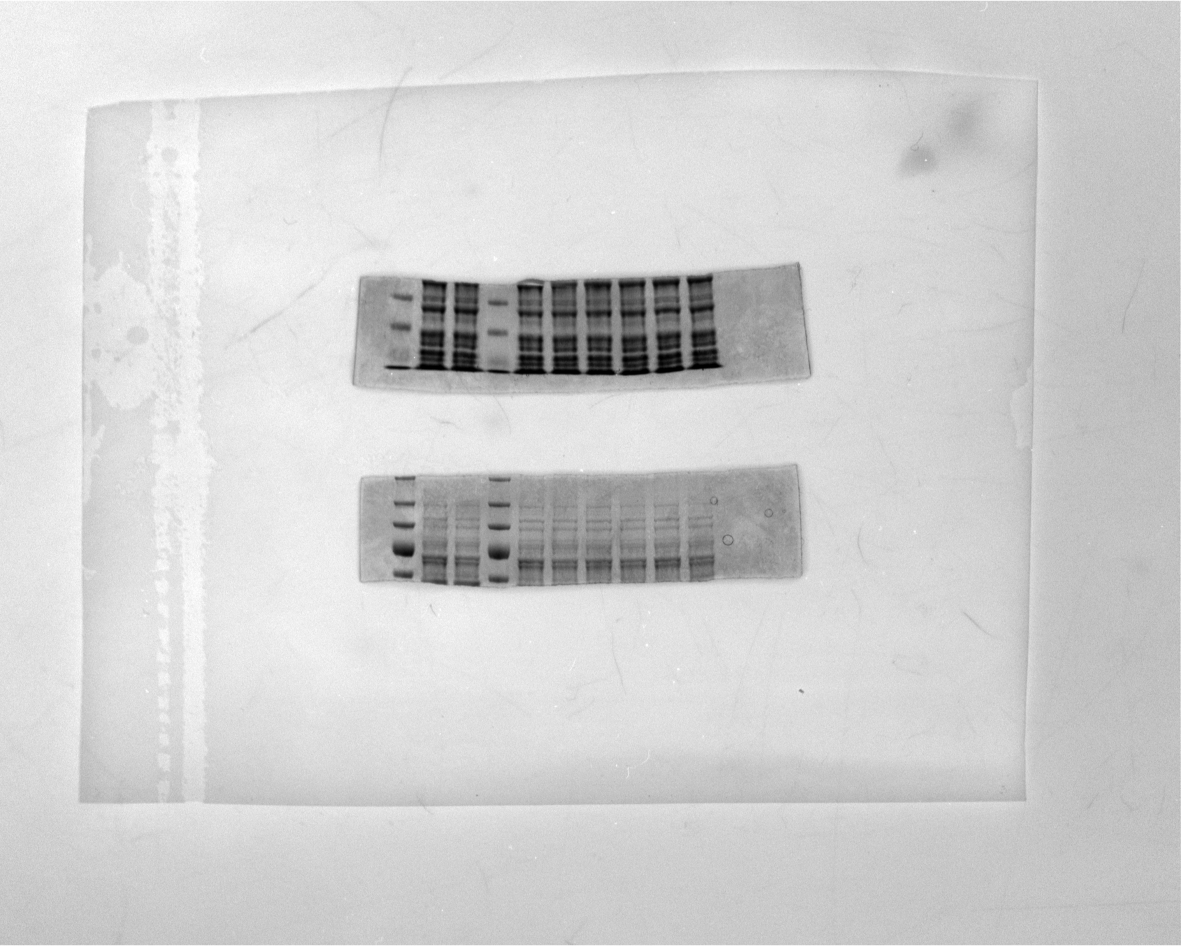

Supplement: Figure 1—source data 2. [file elife-90293-fig1-data2.zip › Figure 1 raw unedited/Figure 1E loading control.tif]

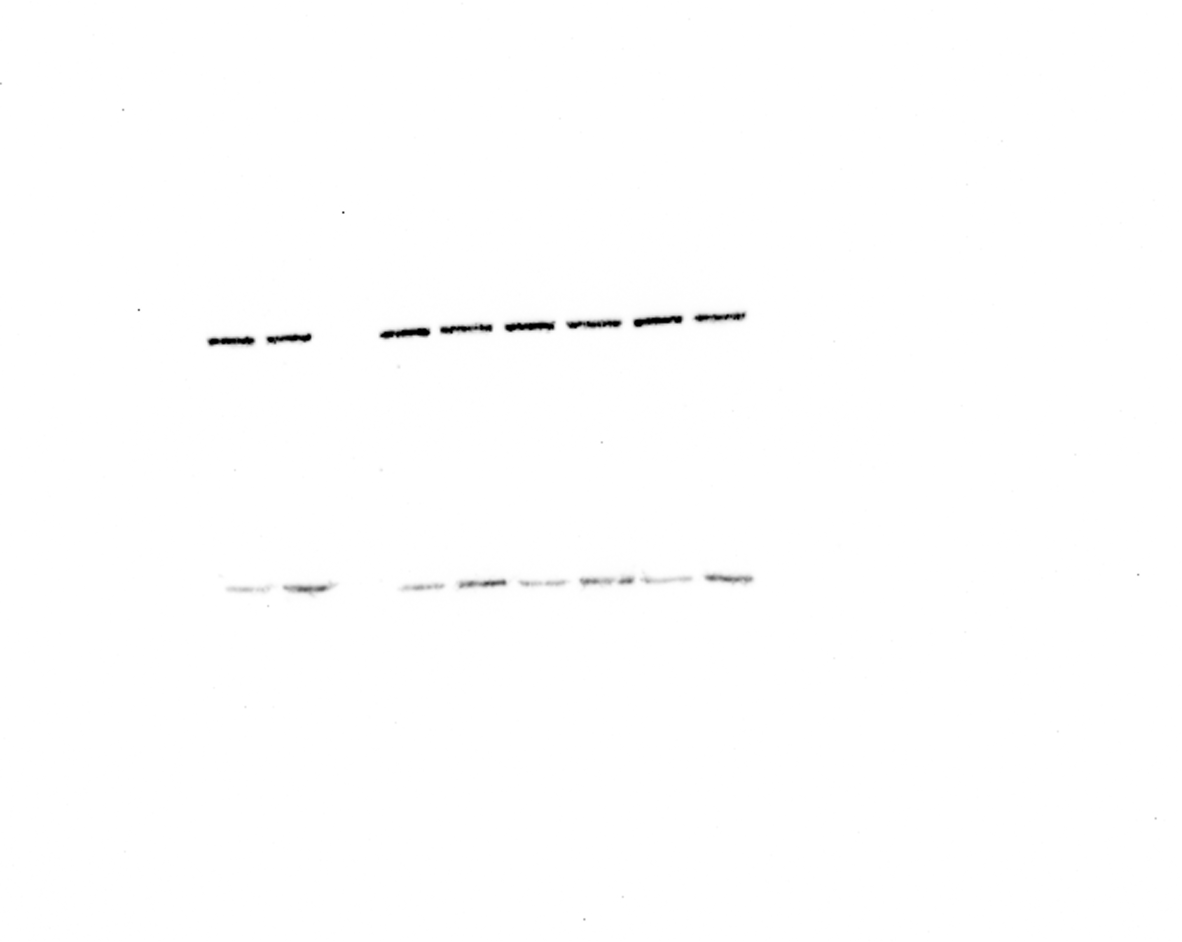

Supplement: Figure 1—source data 2. [file elife-90293-fig1-data2.zip › Figure 1 raw unedited/Figure 1E.tif]

Figure 1-figure supplement 1J - Total ubiquitination in ubp3 and WT

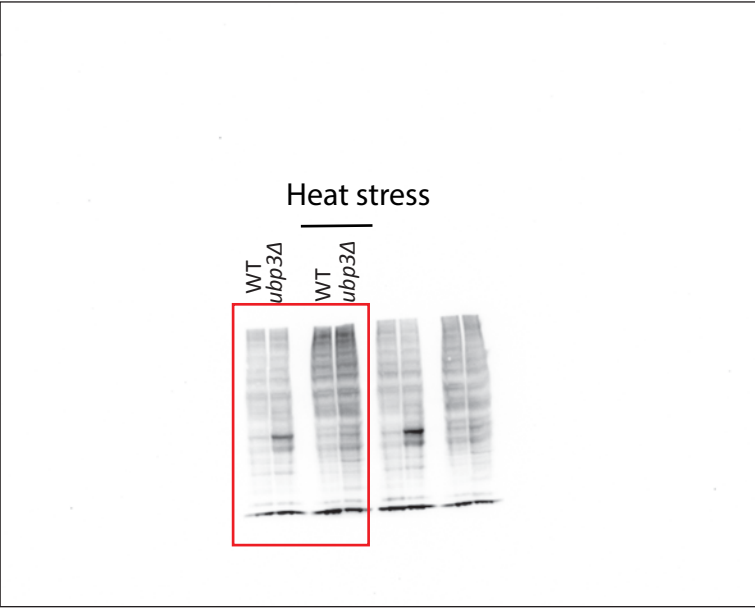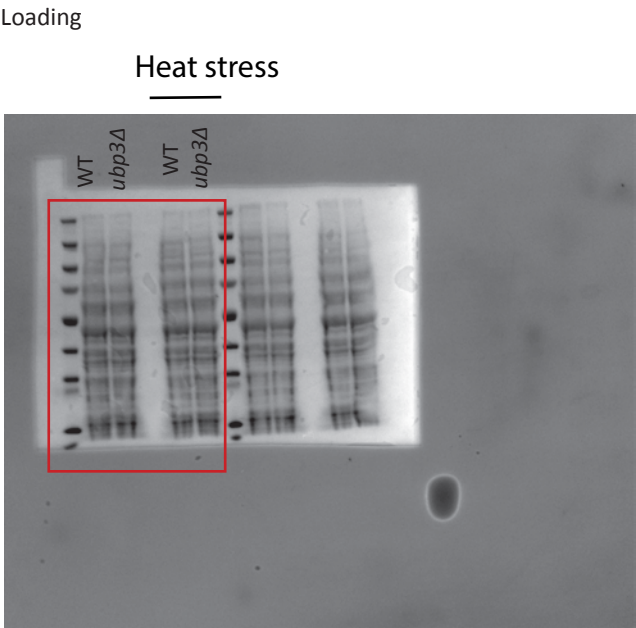

Supplement: Figure 1—figure supplement 1—source data 1. [file elife-90293-fig1-figsupp1-data1.zip › Figure 1, figure supplement 1/Figure 1, figure supplement 1-source data 3, uncropped and labelled gels.pdf]

Figure 1-figure supplement 1E-Ubp3 levels in WT and Ubp3<sup>C469A</sup> cells

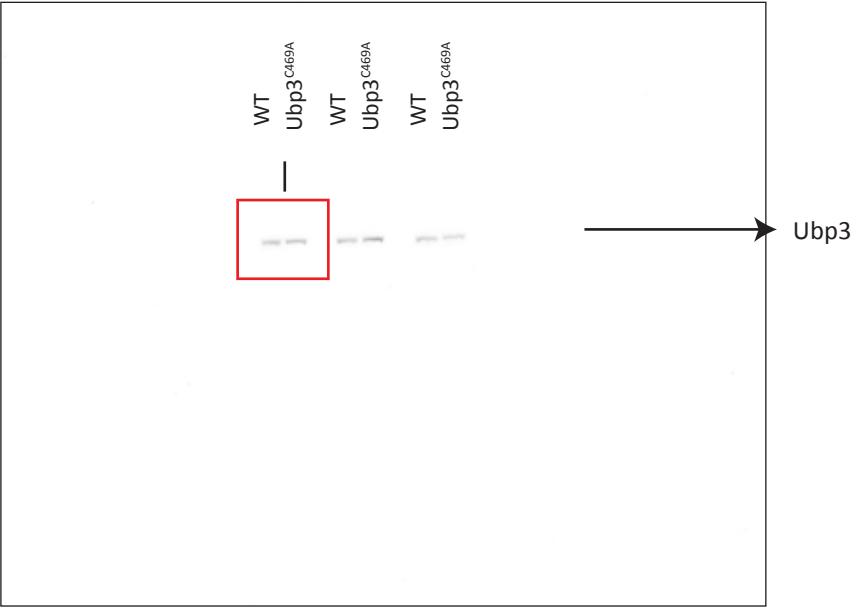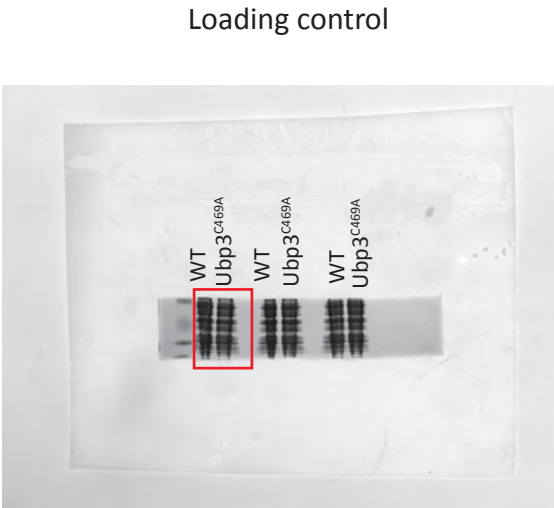

Supplement: Figure 1—figure supplement 1—source data 1. [file elife-90293-fig1-figsupp1-data1.zip › Figure 1, figure supplement 1/Figure 1, figure supplement 1-source data 1, uncropped and labelled gels.pdf]

**Figure 1-figure supplement 1G**-Tom70 levels in WT and *ububp3Δ* *cellsp3Δ* cells

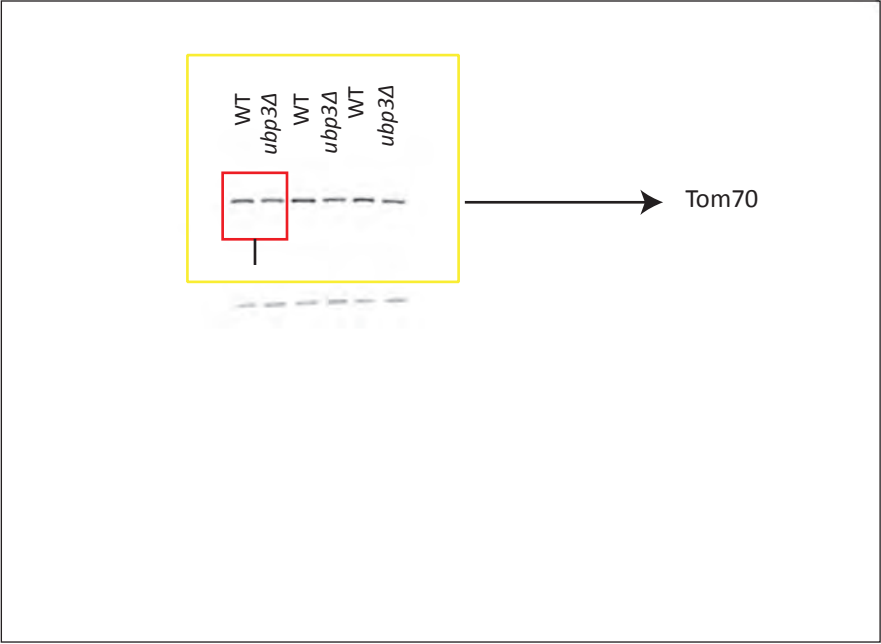

Loading control

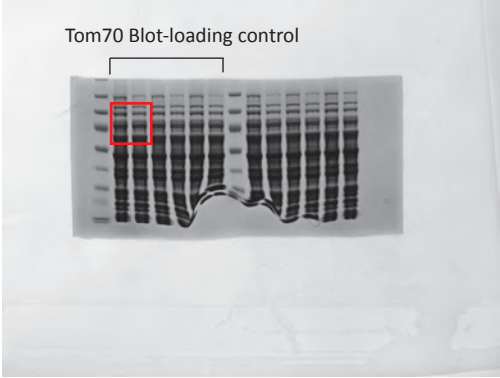

Supplement: Figure 1—figure supplement 1—source data 1. [file elife-90293-fig1-figsupp1-data1.zip › Figure 1, figure supplement 1/Figure 1, figure supplement 1-source data 2, uncropped and labelled gels.pdf]

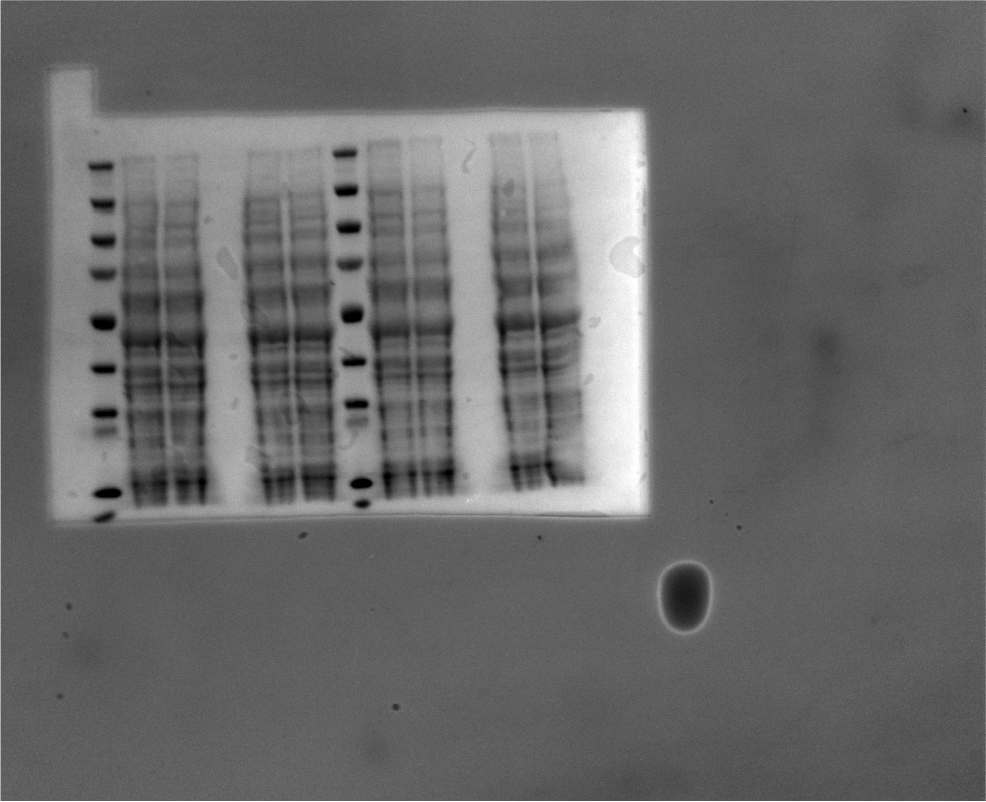

Supplement: Figure 1—figure supplement 1—source data 2. [file elife-90293-fig2-figsupp2-data2.zip › Figure 1, figure supplement 1 raw unedited/Figure 1, supplement 1J loading.tif]

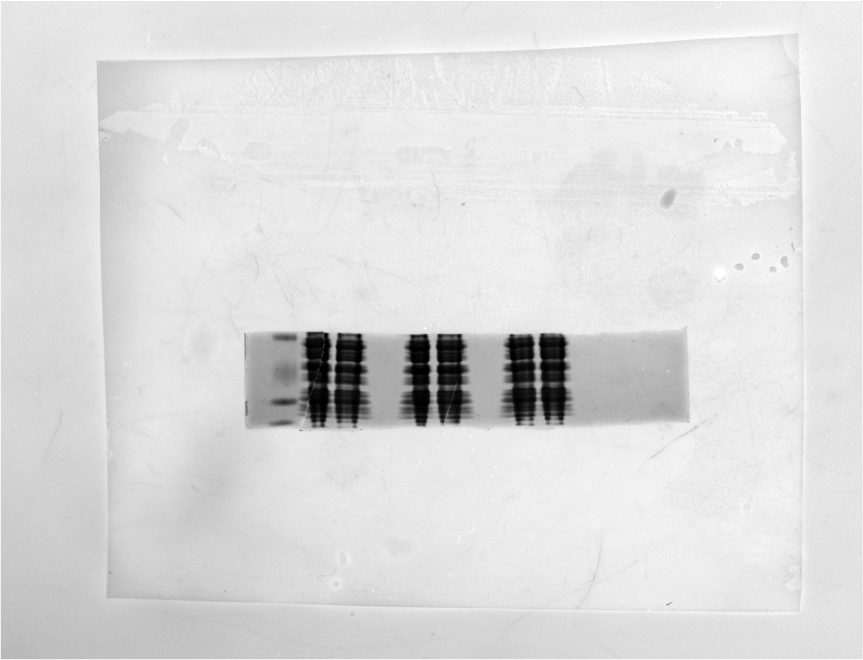

Supplement: Figure 1—figure supplement 1—source data 2. [file elife-90293-fig2-figsupp2-data2.zip › Figure 1, figure supplement 1 raw unedited/Figure 1, supplement 1E loading.tif]

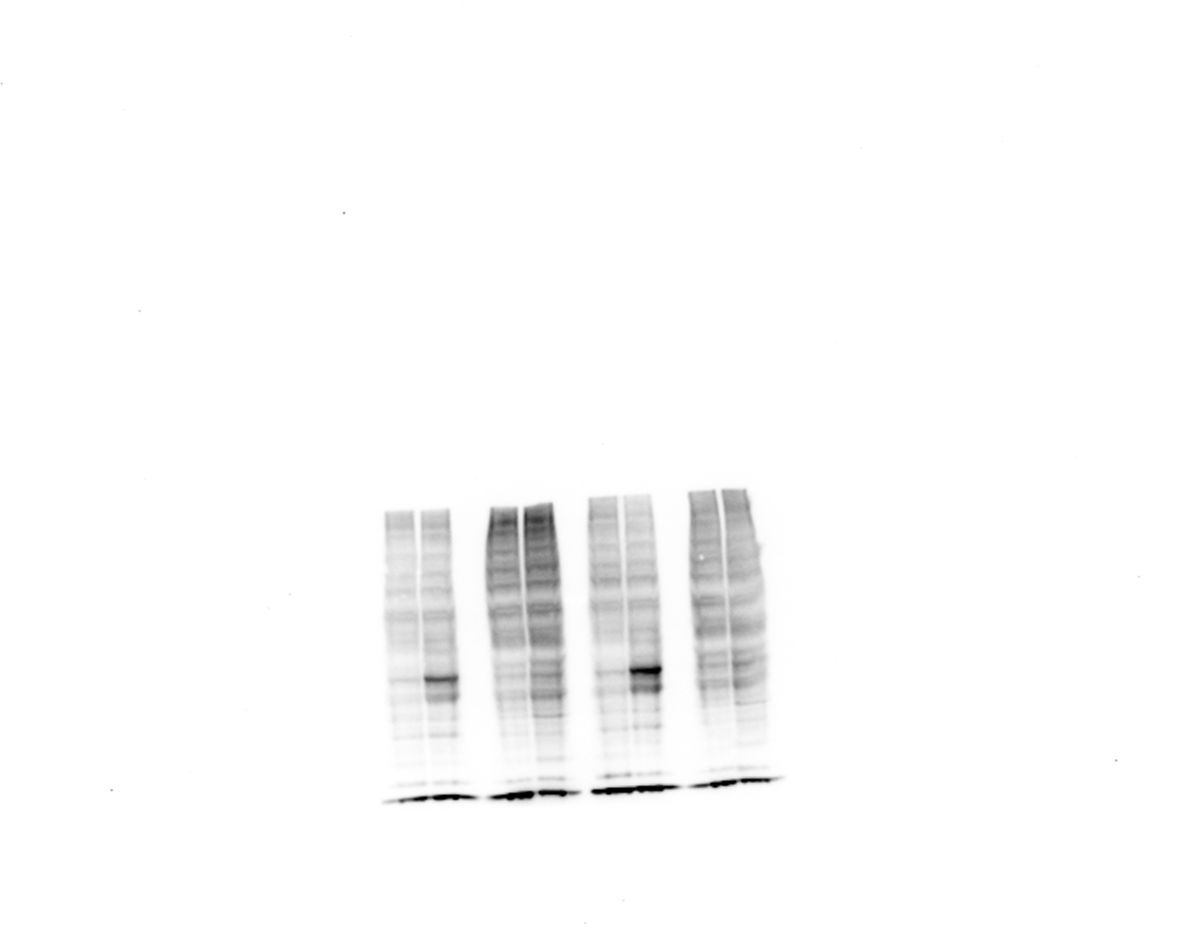

Supplement: Figure 1—figure supplement 1—source data 2. [file elife-90293-fig2-figsupp2-data2.zip › Figure 1, figure supplement 1 raw unedited/Figure 1, supplement 1J.tif]

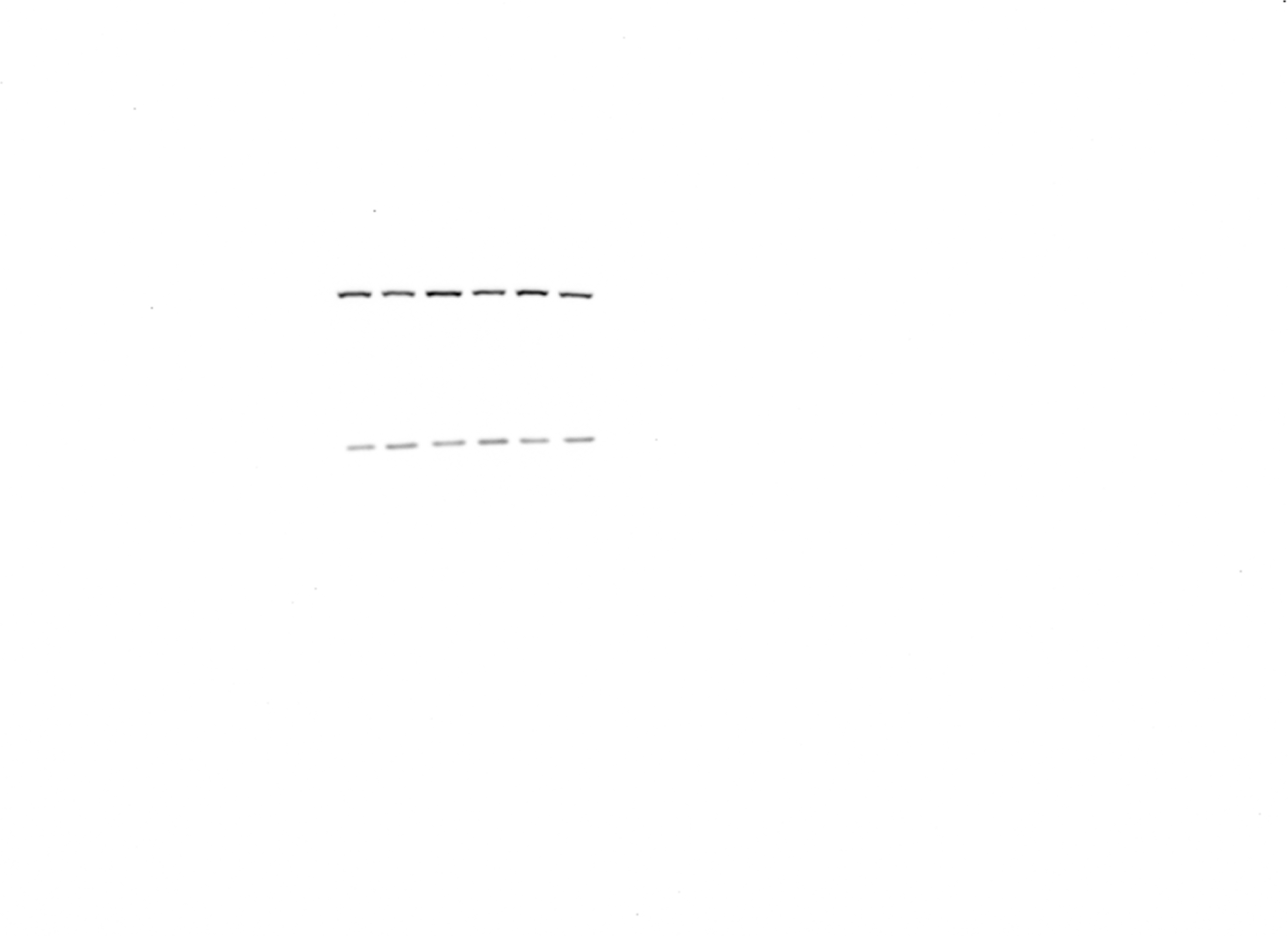

Supplement: Figure 1—figure supplement 1—source data 2. [file elife-90293-fig2-figsupp2-data2.zip › Figure 1, figure supplement 1 raw unedited/Figure 1, supplement 1G.tif]

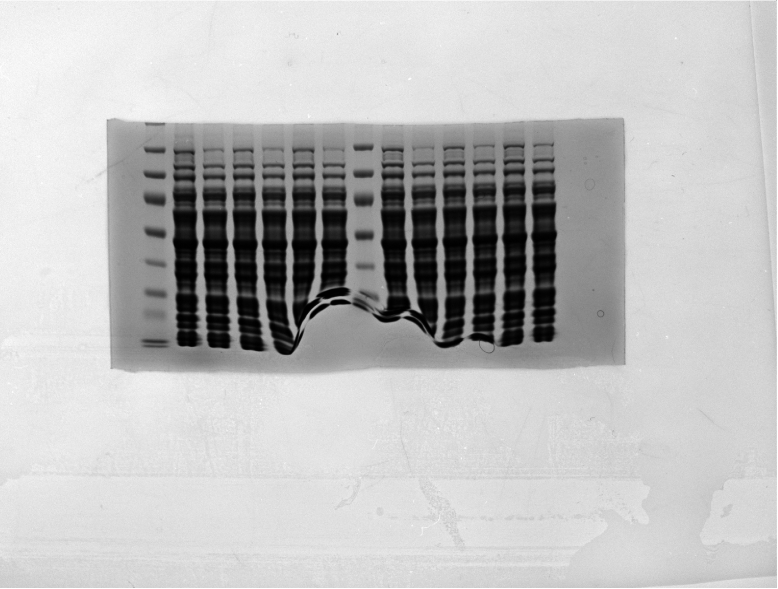

Supplement: Figure 1—figure supplement 1—source data 2. [file elife-90293-fig2-figsupp2-data2.zip › Figure 1, figure supplement 1 raw unedited/Figure 1, supplement 1G, loading.tif]

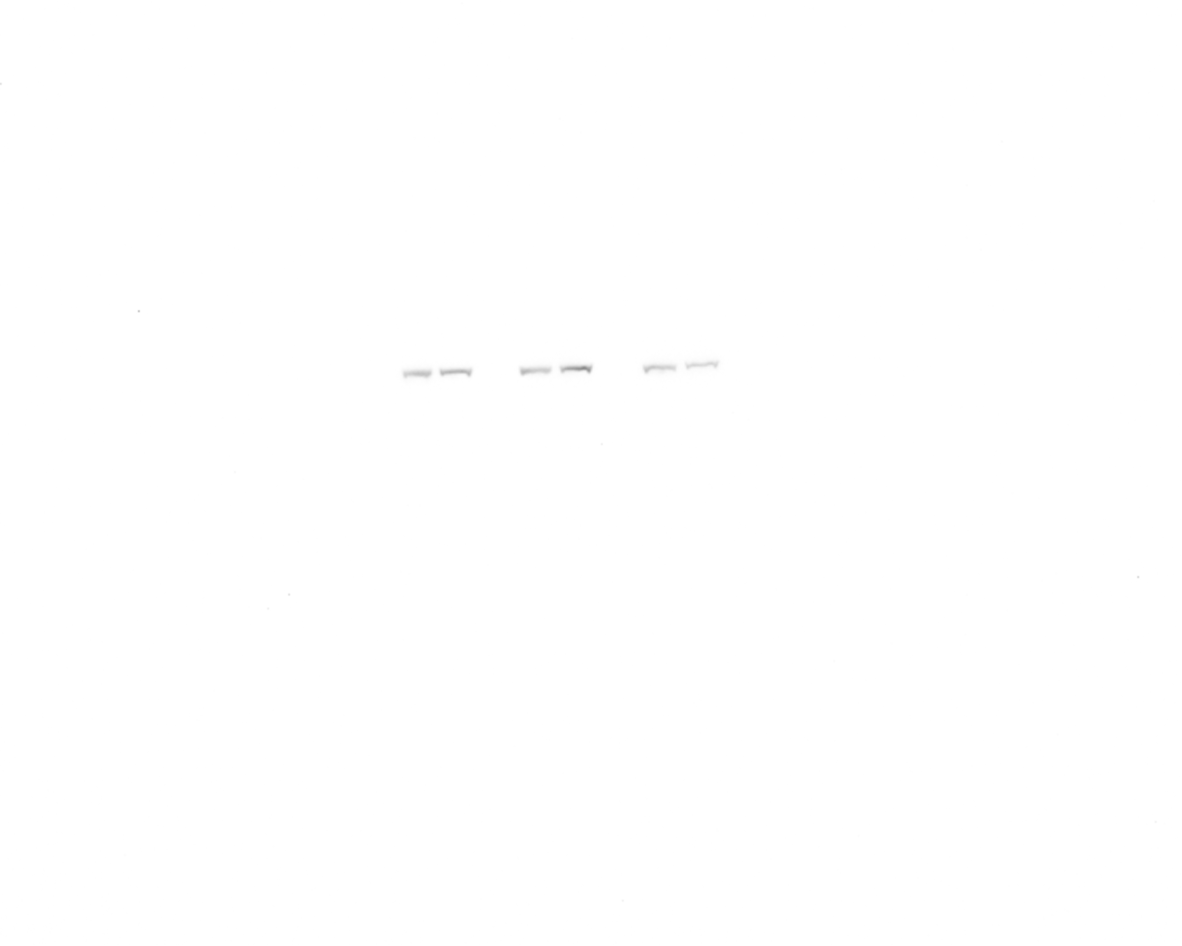

Supplement: Figure 1—figure supplement 1—source data 2. [file elife-90293-fig2-figsupp2-data2.zip › Figure 1, figure supplement 1 raw unedited/Figure 1, supplement 1E.tif]

Figure 2B-Pfk1,Tdh2,Tdh3 in WT and *ubp3Δ*

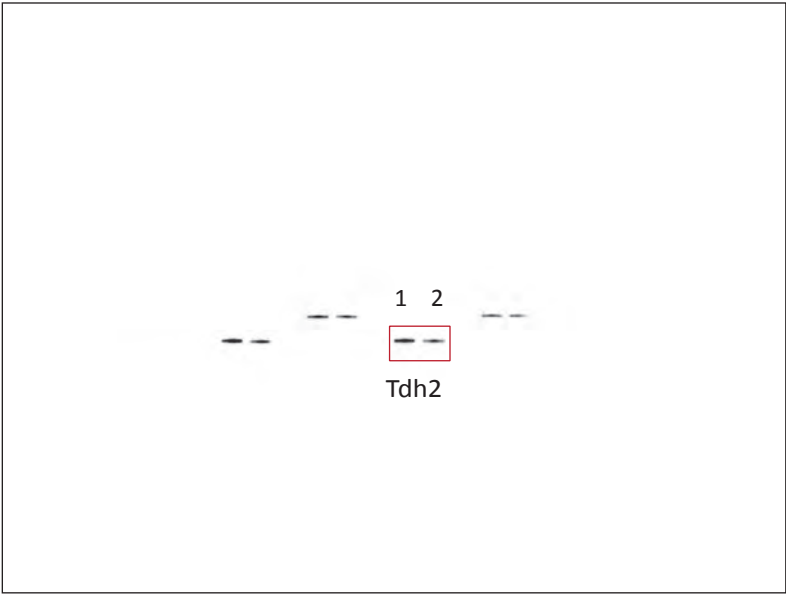

Loading

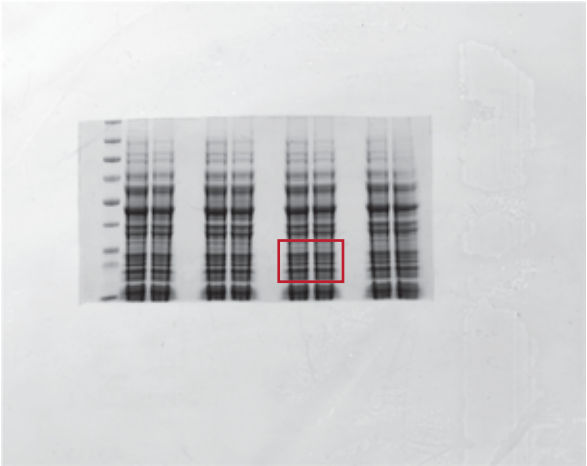

Supplement: Figure 2—source data 1. [file elife-90293-fig2-data1.zip › Figure 2/Figure 2-source data 3, uncropped and labelled gels.pdf]

Figure 2B-Pfk1,Tdh2,Tdh3 in WT and *ubp3Δ*

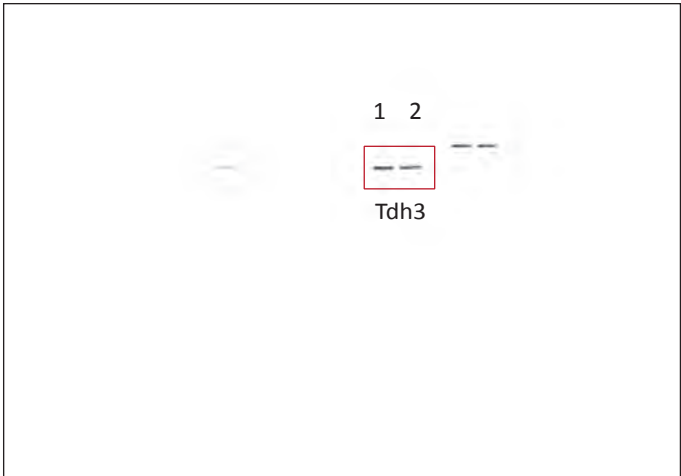

1- WT, 2- *ubp3Δ*

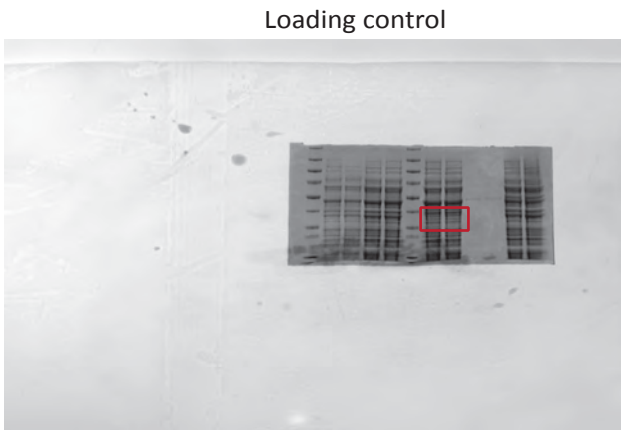

Supplement: Figure 2—source data 1. [file elife-90293-fig2-data1.zip › Figure 2/Figure 2-source data 2, uncropped and labelled gels.pdf]

Figure 2B-Pfk1,Tdh2,Tdh3 in WT and *ubp3Δ*

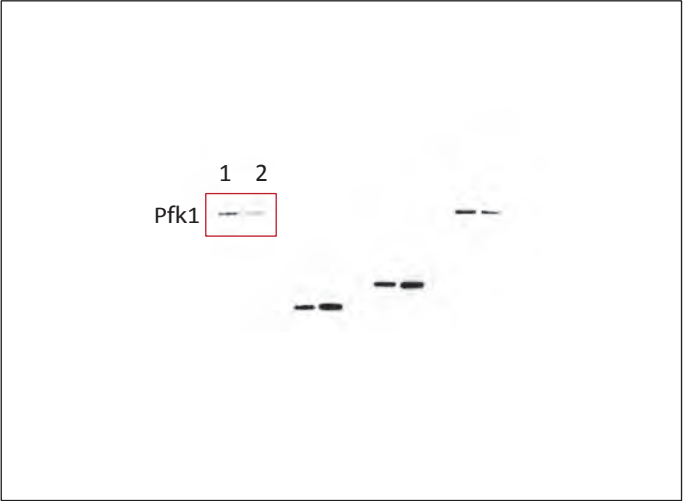

Loading control

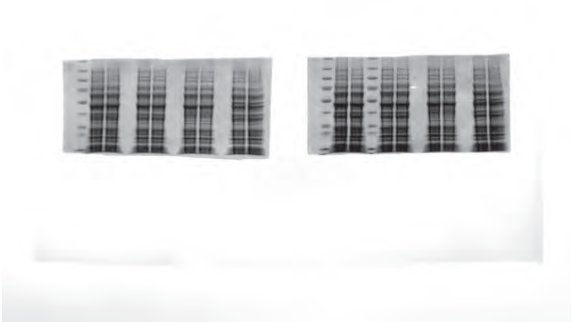

Supplement: Figure 2—source data 1. [file elife-90293-fig2-data1.zip › Figure 2/Figure 2-source data 1, uncropped and labelled gels.pdf]

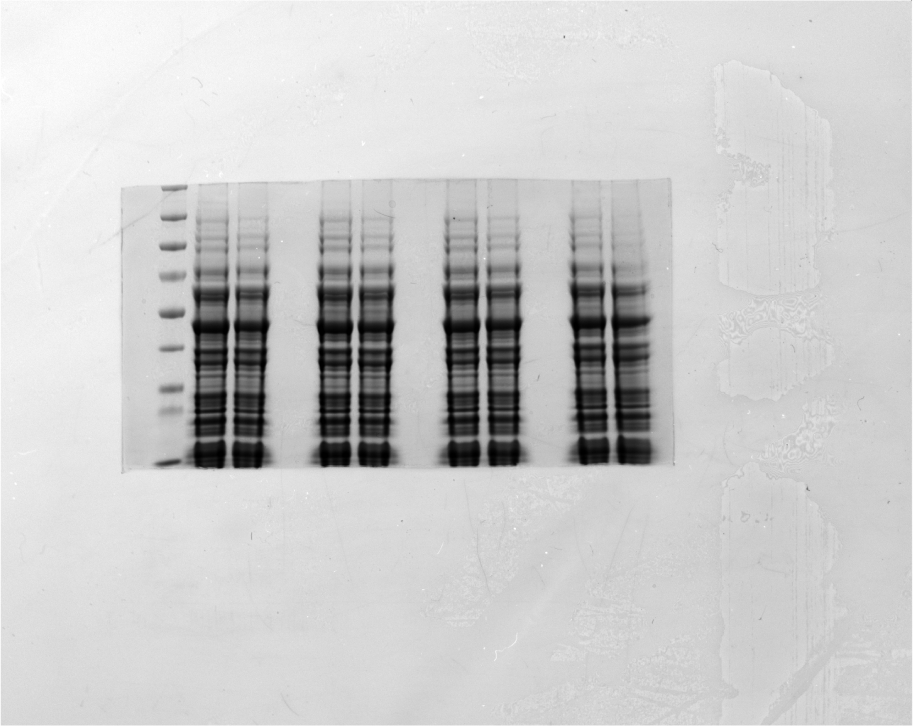

Supplement: Figure 2—source data 2. [file elife-90293-fig2-data2.zip › Figure 2 raw unedited/Figure 2B Loading_3.tif]

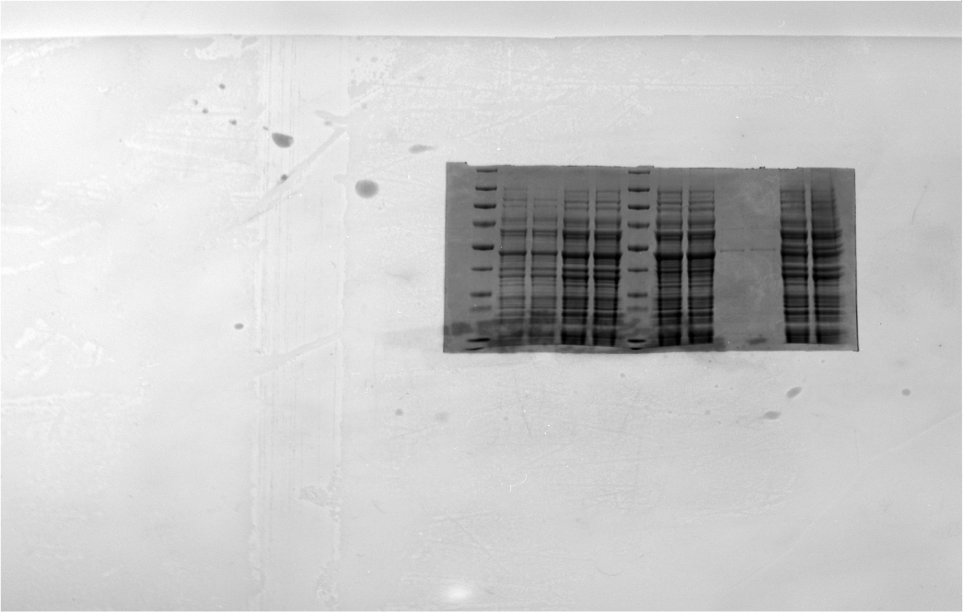

Supplement: Figure 2—source data 2. [file elife-90293-fig2-data2.zip › Figure 2 raw unedited/Figure 2B Loading_2.tif]

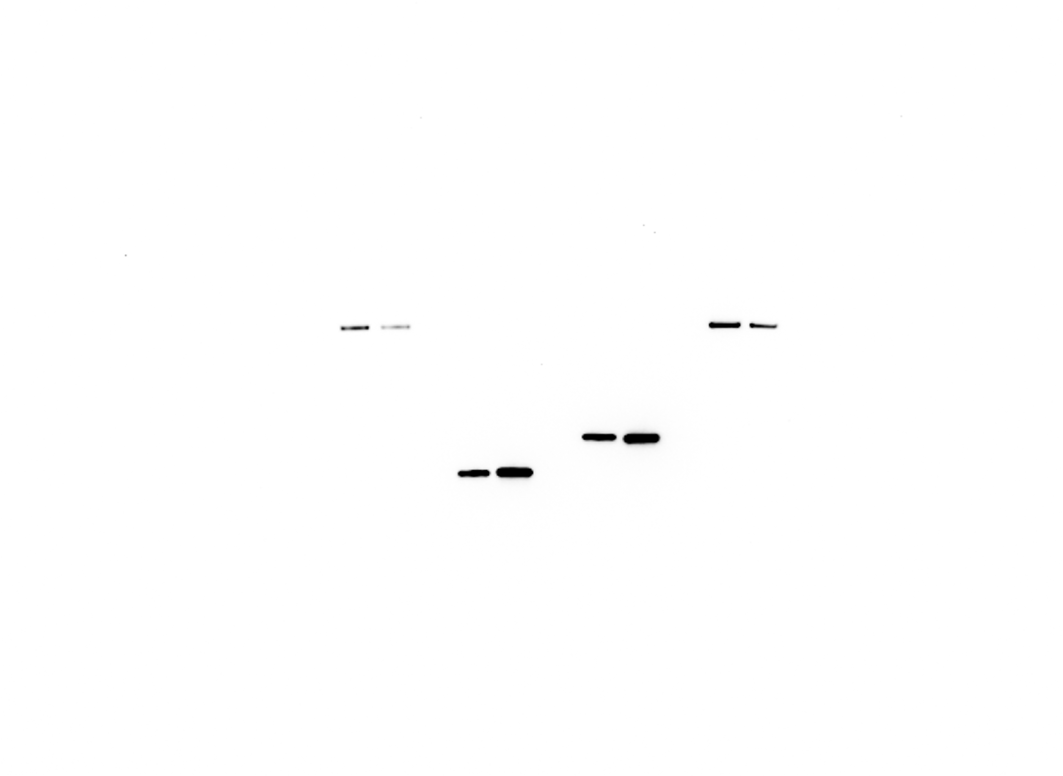

Supplement: Figure 2—source data 2. [file elife-90293-fig2-data2.zip › Figure 2 raw unedited/Figure 2B.tif]

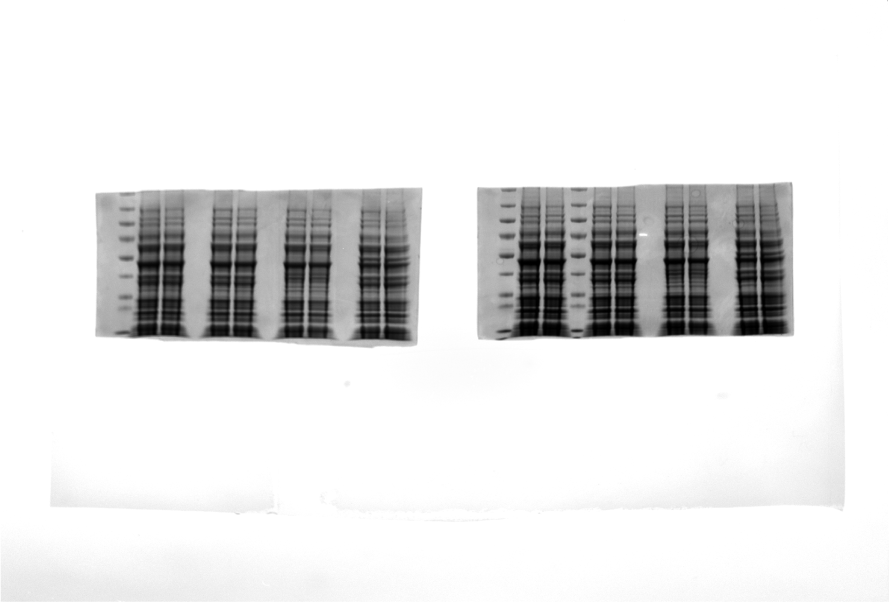

Supplement: Figure 2—source data 2. [file elife-90293-fig2-data2.zip › Figure 2 raw unedited/Figure 2B, loading.tif]

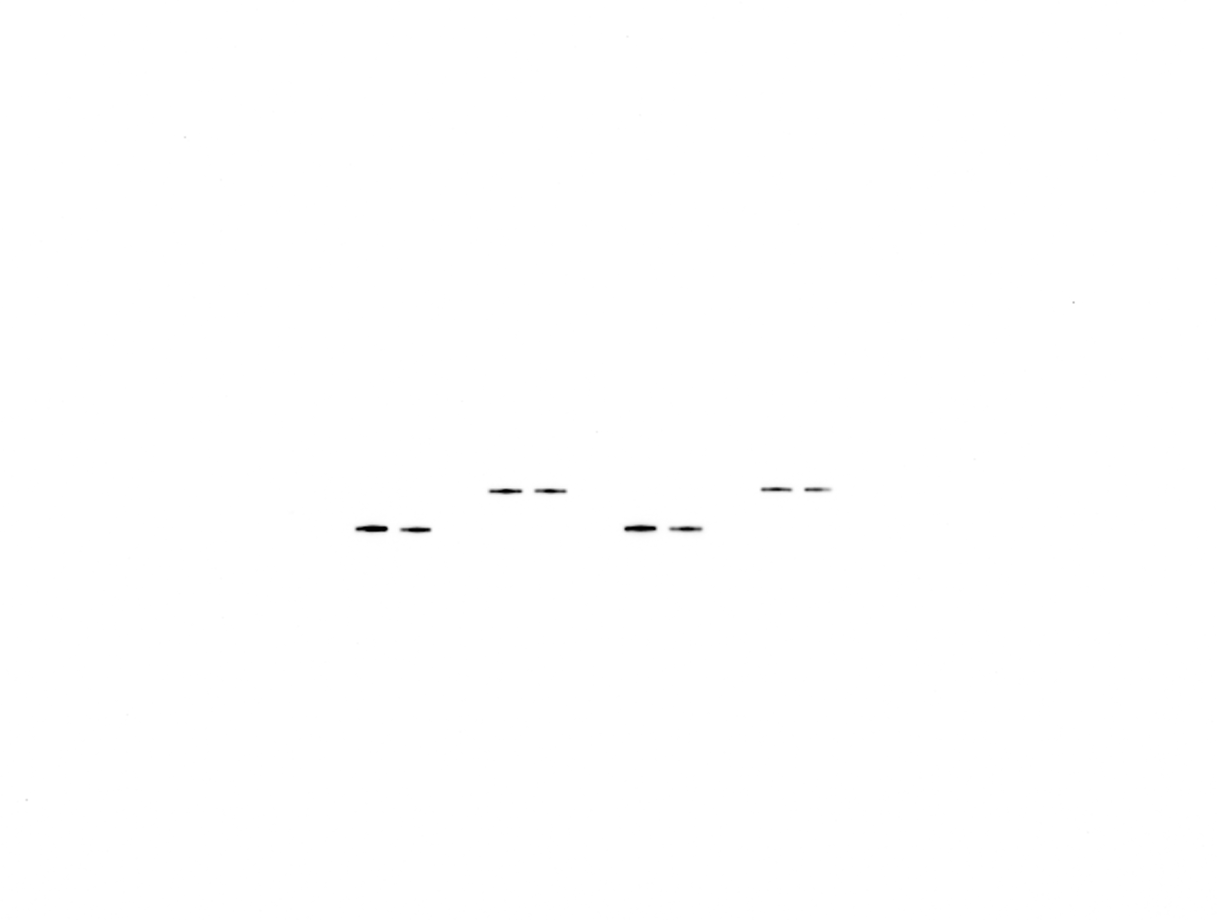

Supplement: Figure 2—source data 2. [file elife-90293-fig2-data2.zip › Figure 2 raw unedited/Figure 2B_3.tif]

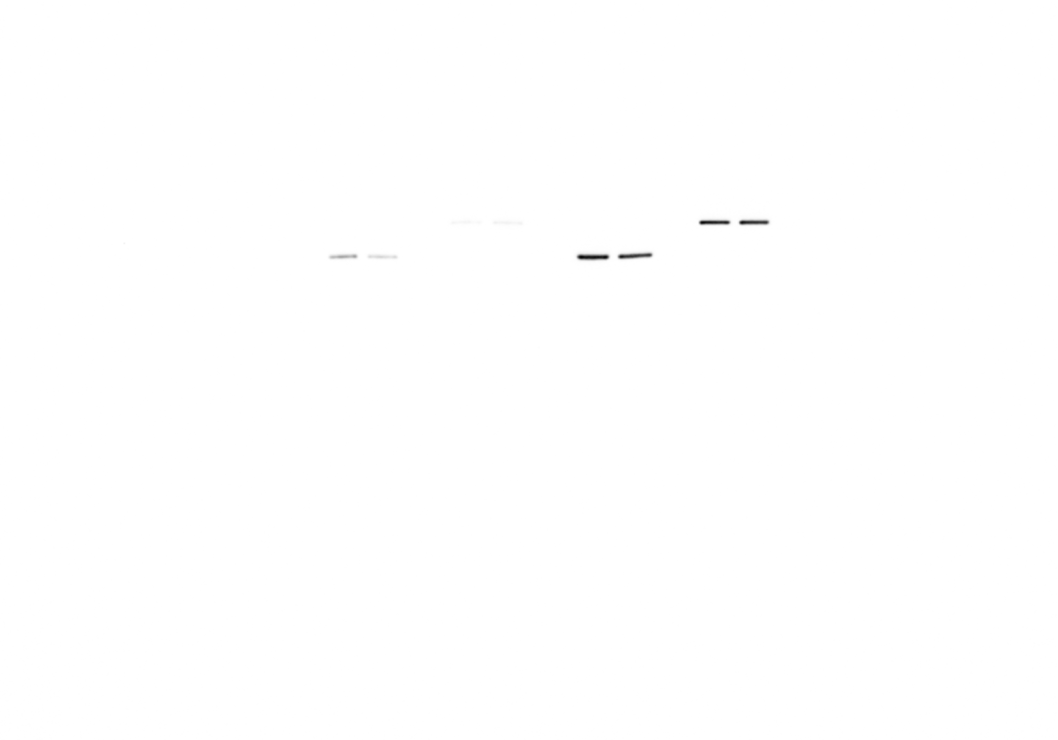

Supplement: Figure 2—source data 2. [file elife-90293-fig2-data2.zip › Figure 2 raw unedited/Figure 2B_2.tif]

Figure 2-figure supplement 1A-Eno1 and Eno2 in WT and *ubp3Δ*

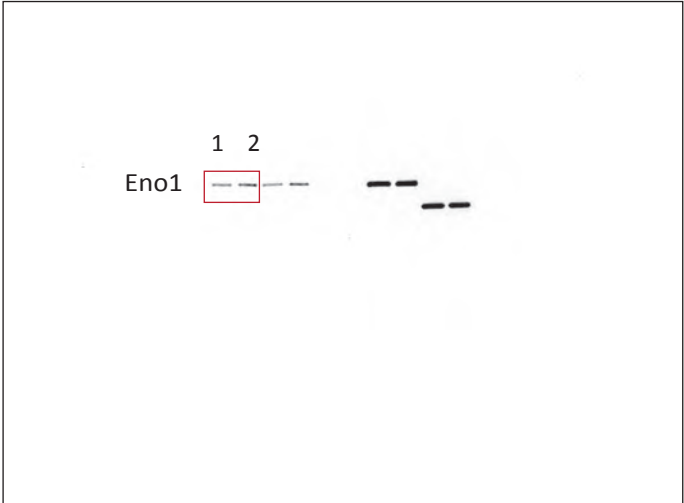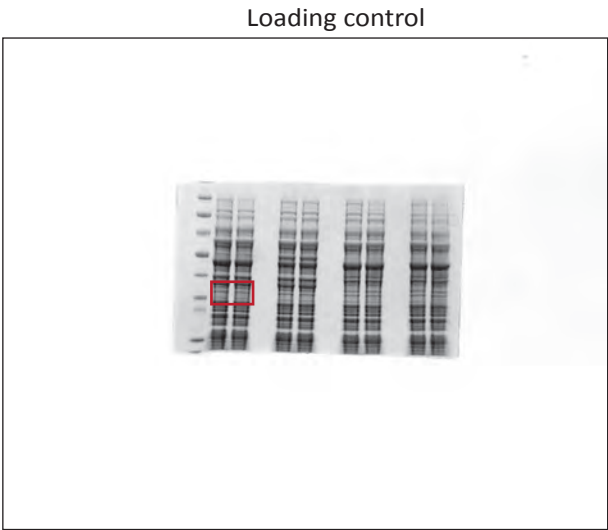

1- WT, 2- *ubp3Δ*

Supplement: Figure 2—figure supplement 1—source data 1. [file elife-90293-fig3-figsupp3-data3.zip › Figure 2, figure supplement 1/Figure 2, figure supplement 1-source data 1, uncropped and labelled gels.pdf]

Figure 2-figure supplement 1A-Eno1 and Eno2 in WT and *ubp3Δ*

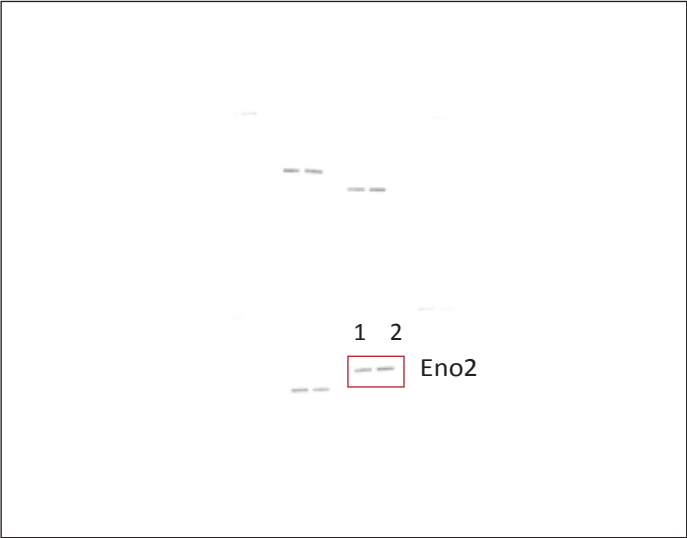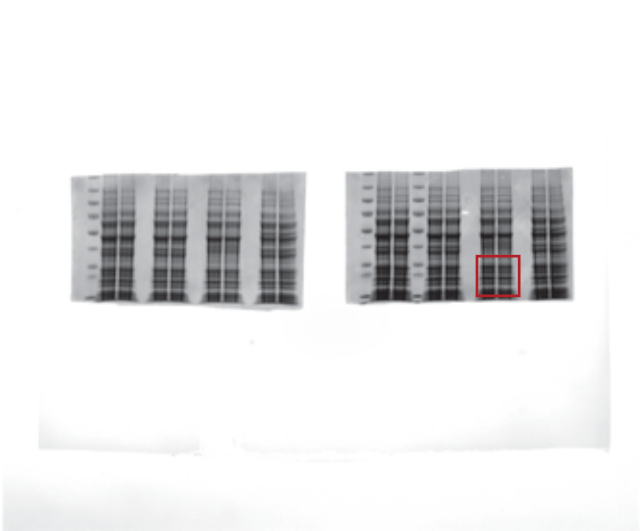

1- WT, 2- *ubp3Δ*

Supplement: Figure 2—figure supplement 1—source data 1. [file elife-90293-fig3-figsupp3-data3.zip › Figure 2, figure supplement 1/Figure 2, figure supplement 1-source data 2, uncropped and labelled gels.pdf]

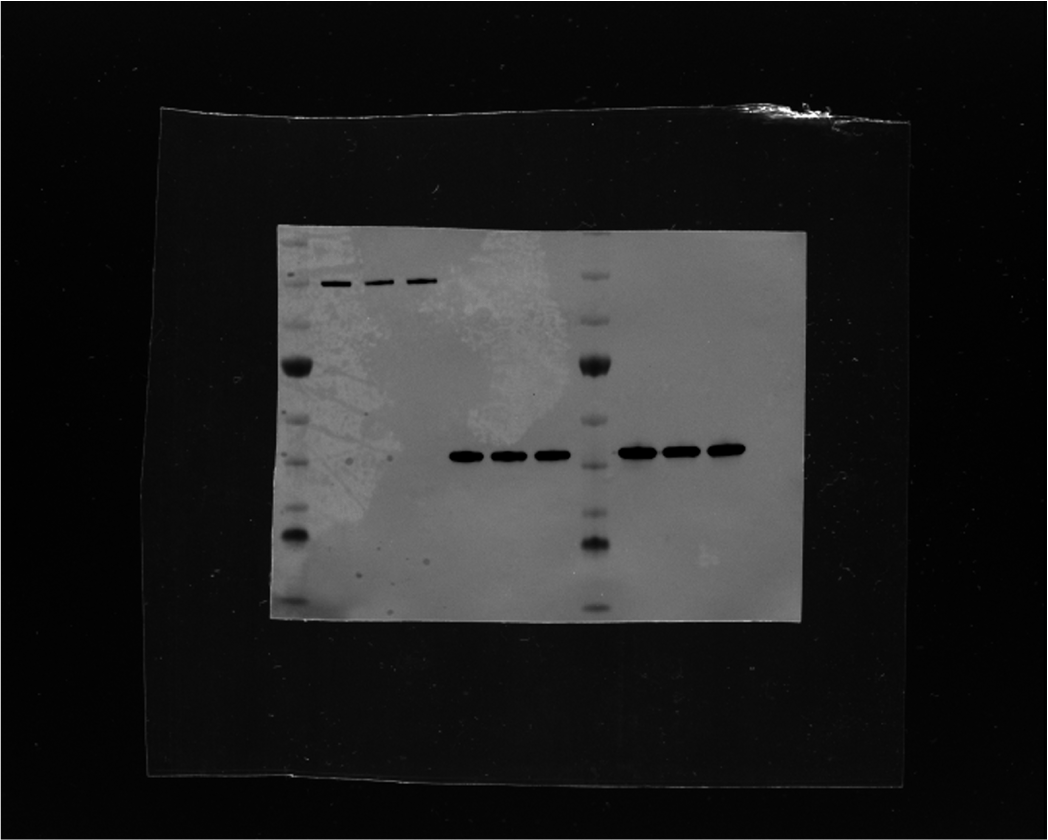

Supplement: Figure 2—figure supplement 1—source data 2. [file elife-90293-fig4-figsupp4-data4.zip › Figure 2, figure supplement 1 raw unedited/Figure 2, supplement 1B_2.tif]

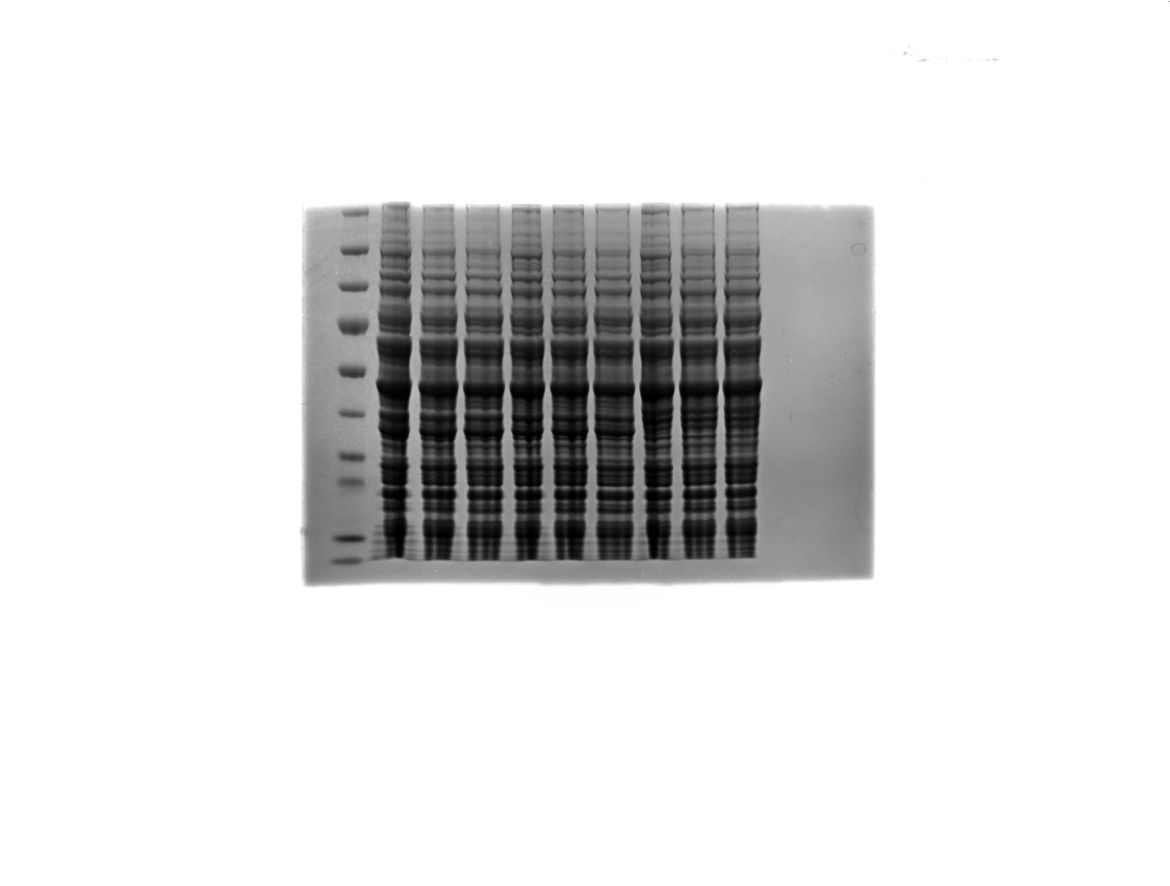

Supplement: Figure 2—figure supplement 1—source data 2. [file elife-90293-fig4-figsupp4-data4.zip › Figure 2, figure supplement 1 raw unedited/Figure 2, supplement 1B loading.tif]

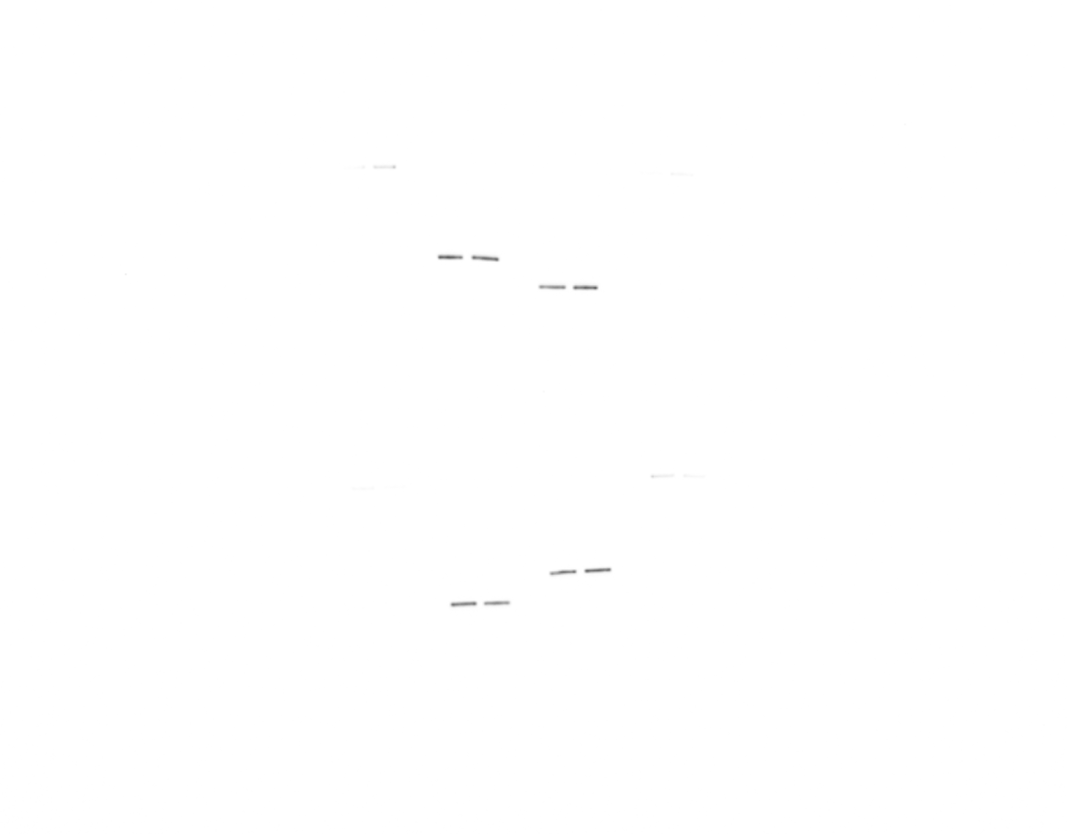

Supplement: Figure 2—figure supplement 1—source data 2. [file elife-90293-fig4-figsupp4-data4.zip › Figure 2, figure supplement 1 raw unedited/Figure 2, supplement 1A_2.tif]

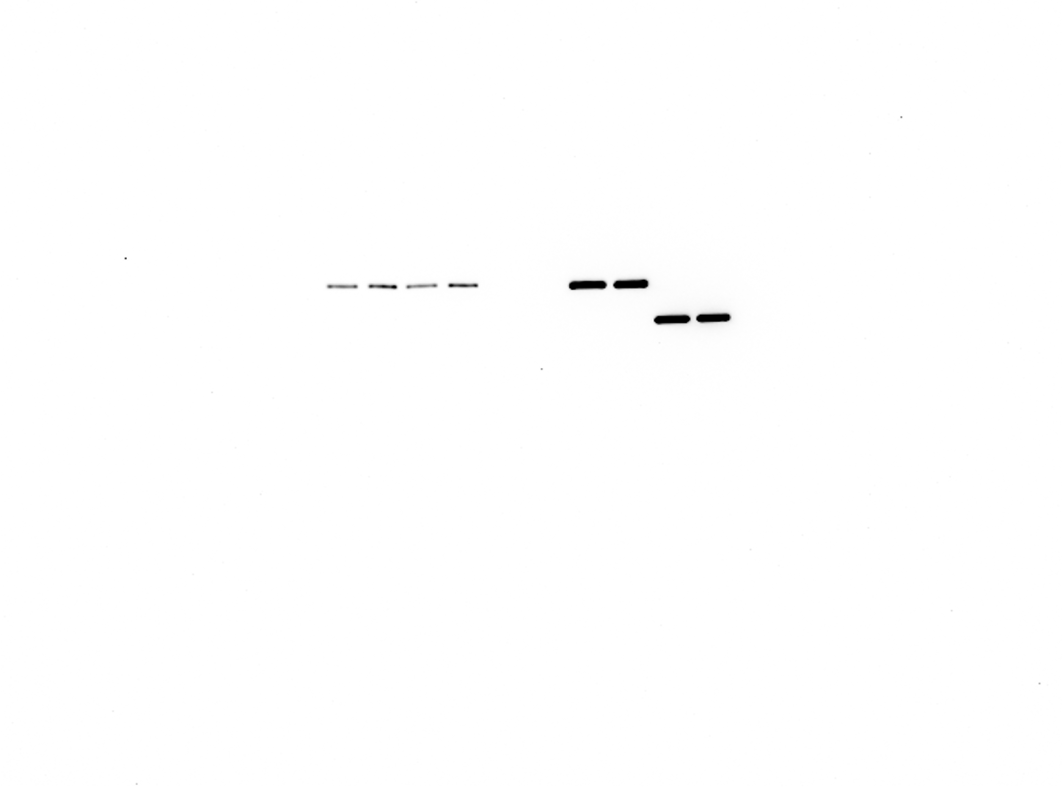

Supplement: Figure 2—figure supplement 1—source data 2. [file elife-90293-fig4-figsupp4-data4.zip › Figure 2, figure supplement 1 raw unedited/Figure 2, supplement 1A_1.tif]

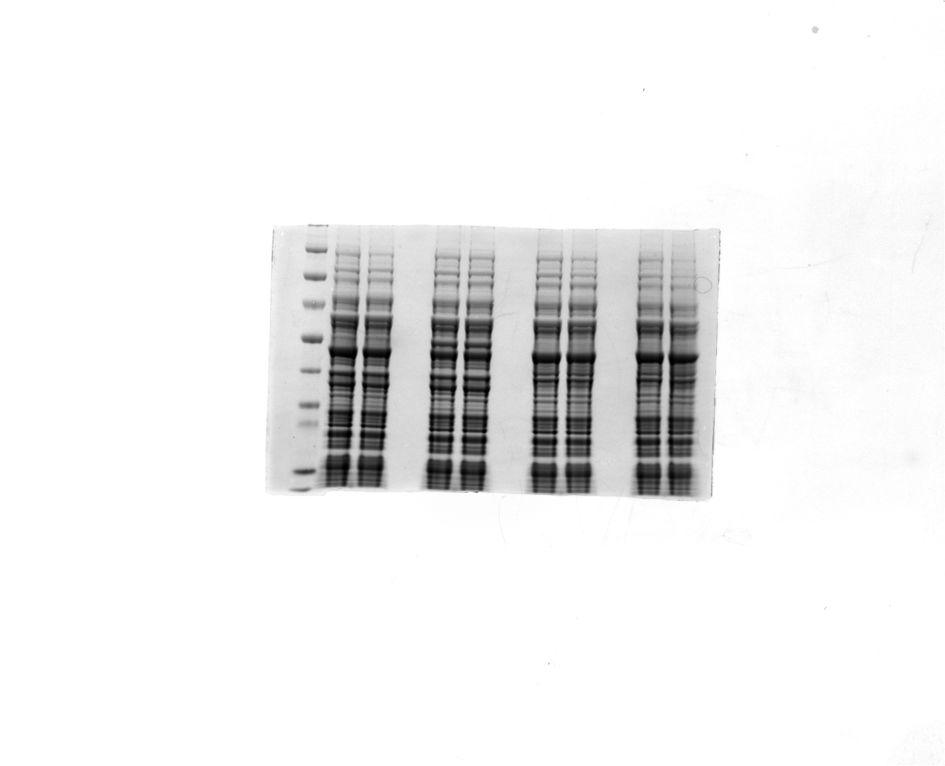

Supplement: Figure 2—figure supplement 1—source data 2. [file elife-90293-fig4-figsupp4-data4.zip › Figure 2, figure supplement 1 raw unedited/Figure 2, supplement 1A loading_1.tif]

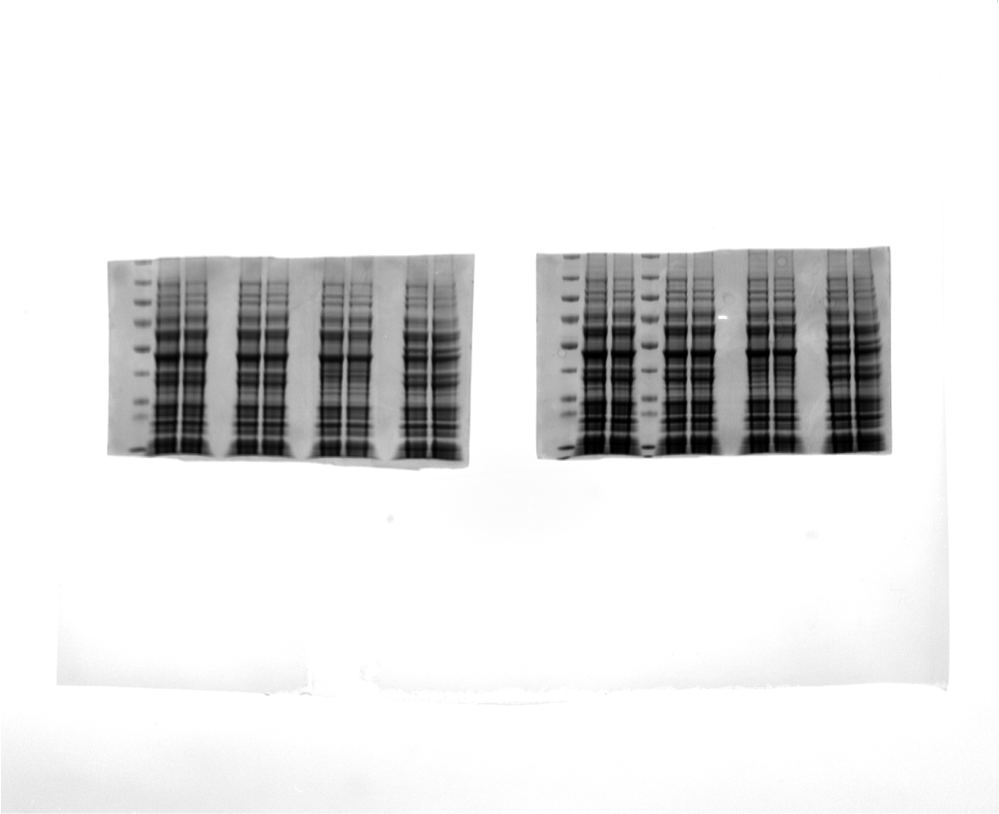

Supplement: Figure 2—figure supplement 1—source data 2. [file elife-90293-fig4-figsupp4-data4.zip › Figure 2, figure supplement 1 raw unedited/Figure 2, supplement 1A loading_2.tif]

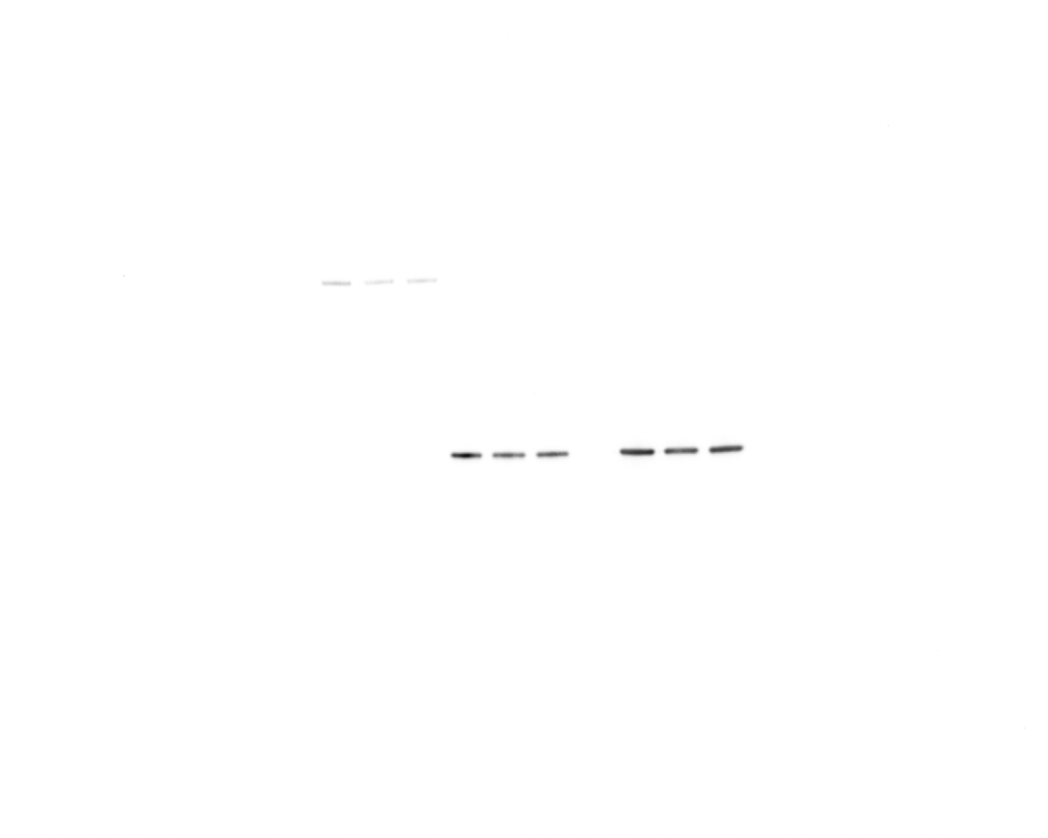

Supplement: Figure 2—figure supplement 1—source data 2. [file elife-90293-fig4-figsupp4-data4.zip › Figure 2, figure supplement 1 raw unedited/Figure 2, supplement 1B.tif]

**Figure 3D**-Pho12 and Pho84 levels in WT and *ubp3Δ* cells

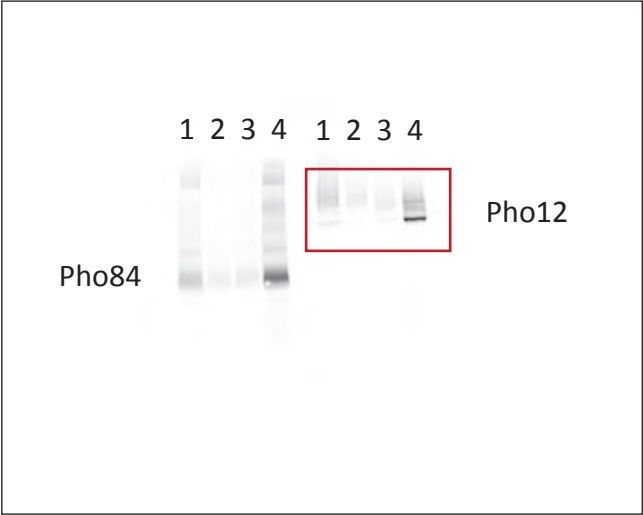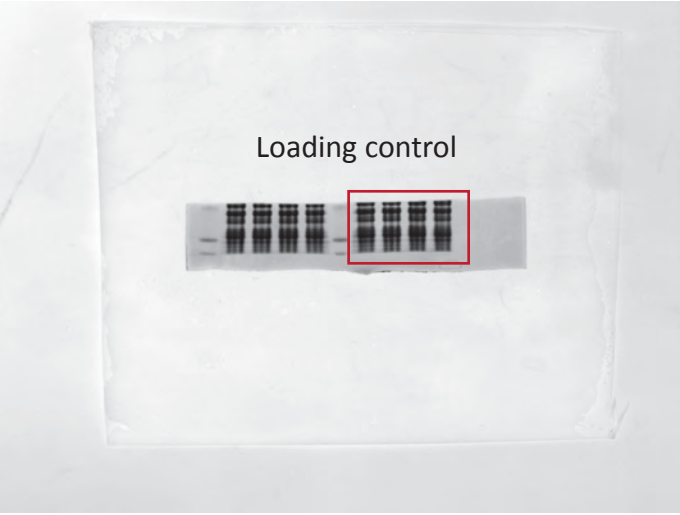

1- WT, 2- WT -High Pi, 3- *ubp3Δ*, 4- *ubp3Δ*- Pi

Supplement: Figure 3—source data 1. [file elife-90293-fig3-data1.zip › Figure 3/Figure 3-source data 1, uncropped and labelled gels.pdf]

**Figure 3D**-Pho12 and Pho84 levels in WT and *ubp3Δ* cells

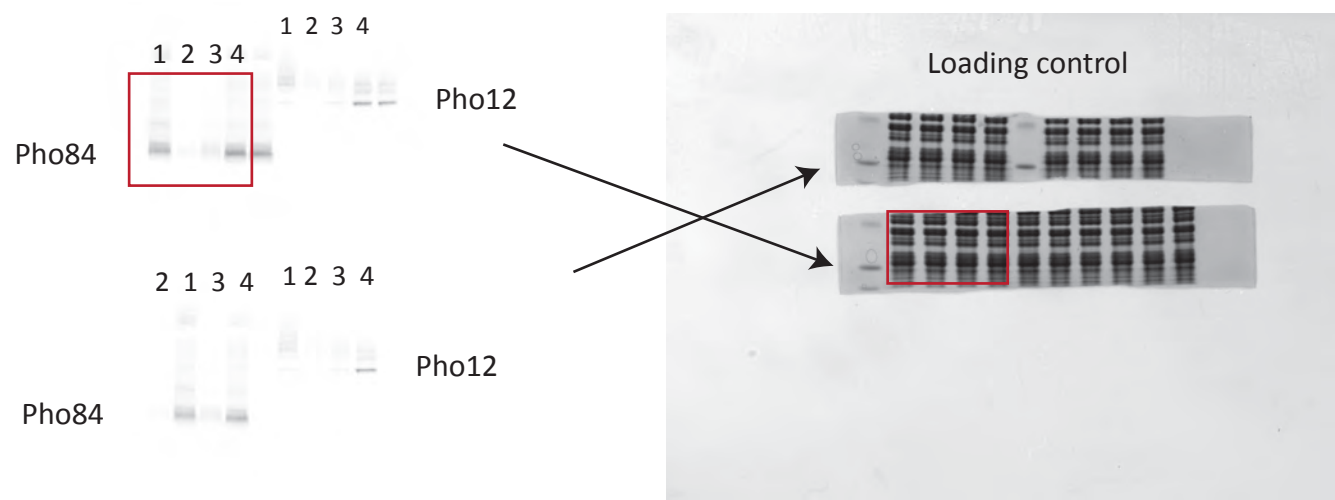

1- WT, 2- WT -High Pi, 3- *ubp3Δ*, 4- *ubp3Δ*- Pi

Supplement: Figure 3—source data 1. [file elife-90293-fig3-data1.zip › Figure 3/Figure 3-source data 2, uncropped and labelled gels.pdf]

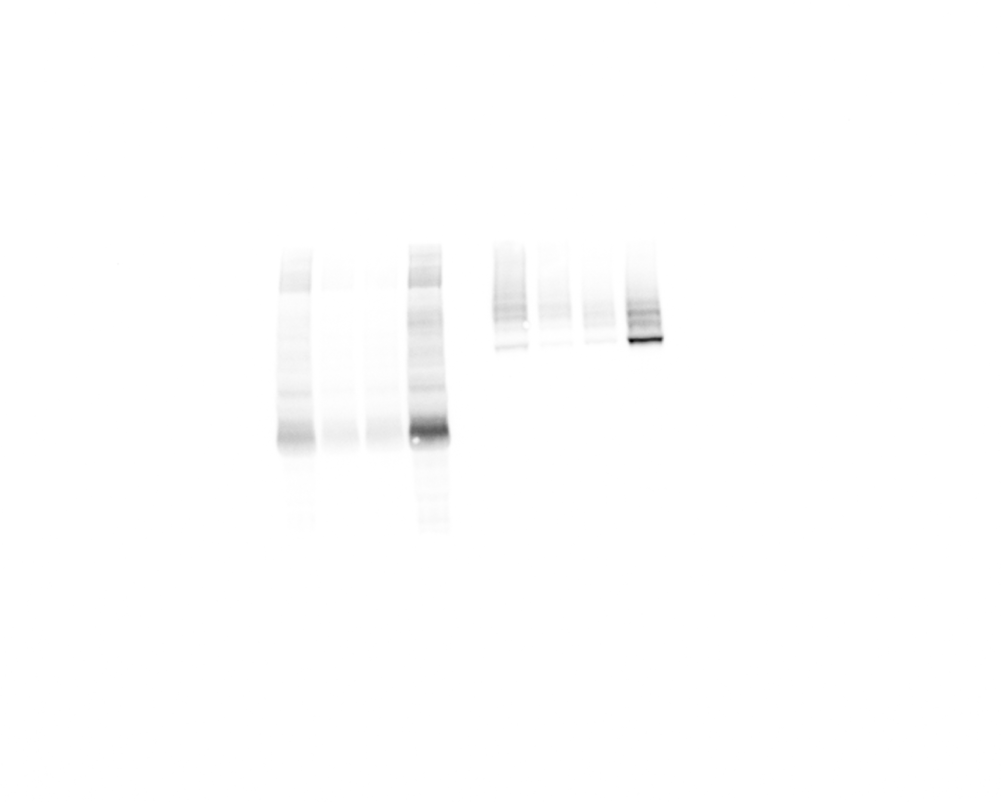

Supplement: Figure 3—source data 2. [file elife-90293-fig3-data2.zip › Figure 3 raw unedited/Figure 3D_1.tif]

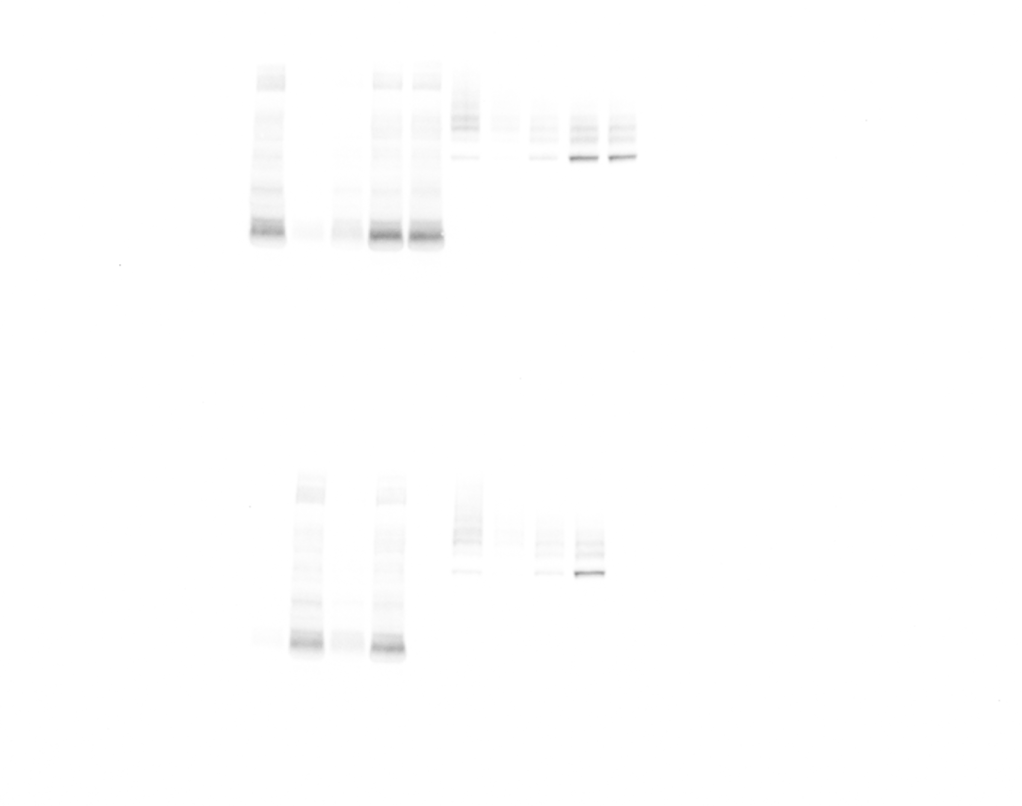

Supplement: Figure 3—source data 2. [file elife-90293-fig3-data2.zip › Figure 3 raw unedited/Figure 3D_2.tif]

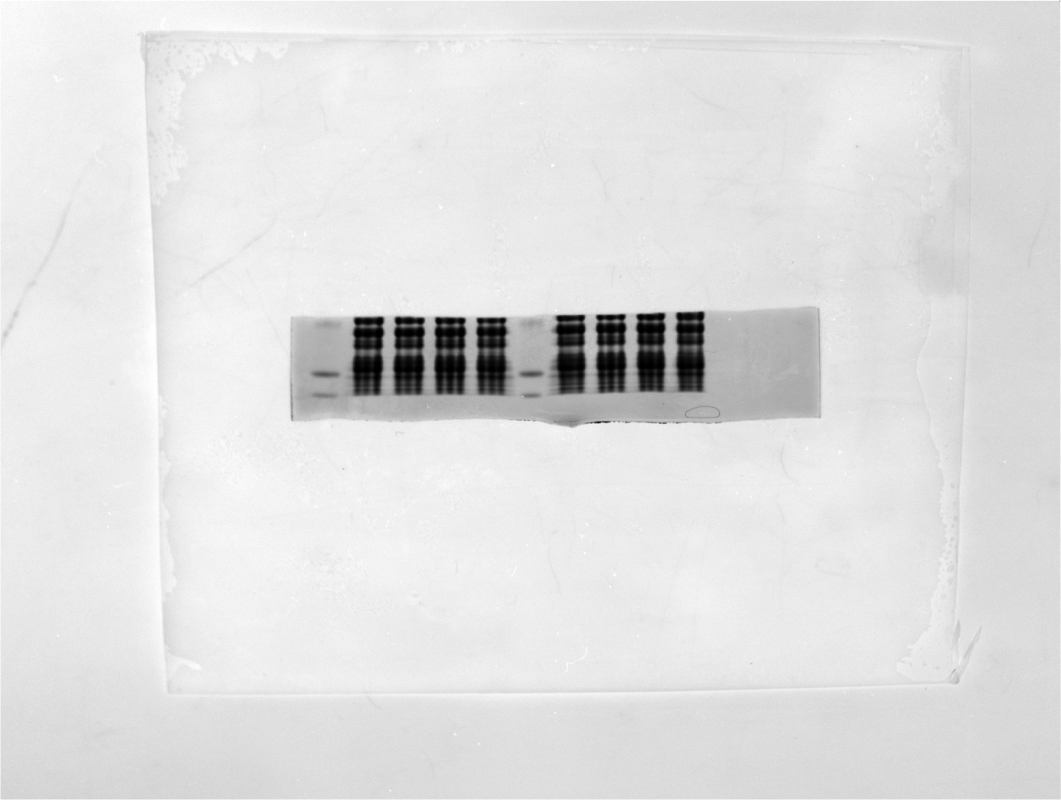

Supplement: Figure 3—source data 2. [file elife-90293-fig3-data2.zip › Figure 3 raw unedited/Figure 3D loading_1.tif]

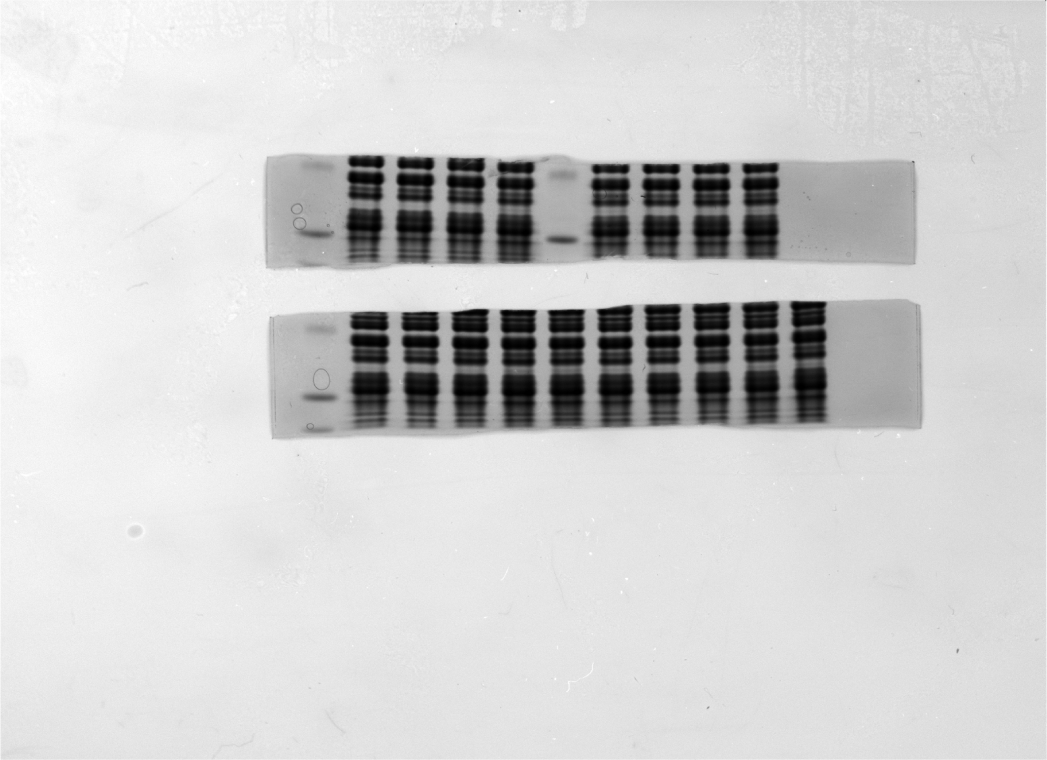

Supplement: Figure 3—source data 2. [file elife-90293-fig3-data2.zip › Figure 3 raw unedited/Figure 3D loading_2.tif]

**Figure 4G** -Cox 2 levels in WT,*ubp3Δ*, *mir1Δ* and *mir1Δubp3Δ* cells

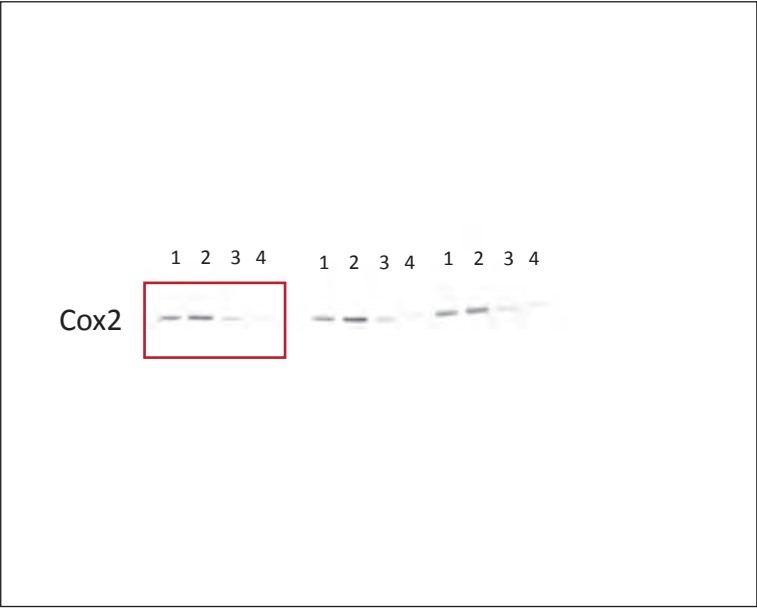

1-WT, 2- *ubp3Δ*, 3 - *mirΔ*, 4- *mirΔubp3Δ*

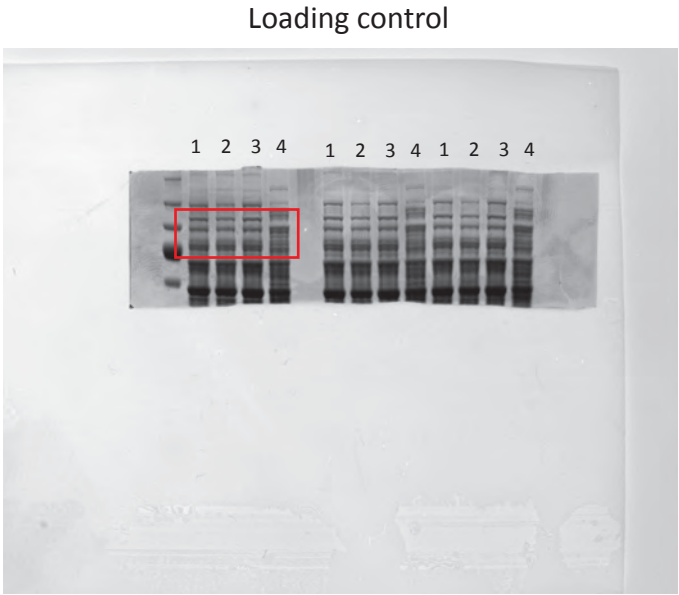

Supplement: Figure 4—source data 1. [file elife-90293-fig4-data1.zip › Figure 4/Figure 4-source data 2, uncropped and labelled gels.pdf]

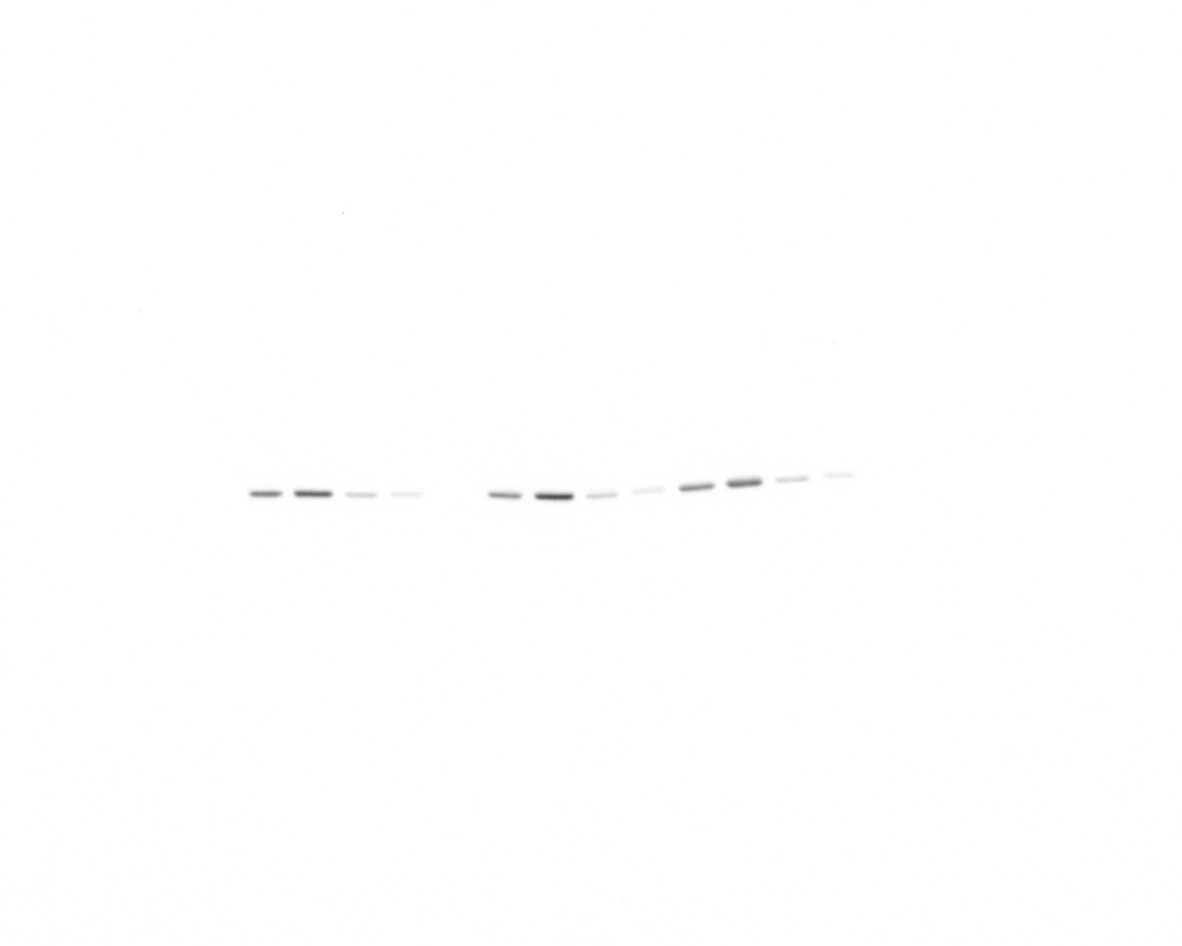

Supplement: Figure 4—source data 2. [file elife-90293-fig4-data2.zip › Figure 4 raw unedited/Figure 4G.tif]

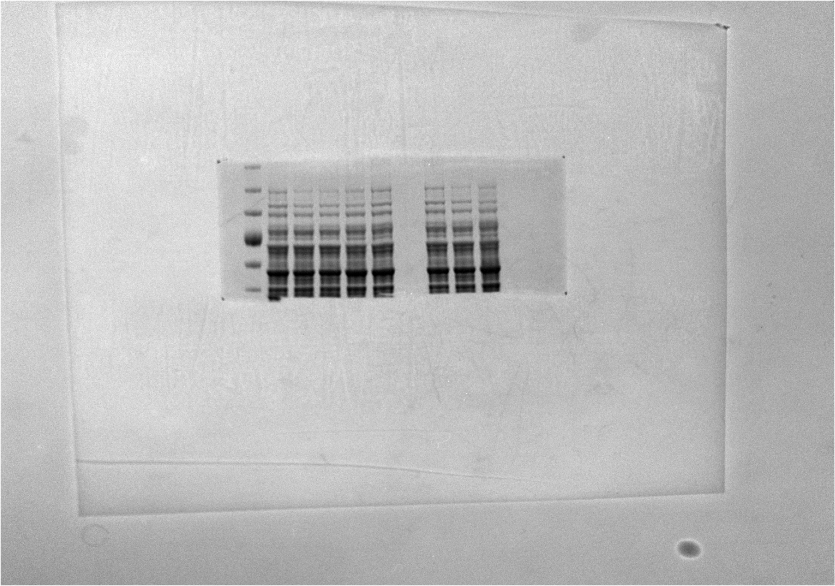

Supplement: Figure 4—source data 2. [file elife-90293-fig4-data2.zip › Figure 4 raw unedited/Figure 4C, 4E loading.tif]

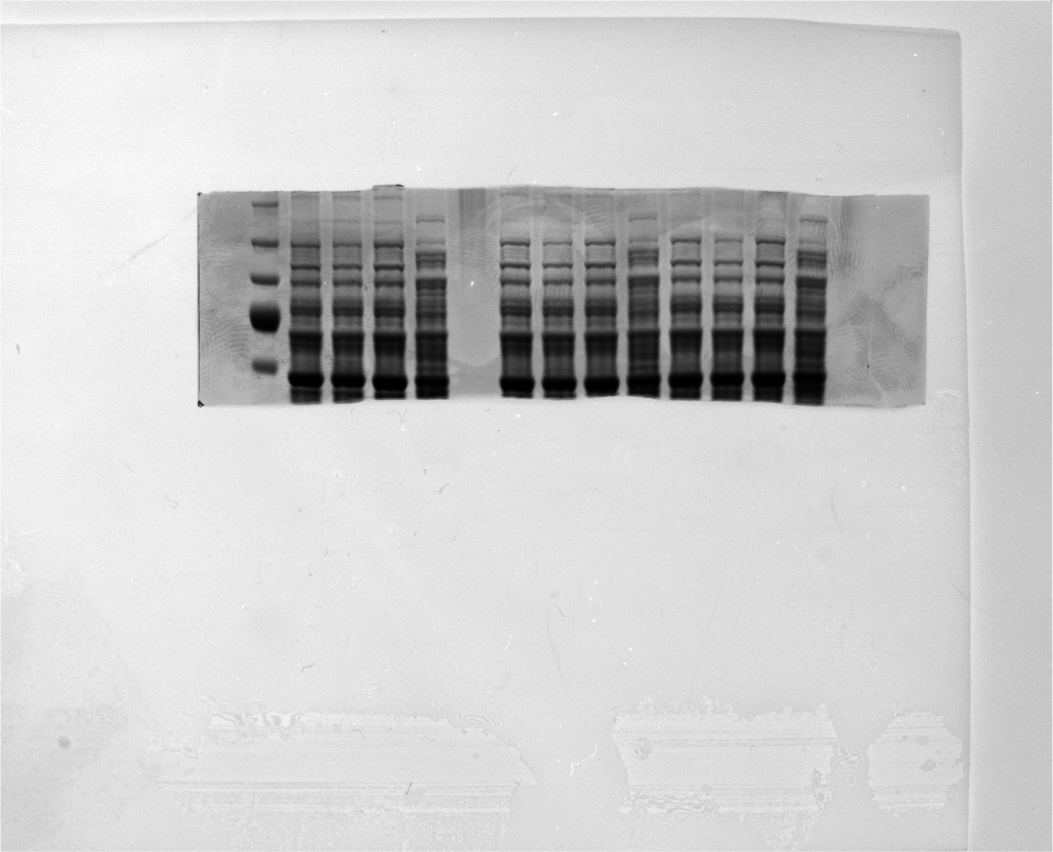

Supplement: Figure 4—source data 2. [file elife-90293-fig4-data2.zip › Figure 4 raw unedited/Figure 4G Loading.tif]

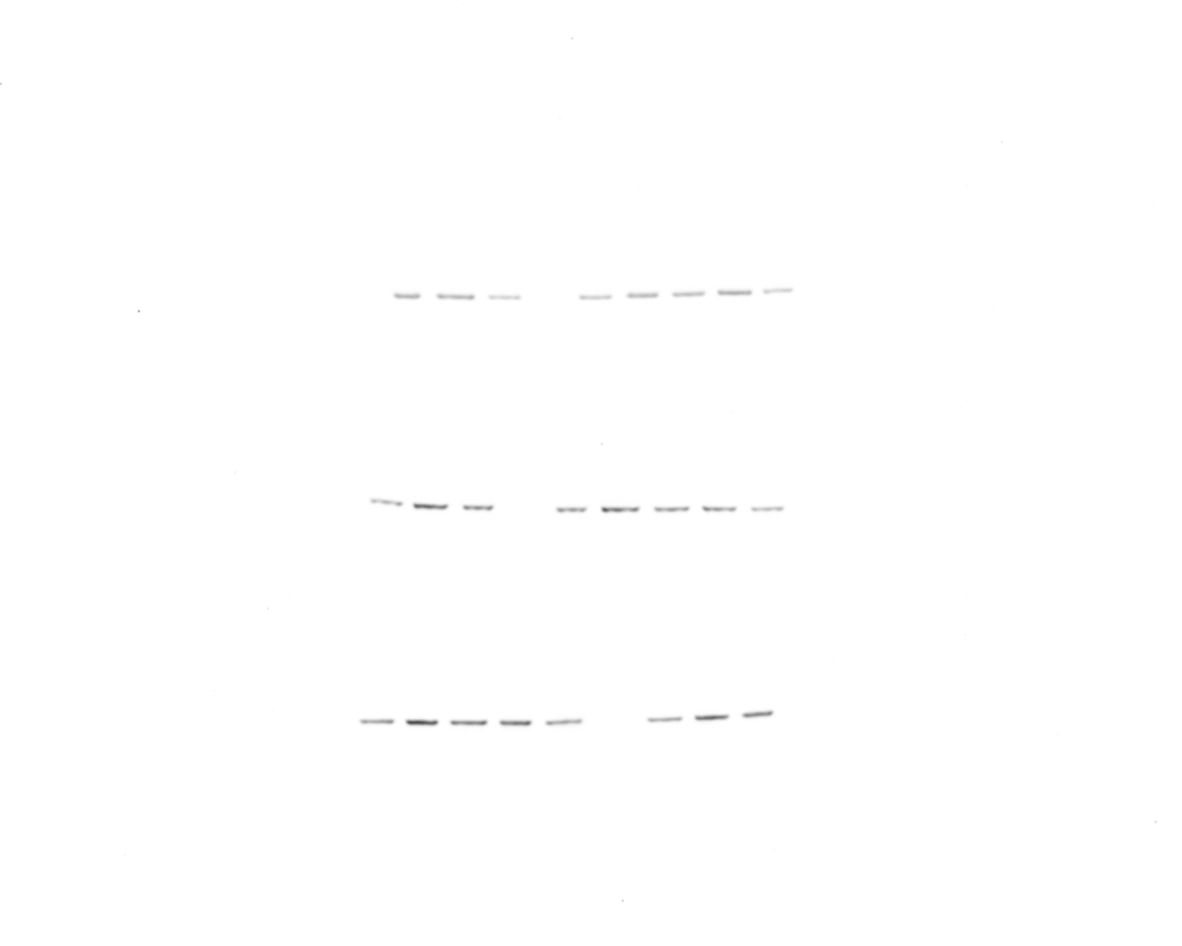

Supplement: Figure 4—source data 2. [file elife-90293-fig4-data2.zip › Figure 4 raw unedited/Figure 4C, 4E.tif]

Figure 4-figure supplement 1H-Mir1 and Pic2 levels

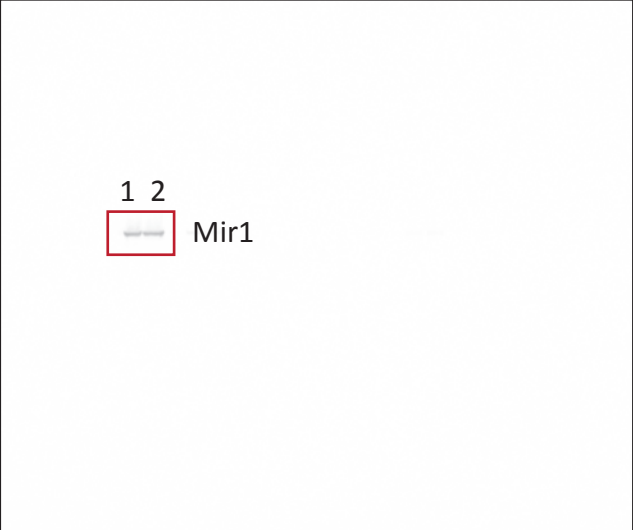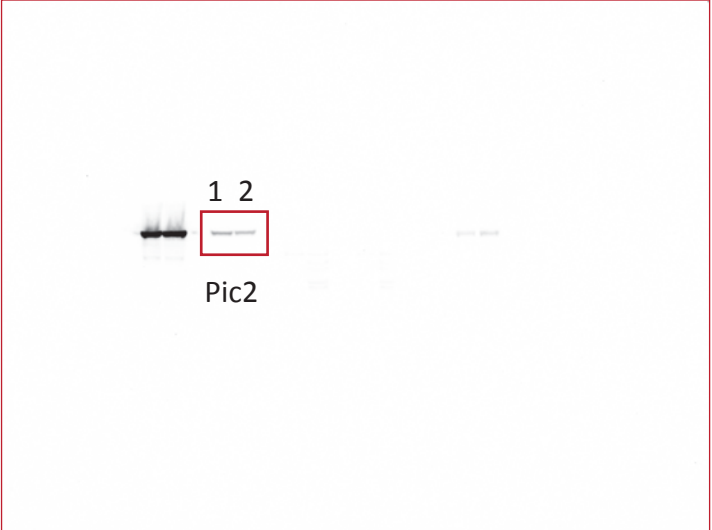

Loading

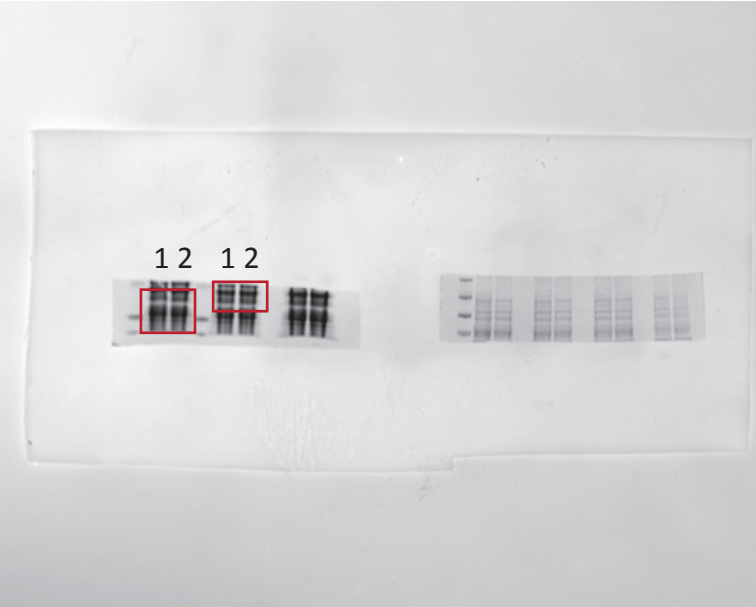

1 - WT  
2-ubp3

Supplement: Figure 4—figure supplement 1—source data 1. [file elife-90293-fig5-figsupp5-data5.zip › Figure 4,figure supplement 1/Figure 4, figure supplement 1-source data 4, uncropped and labelled gels.pdf]

**Figure 4-Figure supplement 1B - Idh1 in WT, WT+Pi, *ubp3Δ***

Loading control

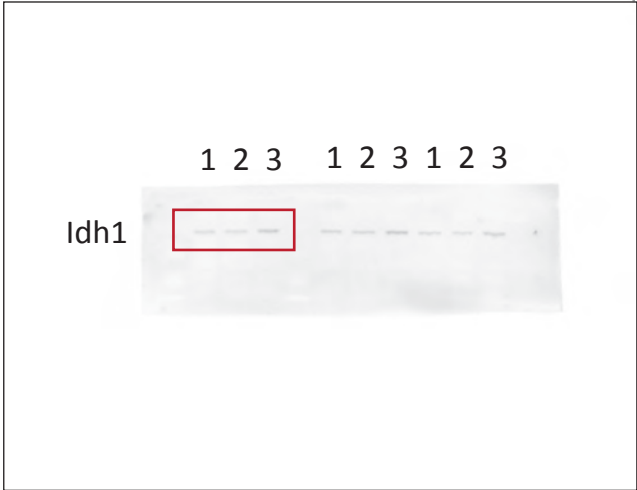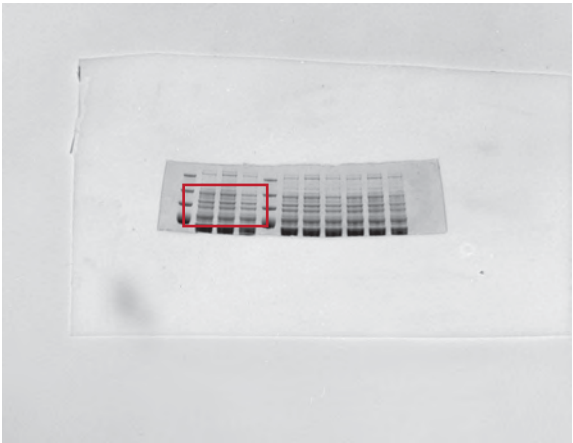

1-WT, 2- WT+Pi, 3- *ubp3Δ*

Supplement: Figure 4—figure supplement 1—source data 1. [file elife-90293-fig5-figsupp5-data5.zip › Figure 4,figure supplement 1/Figure 4, figure supplement 1-source data 3, uncropped and labelled gels.pdf]

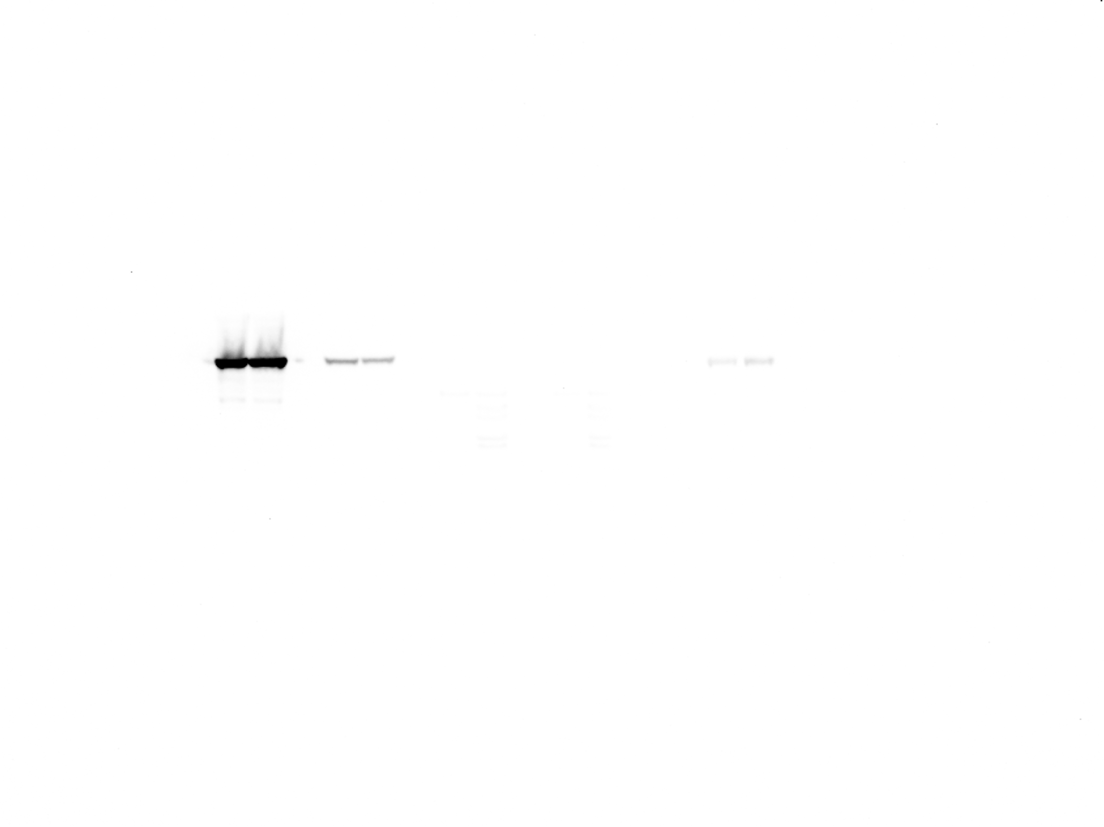

Supplement: Figure 4—figure supplement 1—source data 2. [file elife-90293-fig6-figsupp6-data6.zip › Figure 4, figure supplement 1 raw unedited/Figure 4, supplement 1H_2.tif]

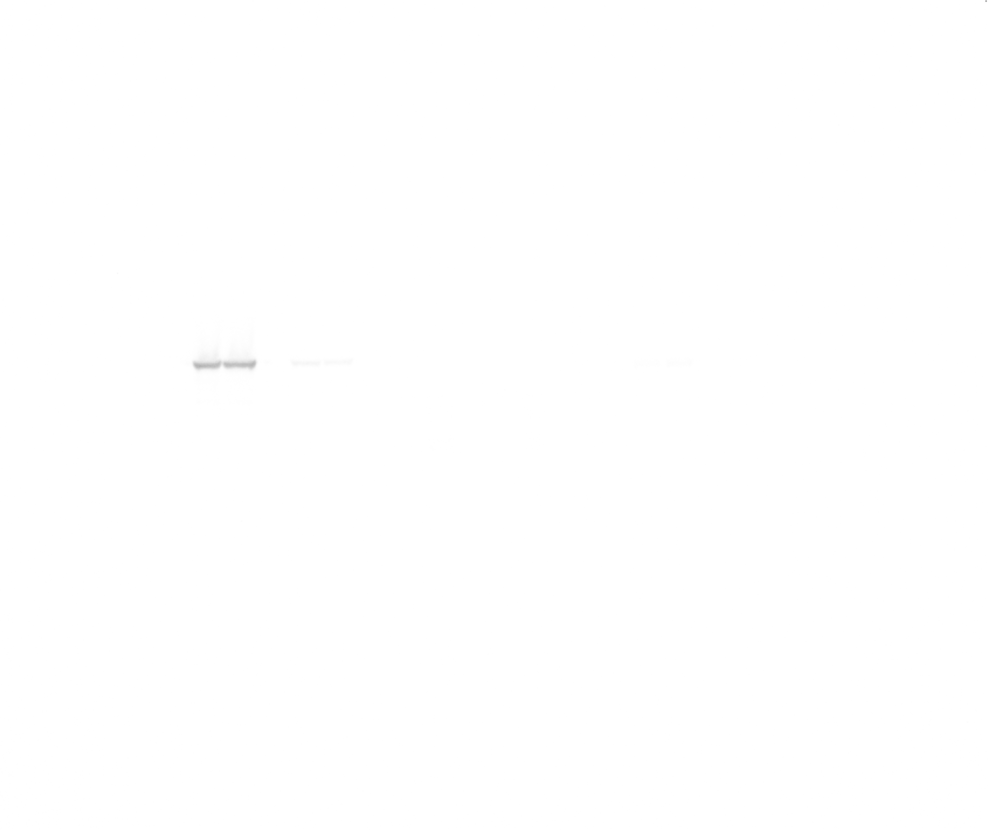

Supplement: Figure 4—figure supplement 1—source data 2. [file elife-90293-fig6-figsupp6-data6.zip › Figure 4, figure supplement 1 raw unedited/Figure 4, supplement 1H_1.tif]

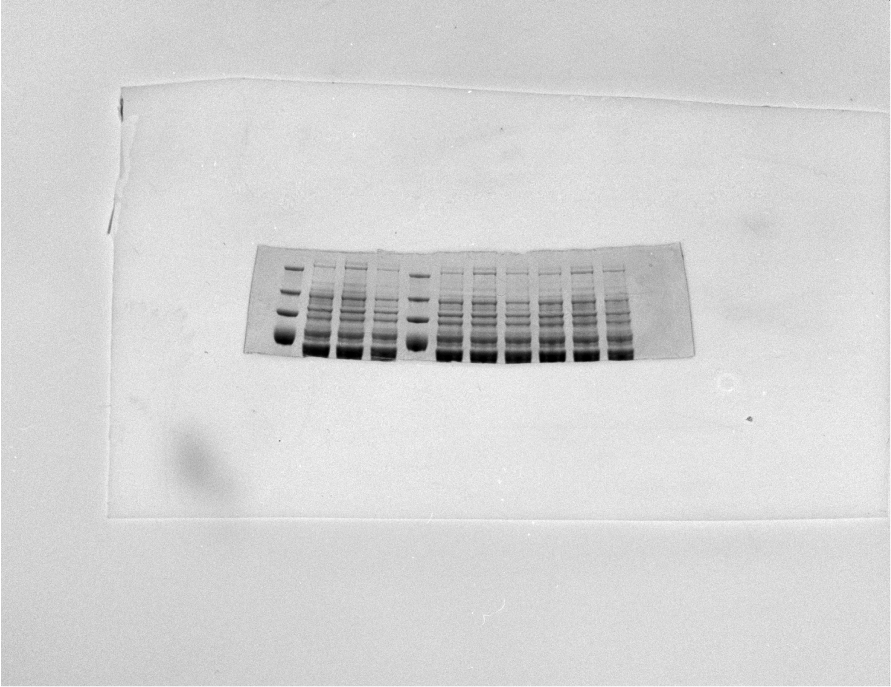

Supplement: Figure 4—figure supplement 1—source data 2. [file elife-90293-fig6-figsupp6-data6.zip › Figure 4, figure supplement 1 raw unedited/Figure 4, supplement 1B_loading.tif]

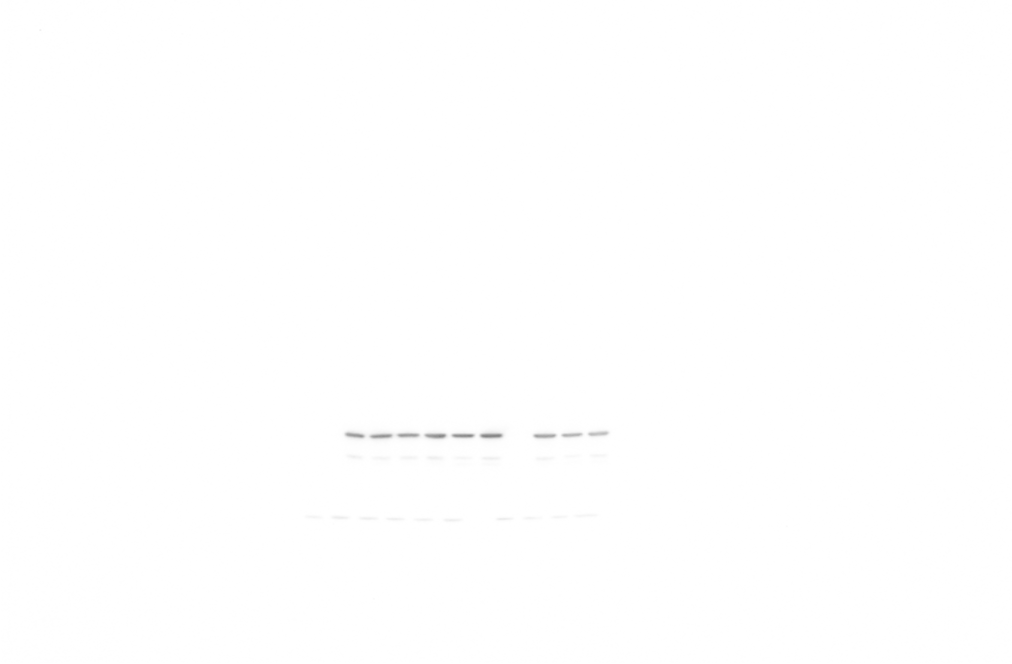

Supplement: Figure 4—figure supplement 1—source data 2. [file elife-90293-fig6-figsupp6-data6.zip › Figure 4, figure supplement 1 raw unedited/Figure 4, supplement 1A_1.tif]

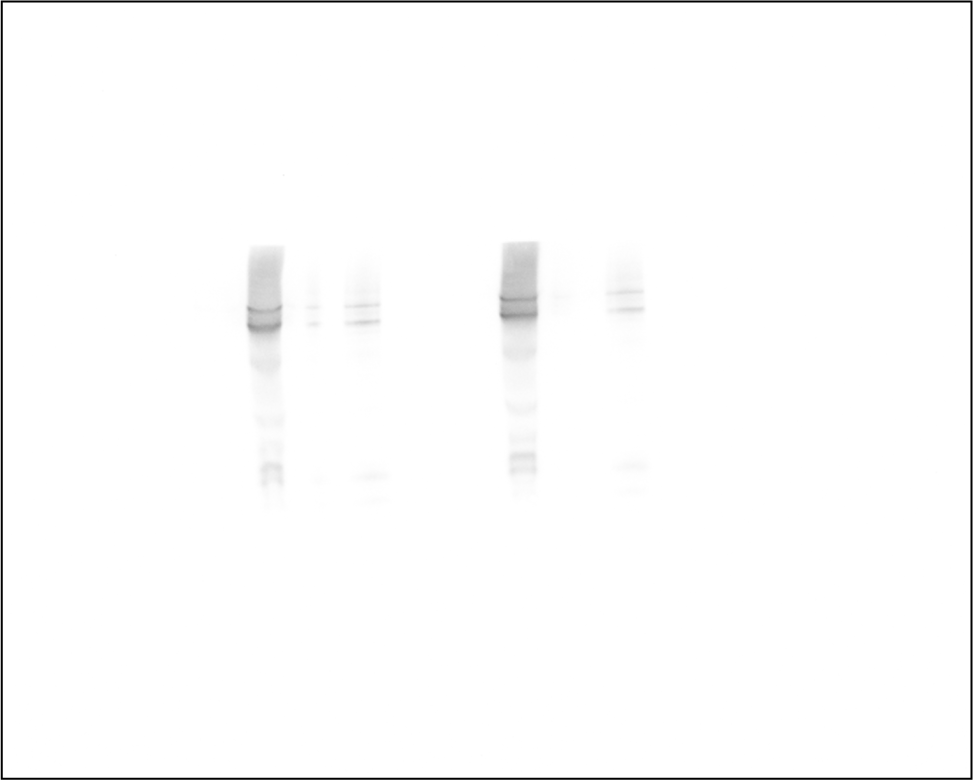

Supplement: Figure 4—figure supplement 1—source data 2. [file elife-90293-fig6-figsupp6-data6.zip › Figure 4, figure supplement 1 raw unedited/Figure 4, supplement 1A_3.tif]

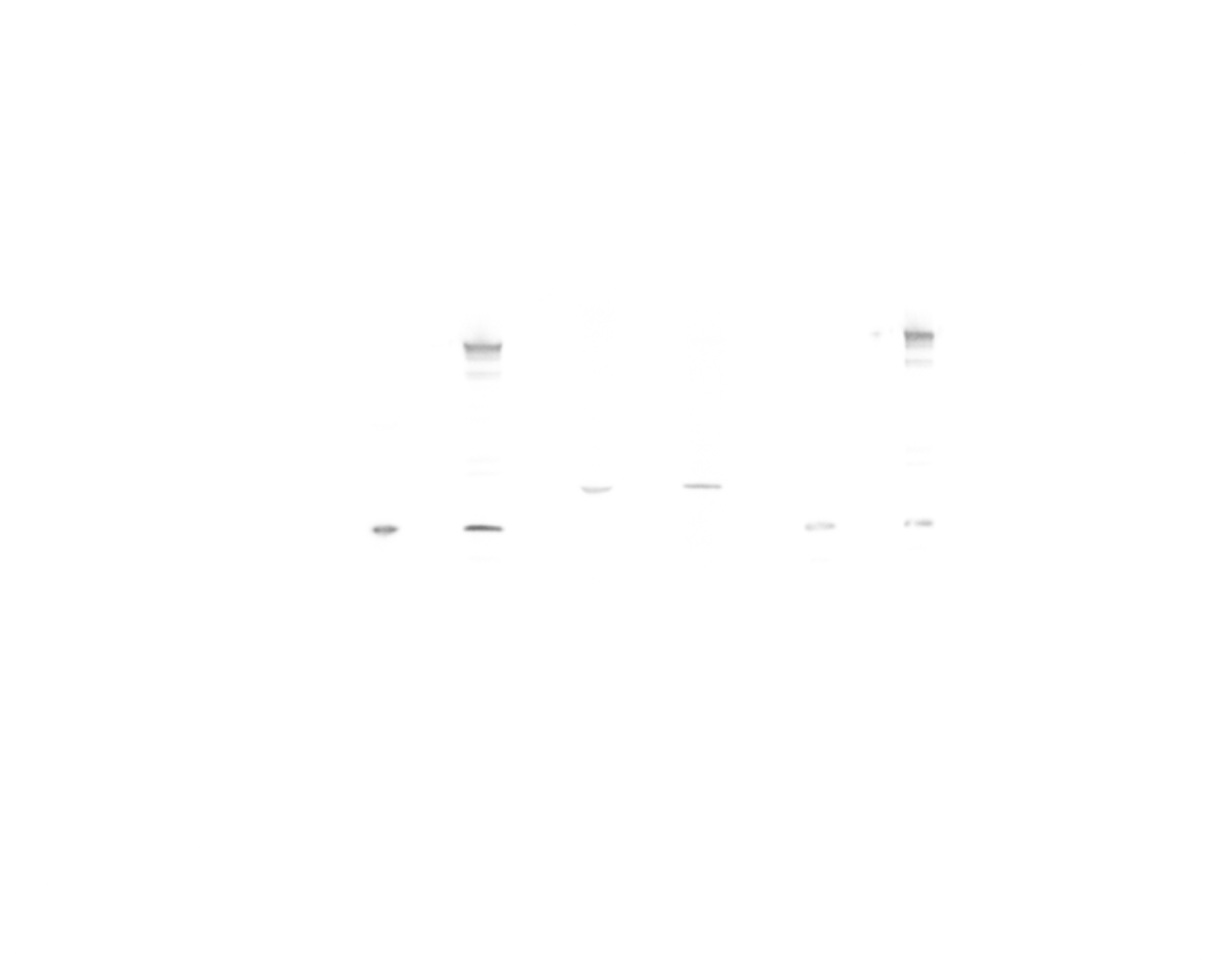

Supplement: Figure 4—figure supplement 1—source data 2. [file elife-90293-fig6-figsupp6-data6.zip › Figure 4, figure supplement 1 raw unedited/Figure 4, supplement 1A_2.tif]

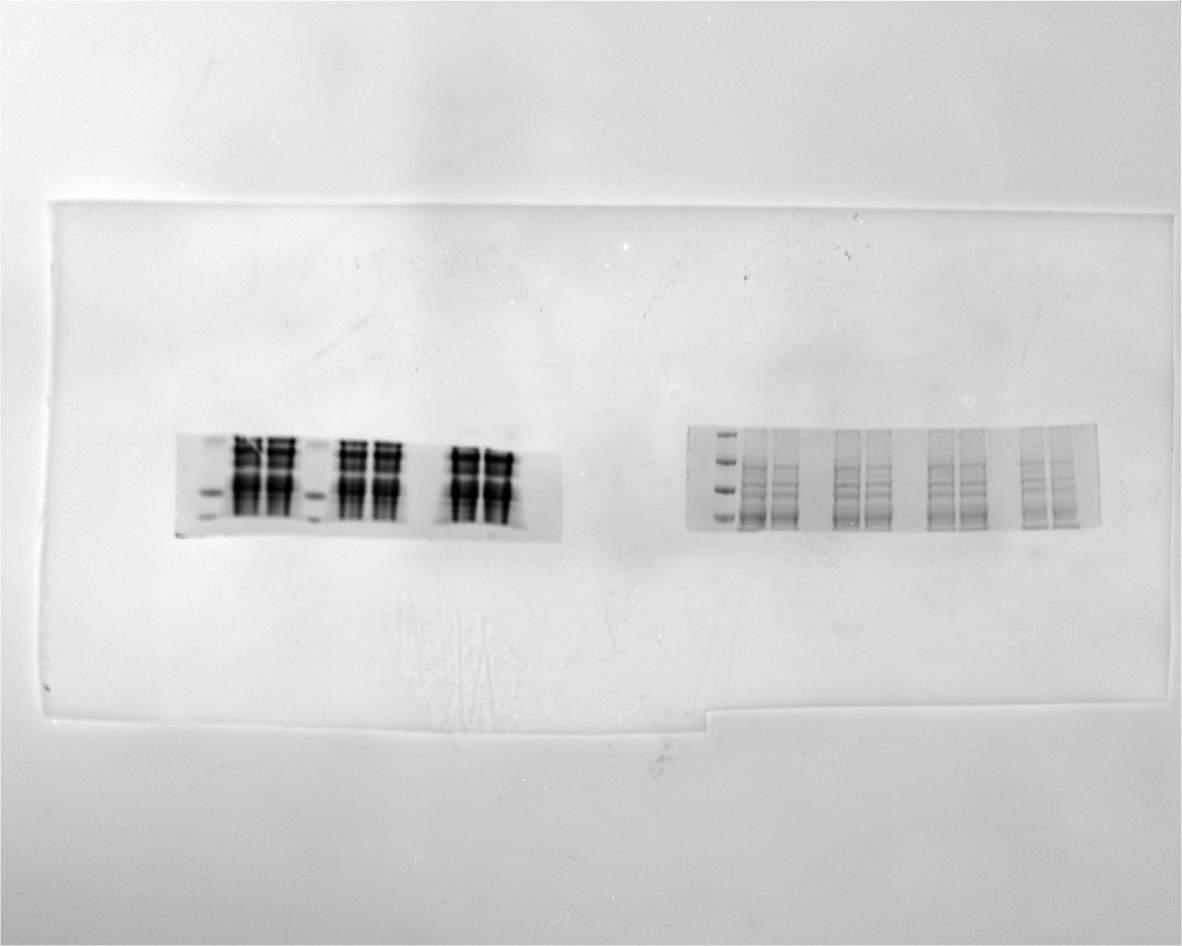

Supplement: Figure 4—figure supplement 1—source data 2. [file elife-90293-fig6-figsupp6-data6.zip › Figure 4, figure supplement 1 raw unedited/Figure 4, supplement 1H_loading.tif]

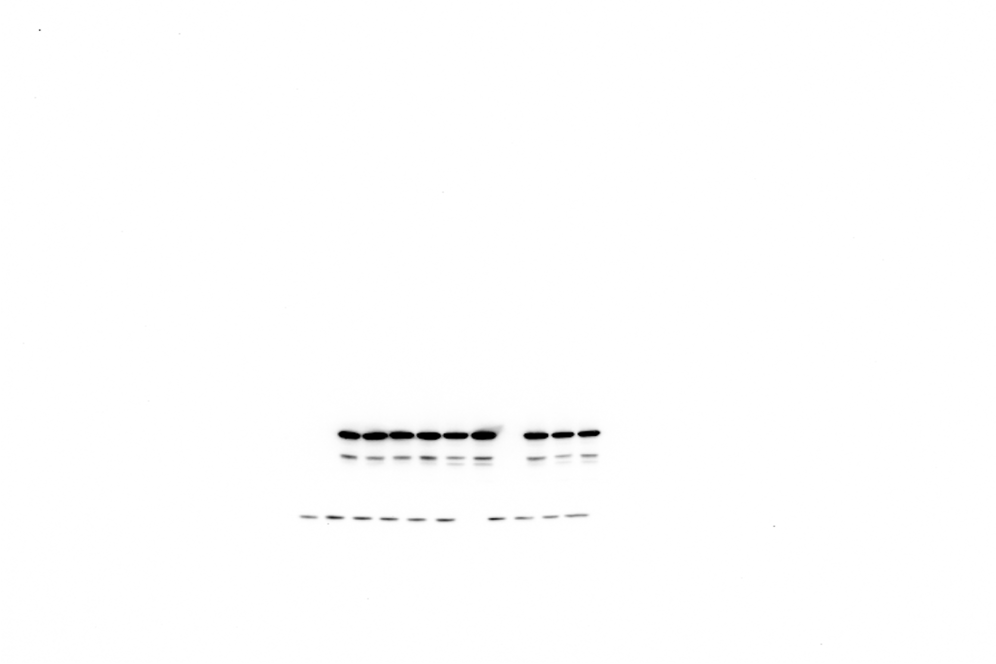

Supplement: Figure 4—figure supplement 1—source data 2. [file elife-90293-fig6-figsupp6-data6.zip › Figure 4, figure supplement 1 raw unedited/Figure 4, supplement 1A_1.1.tif]

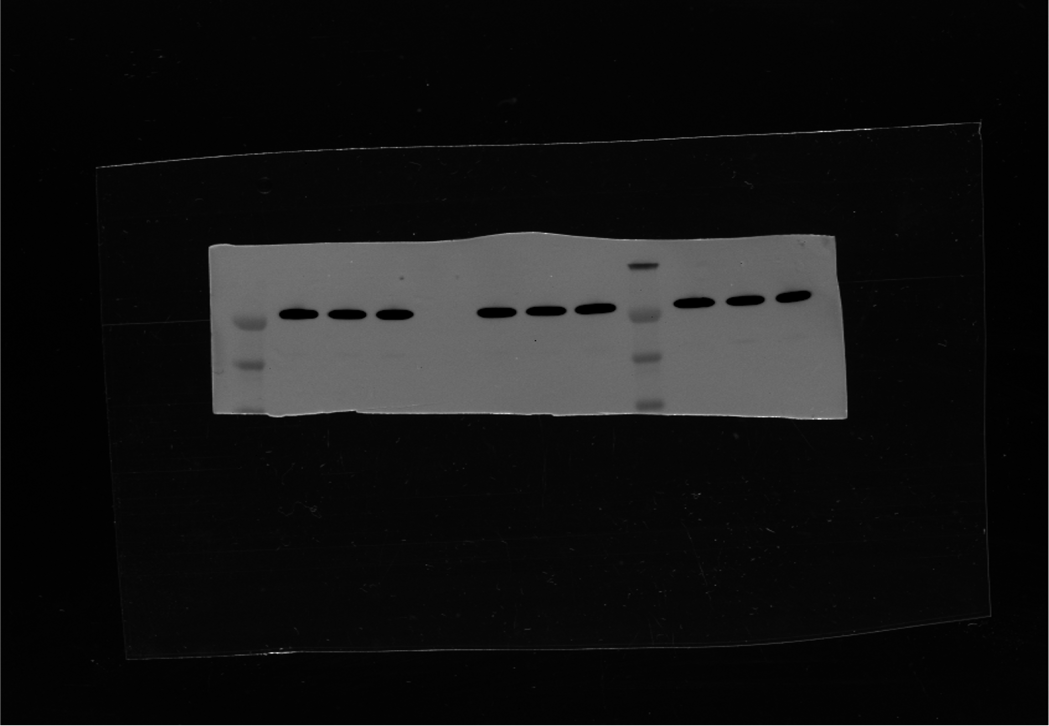

Supplement: Figure 4—figure supplement 1—source data 2. [file elife-90293-fig6-figsupp6-data6.zip › Figure 4, figure supplement 1 raw unedited/Figure 4, supplement 1A_1.2.tif]

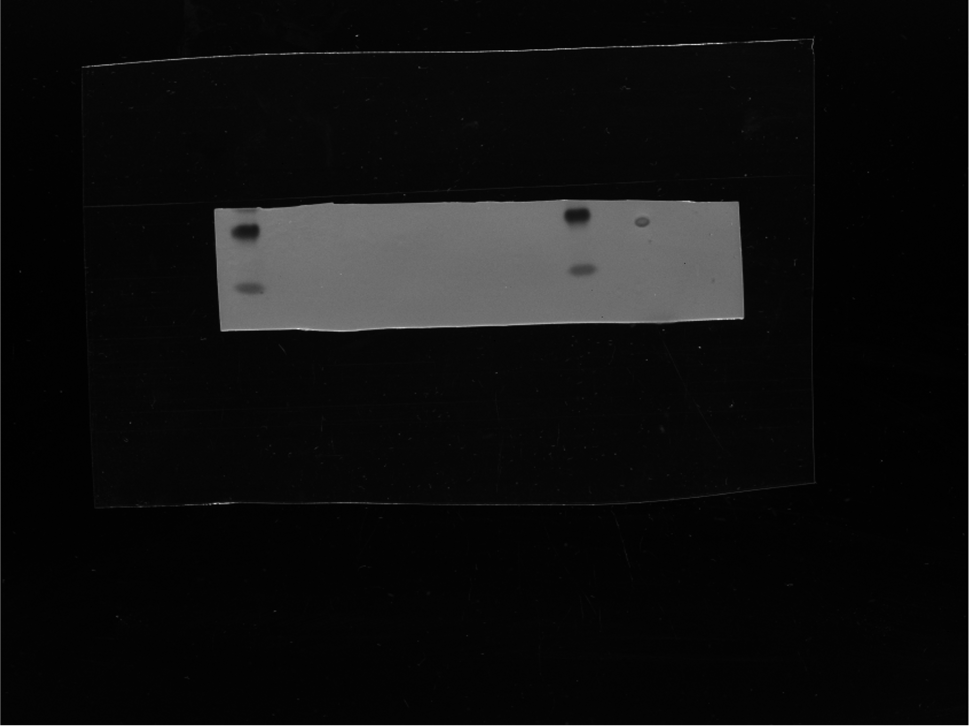

Supplement: Figure 4—figure supplement 1—source data 2. [file elife-90293-fig6-figsupp6-data6.zip › Figure 4, figure supplement 1 raw unedited/Figure 4, supplement 1A_1.3.tif]

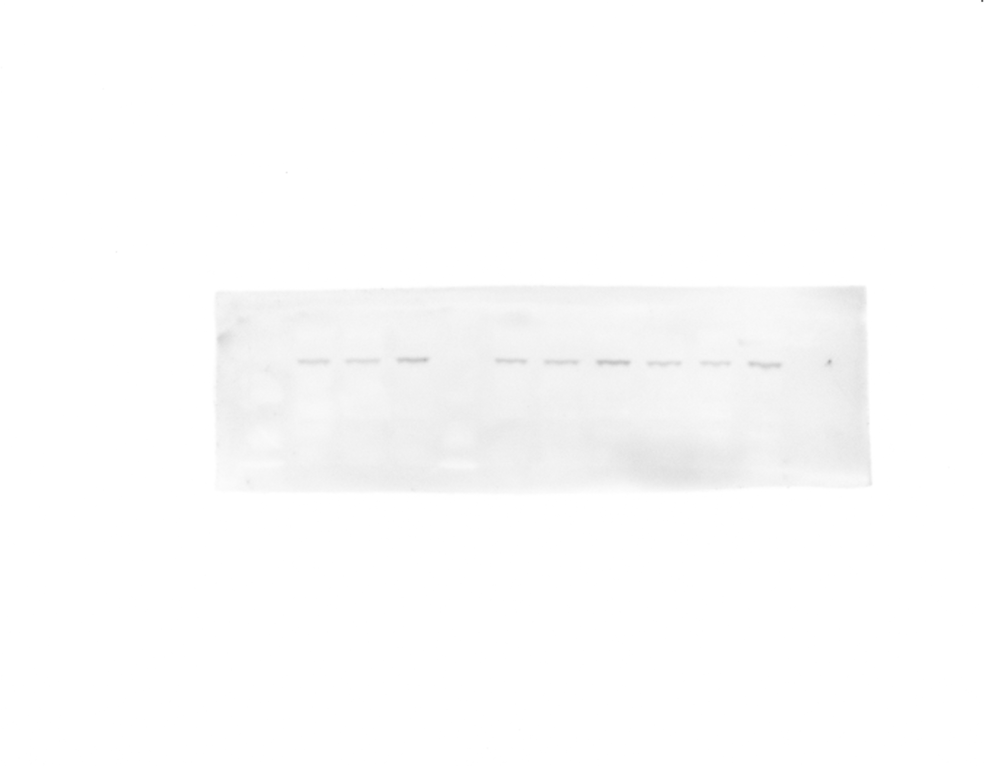

Supplement: Figure 4—figure supplement 1—source data 2. [file elife-90293-fig6-figsupp6-data6.zip › Figure 4, figure supplement 1 raw unedited/Figure 4, supplement 1B.tif]

Figure 5C- Mir1 levels in High and low glucose

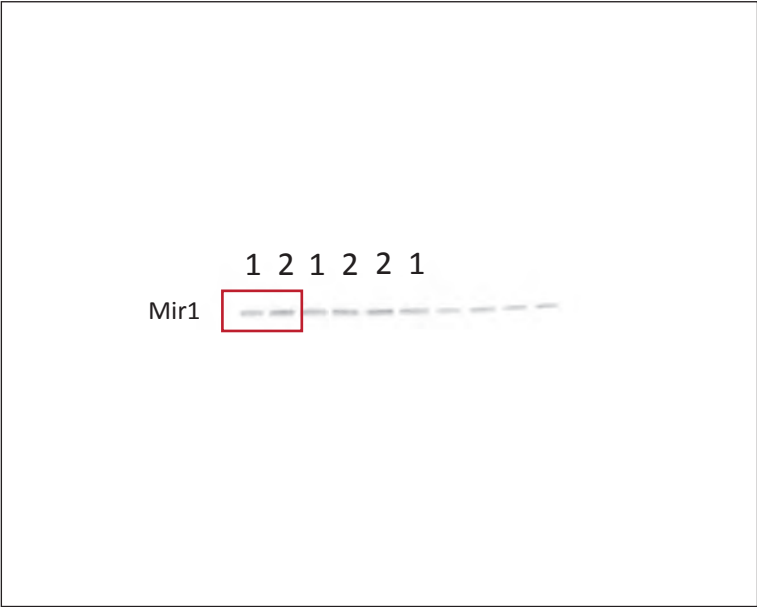

Loading control

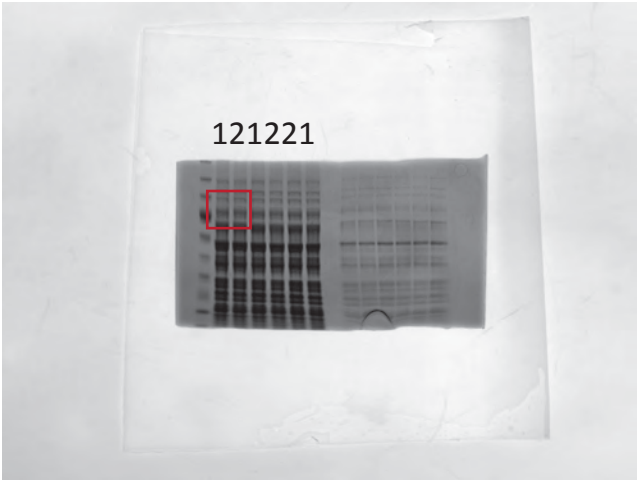

1-2% glc, 2- 0.1% glc

Supplement: Figure 5—source data 1. [file elife-90293-fig5-data1.zip › Figure 5/Figure 5-source data 1, uncropped and labelled gels.pdf]

Figure 5E-Cox2 levels in Mir1 OE

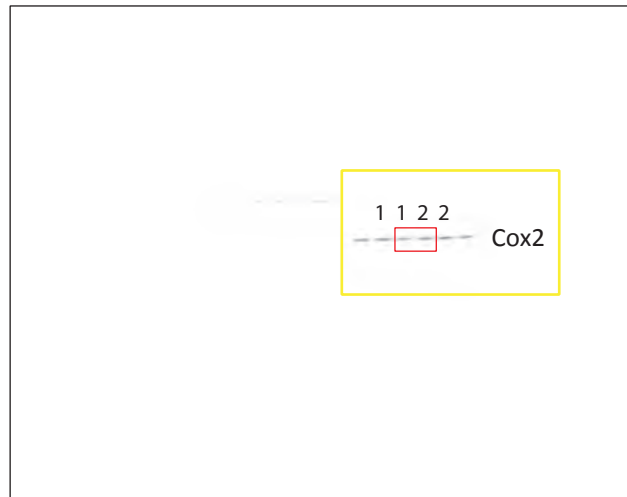

Loading control

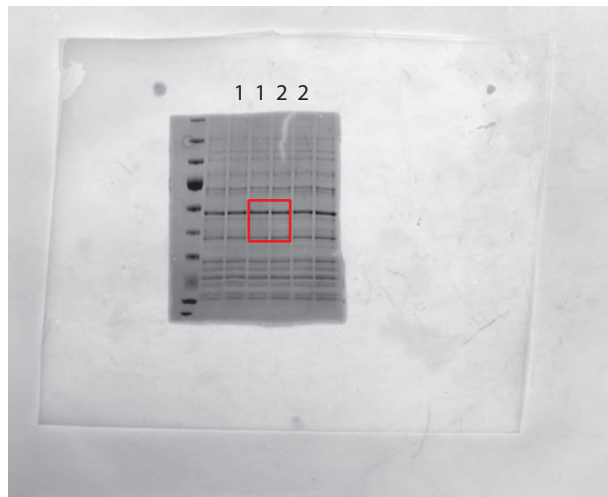

Supplement: Figure 5—source data 1. [file elife-90293-fig5-data1.zip › Figure 5/Figure 5-source data 2, uncropped and labelled gels.pdf]

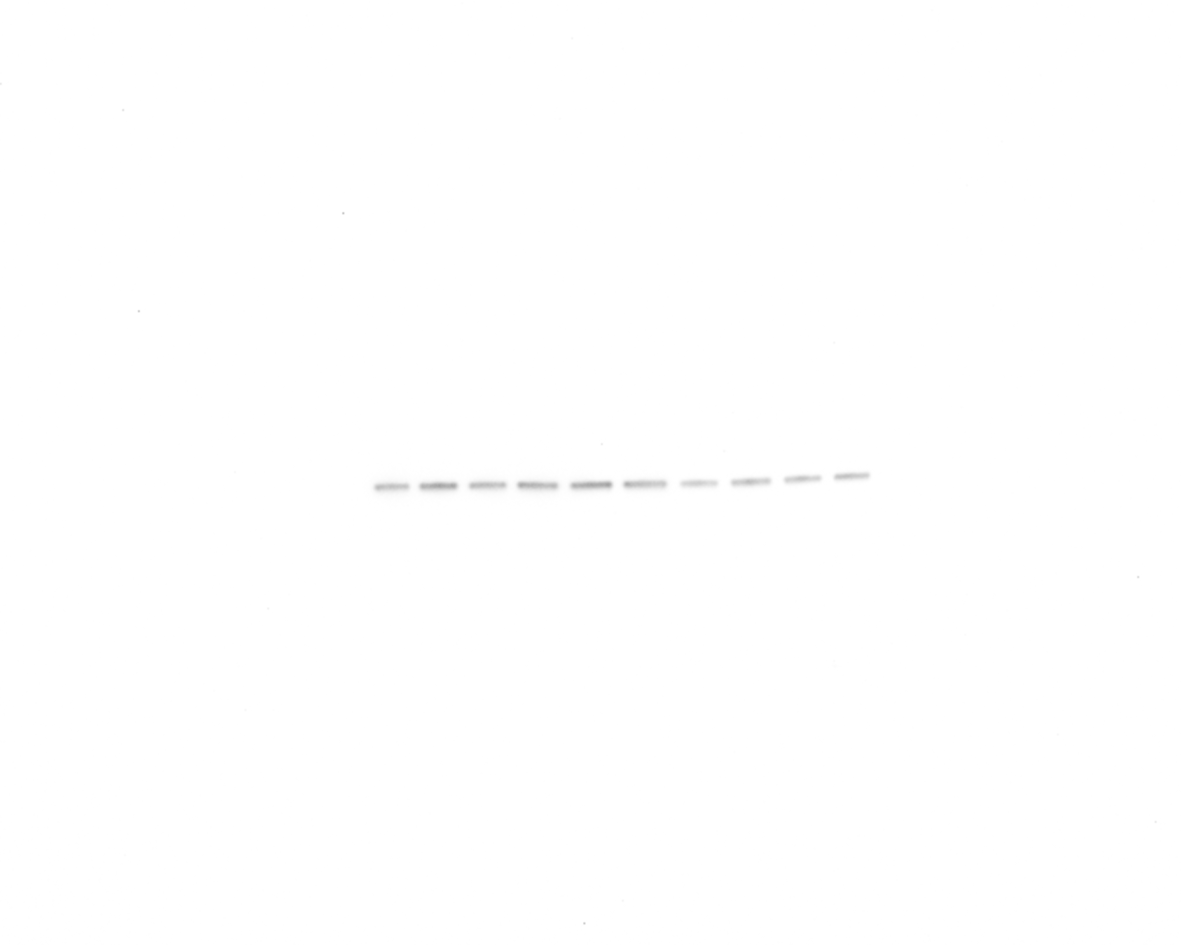

Supplement: Figure 5—source data 2. [file elife-90293-fig5-data2.zip › Figure 5 raw unedited/Figure 5C.tif]

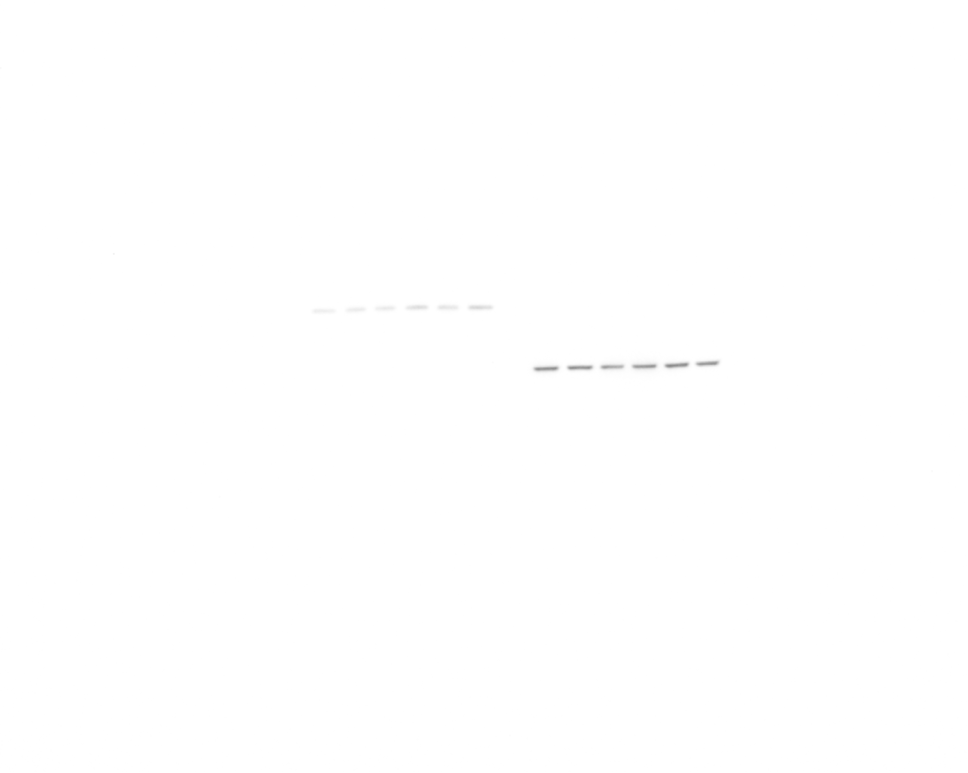

Supplement: Figure 5—source data 2. [file elife-90293-fig5-data2.zip › Figure 5 raw unedited/Figure 5E.tif]

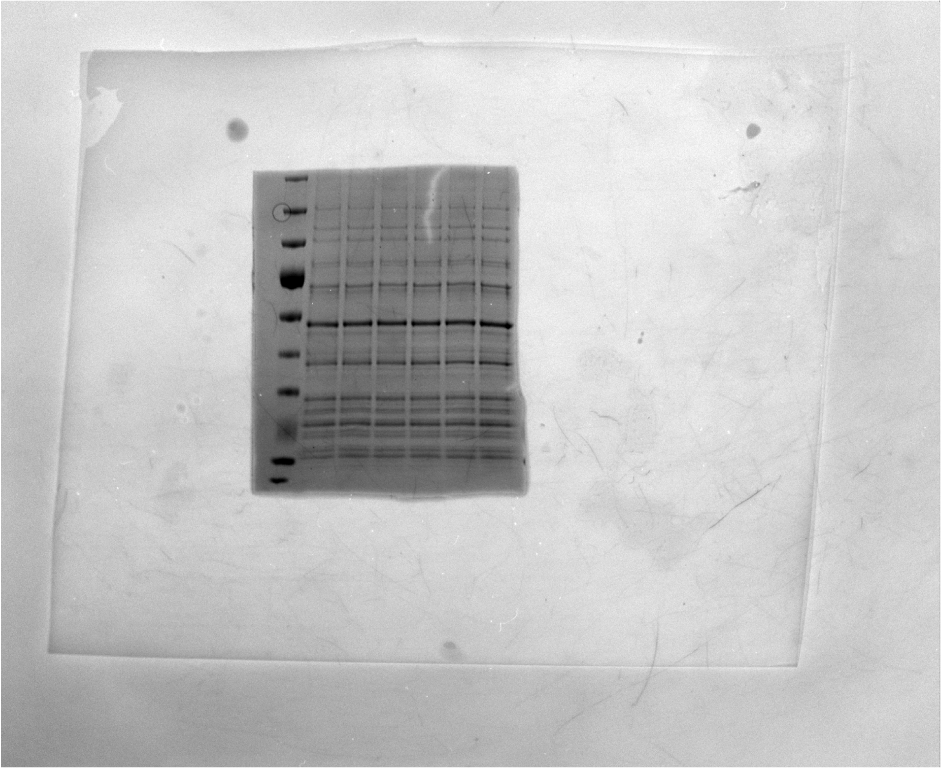

Supplement: Figure 5—source data 2. [file elife-90293-fig5-data2.zip › Figure 5 raw unedited/Figure 5E loading.tif]

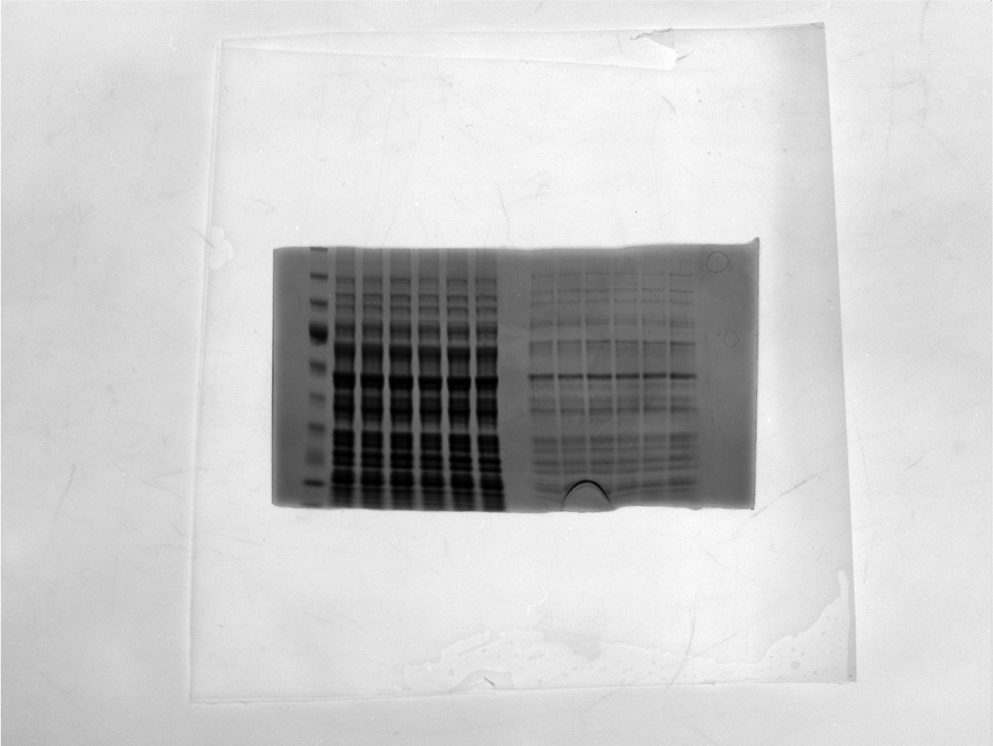

Supplement: Figure 5—source data 2. [file elife-90293-fig5-data2.zip › Figure 5 raw unedited/Figure 5C loading.tif]

Figure 5-figure supplement 1B-Mir1 levels in glucose and ethanol

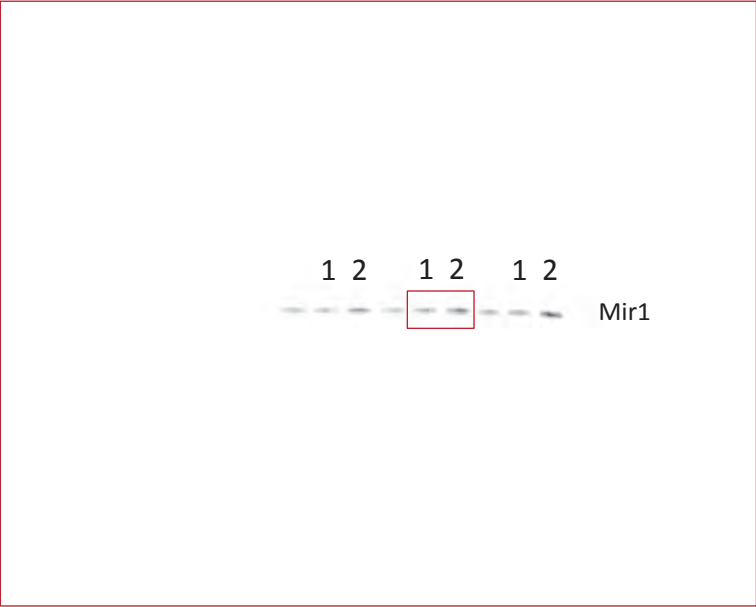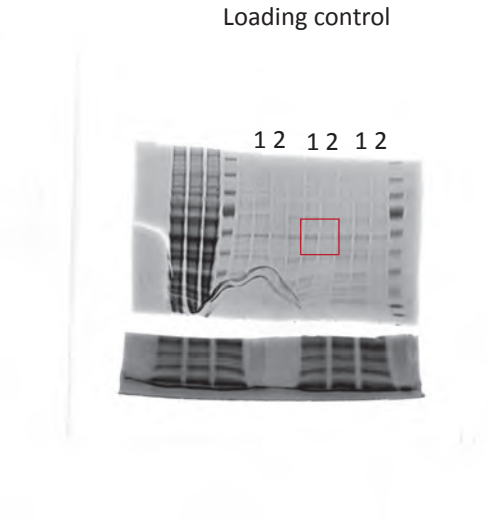

1-glucose 2-ethanol

Supplement: Figure 5—figure supplement 1—source data 1. [file elife-90293-fig5-figsupp1-data1.zip › Figure 5,figure supplement 1/Figure 5, figure supplement 1-source data 1, uncropped and labelled gels.pdf]

Figure 5-figure supplement 1K-Mpc3 levels in WT, Mir10E

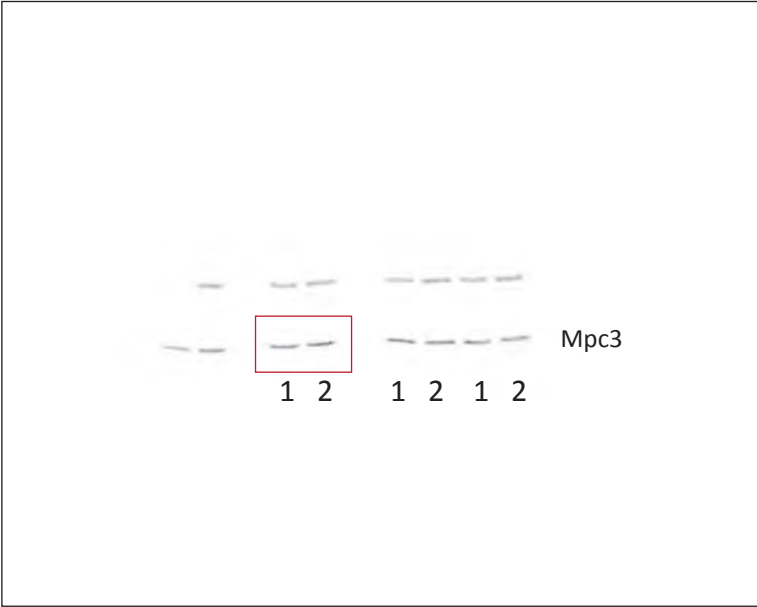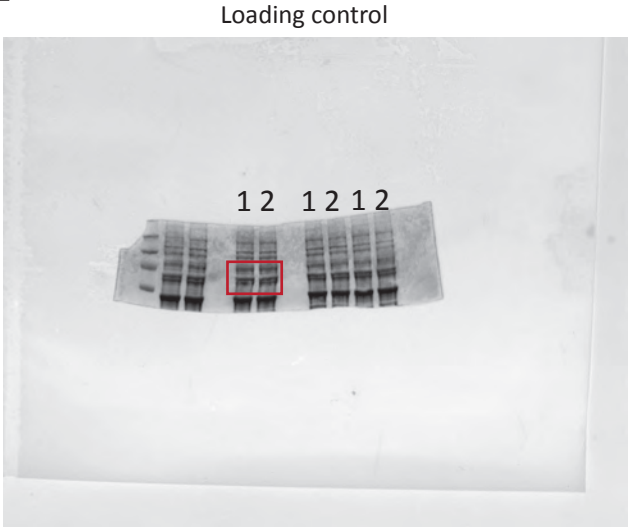

1-WT 2 -Mir10E

Supplement: Figure 5—figure supplement 1—source data 1. [file elife-90293-fig5-figsupp1-data1.zip › Figure 5,figure supplement 1/Figure 5, figure supplement 1-source data 5, uncropped and labelled gels.pdf]

Figure 5-figure supplement 1G-Mir1 levels in WT and Mir1OE

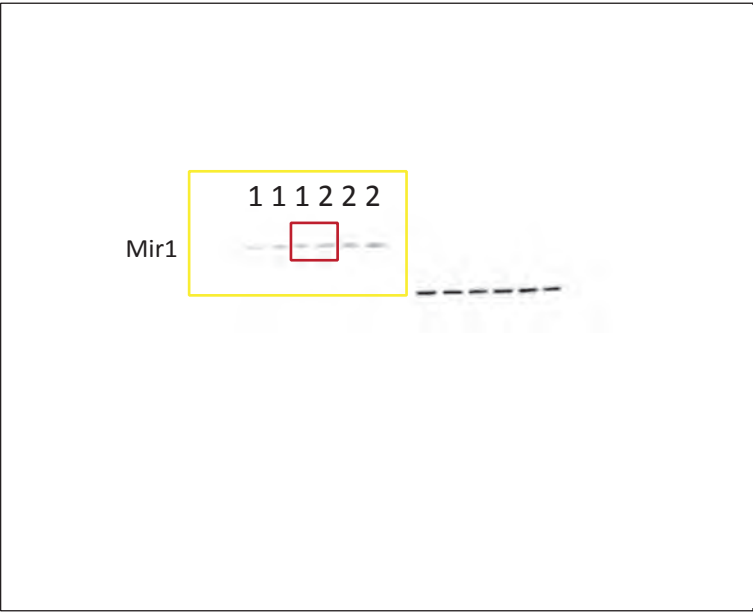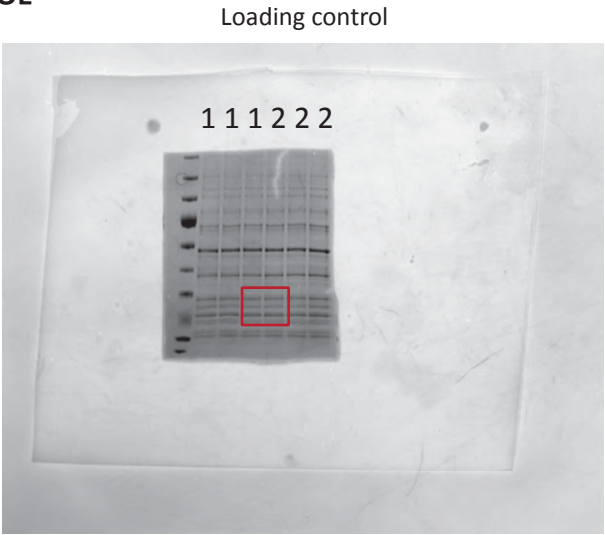

1-WT, 2- Mir1 OE

Supplement: Figure 5—figure supplement 1—source data 1. [file elife-90293-fig5-figsupp1-data1.zip › Figure 5,figure supplement 1/Figure 5, figure supplement 1-source data 2, uncropped and labelled gels.pdf]

Figure 5-figure supplement 1I-Mpc3 levels in WT, *mir1Δ*

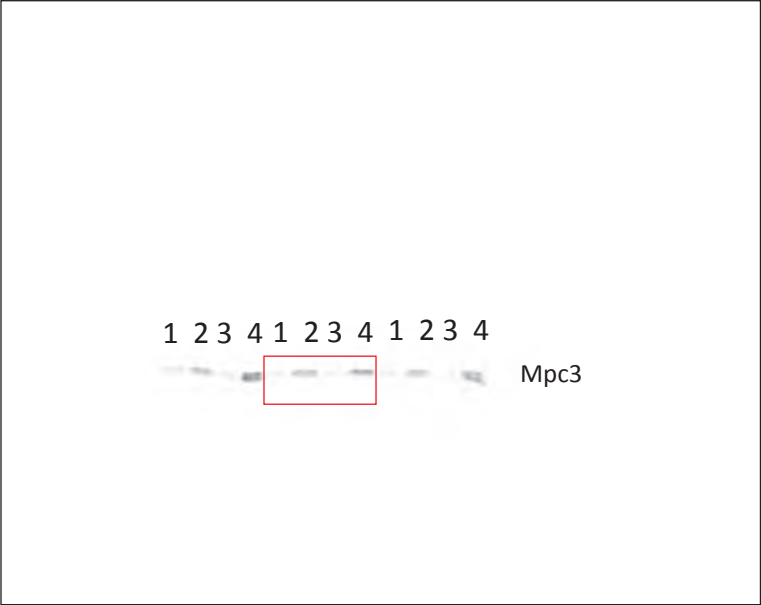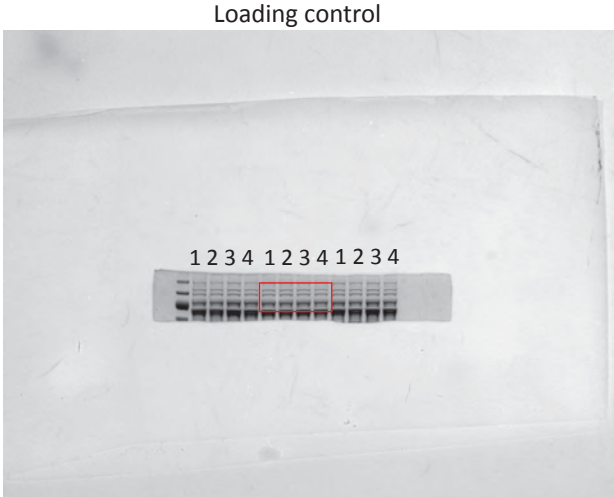

1 -WT 2% glc, 2-WT 0.1 % glc, 3- *mir1Δ* 2%glc,  
4- *mir1Δ* 0.1%glc

Supplement: Figure 5—figure supplement 1—source data 1. [file elife-90293-fig5-figsupp1-data1.zip › Figure 5,figure supplement 1/Figure 5, figure supplement 1-source data 4, uncropped and labelled gels.pdf]

Figure 5-figure supplement 1H-Mpc3 levels in WT, *ubp3Δ* and *ubp3Δ*-Pi

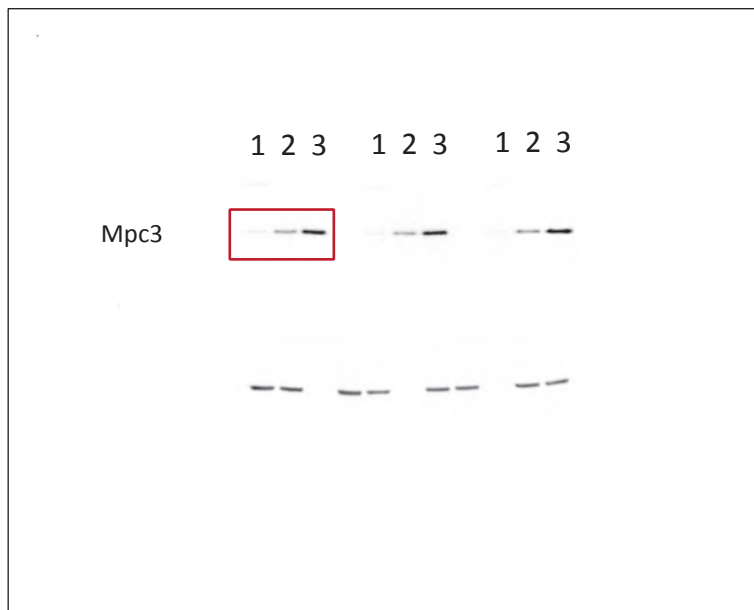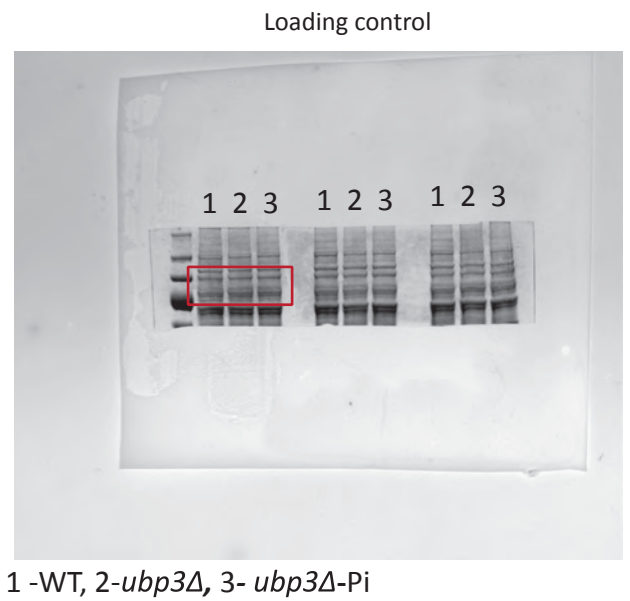

Supplement: Figure 5—figure supplement 1—source data 1. [file elife-90293-fig5-figsupp1-data1.zip › Figure 5,figure supplement 1/Figure 5, figure supplement 1-source data 3, uncropped and labelled gels.pdf]

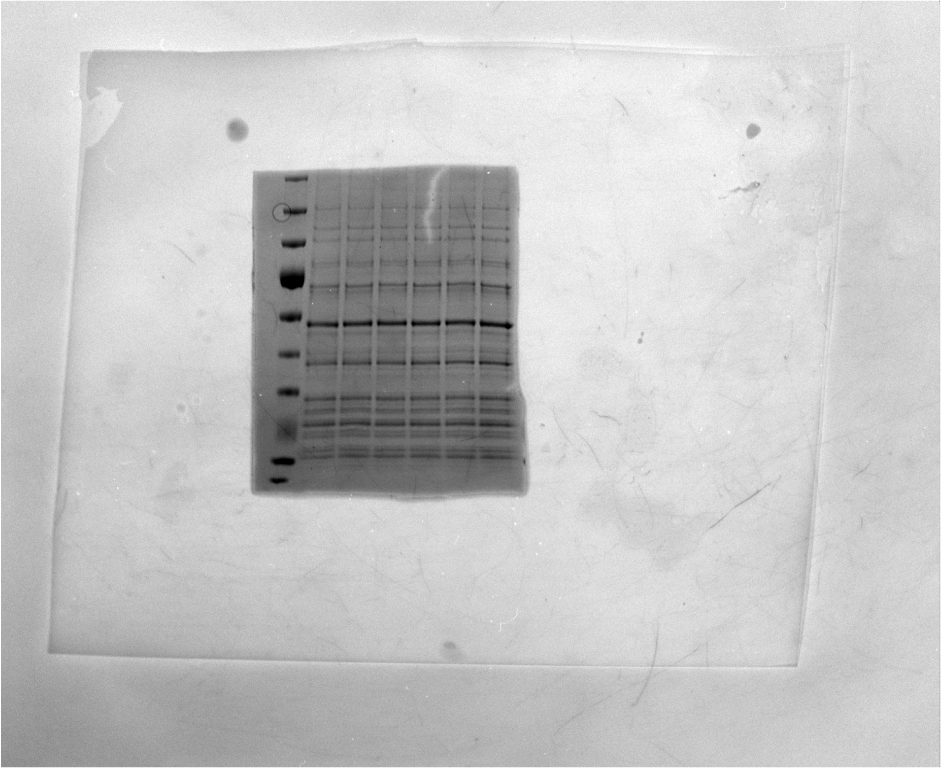

Supplement: Figure 5—figure supplement 1—source data 2. [file elife-90293-fig5-figsupp1-data2.zip › Figure 5, figure supplement 1 raw unedited/Figure 5, Supplement 1G loading.tif]

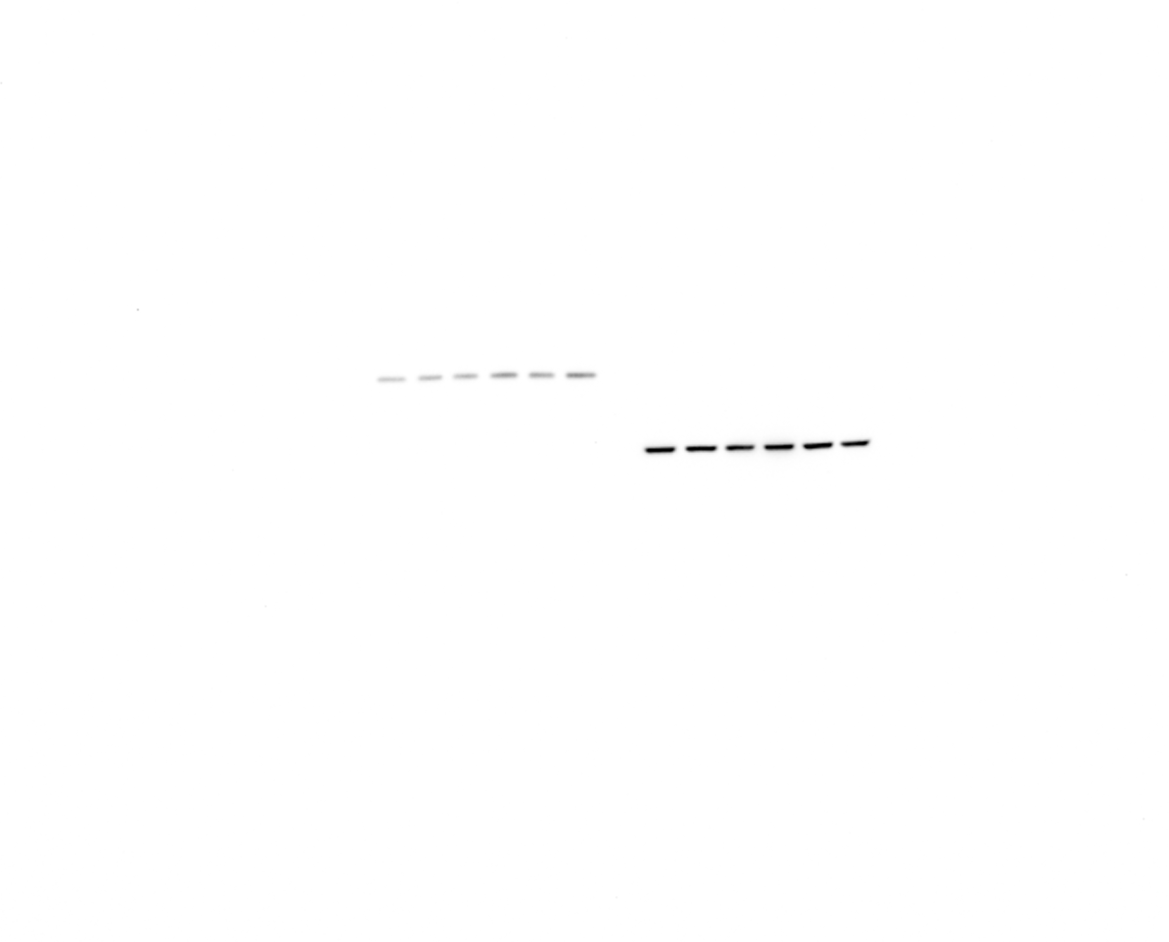

Supplement: Figure 5—figure supplement 1—source data 2. [file elife-90293-fig5-figsupp1-data2.zip › Figure 5, figure supplement 1 raw unedited/Figure 5, Supplement 1G.tif]

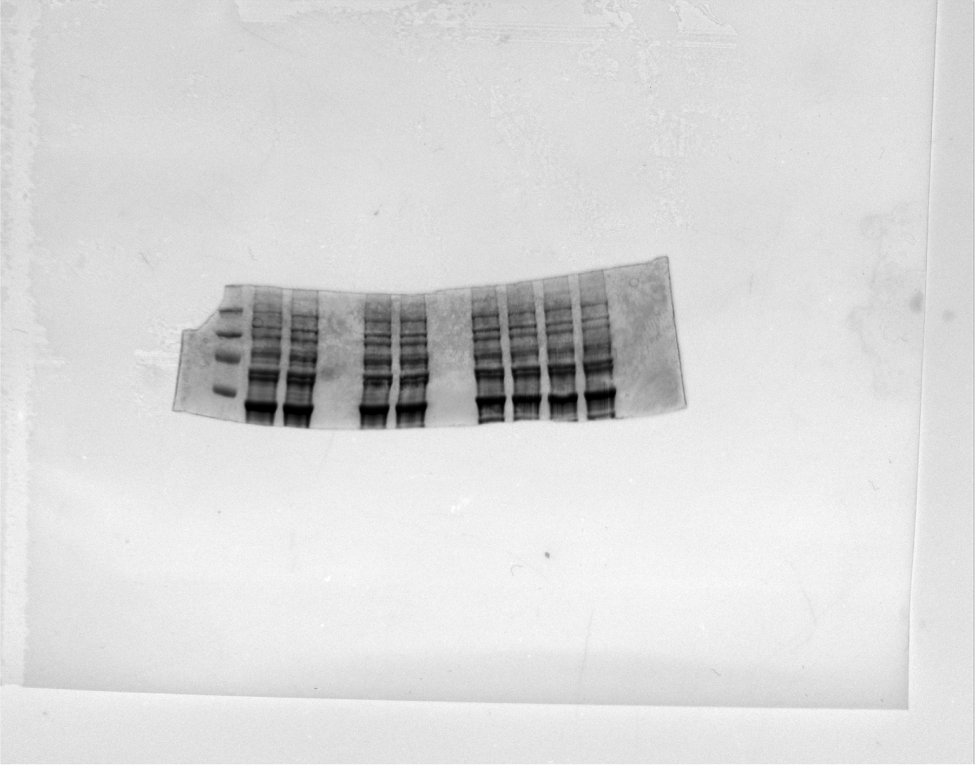

Supplement: Figure 5—figure supplement 1—source data 2. [file elife-90293-fig5-figsupp1-data2.zip › Figure 5, figure supplement 1 raw unedited/Figure 5, Supplement 1K loading.tif]

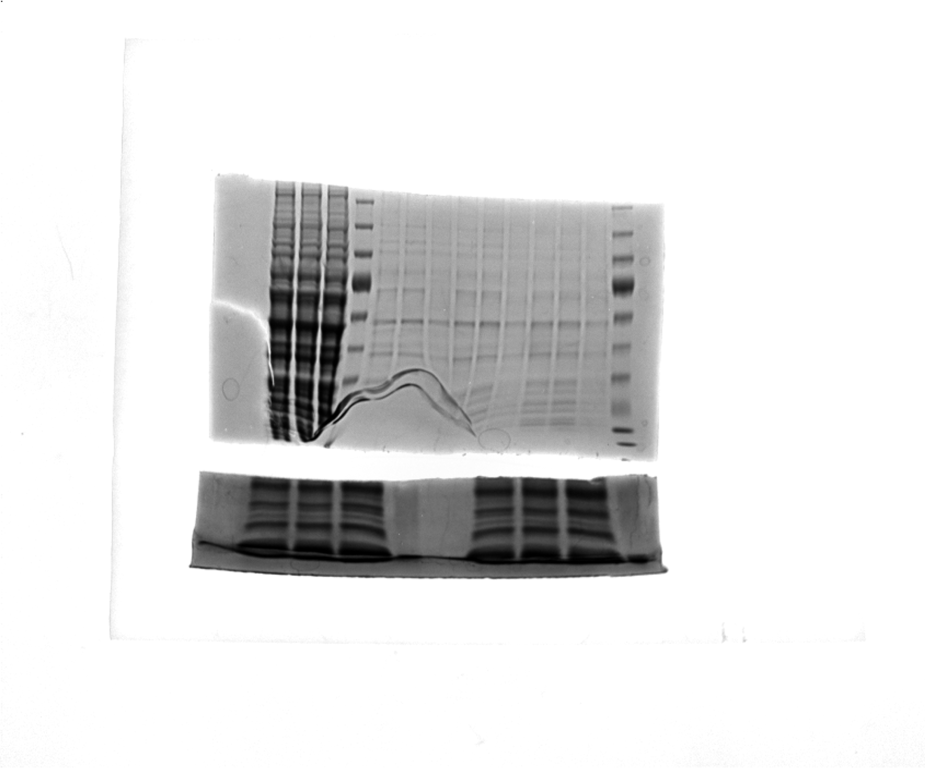

Supplement: Figure 5—figure supplement 1—source data 2. [file elife-90293-fig5-figsupp1-data2.zip › Figure 5, figure supplement 1 raw unedited/Figure 5, Supplement 1B loading.tif]

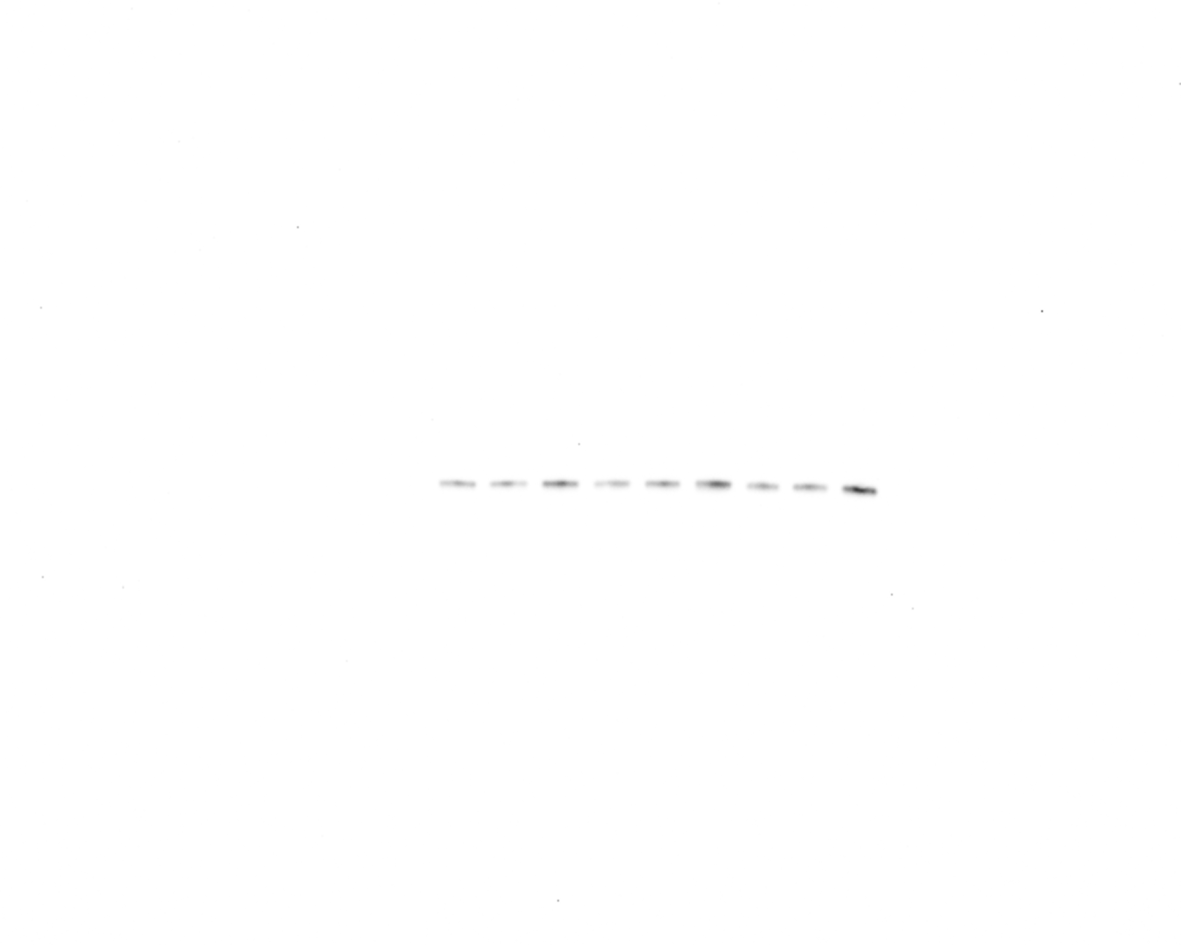

Supplement: Figure 5—figure supplement 1—source data 2. [file elife-90293-fig5-figsupp1-data2.zip › Figure 5, figure supplement 1 raw unedited/Figure 5, Supplement 1B.tif]

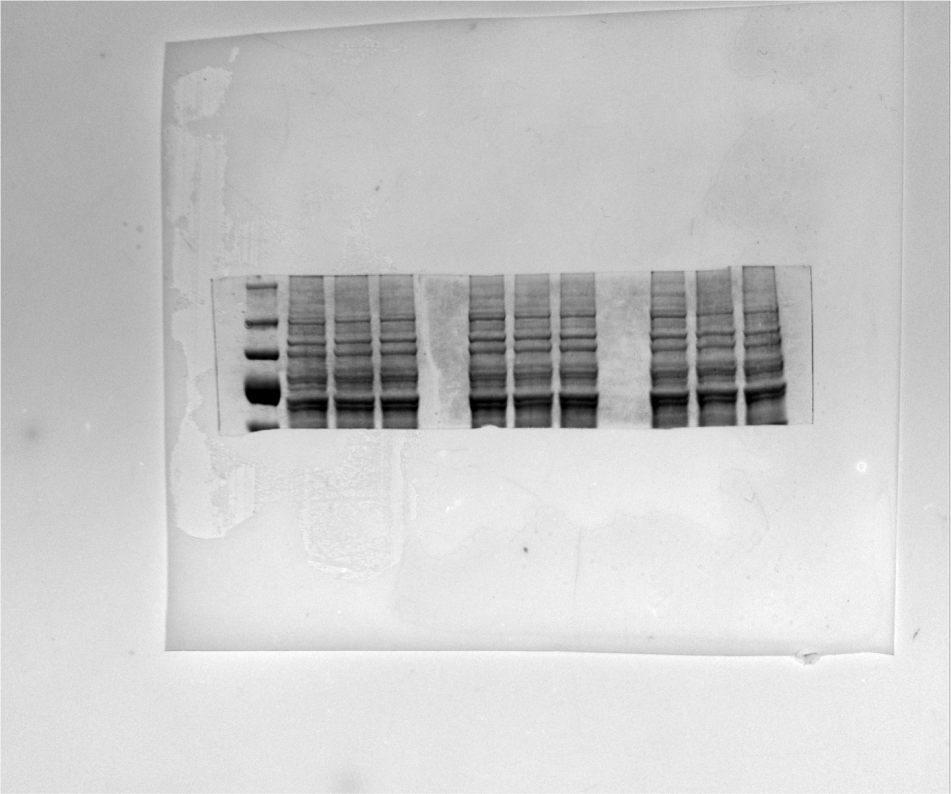

Supplement: Figure 5—figure supplement 1—source data 2. [file elife-90293-fig5-figsupp1-data2.zip › Figure 5, figure supplement 1 raw unedited/Figure 5, Supplement 1H loading.tif]

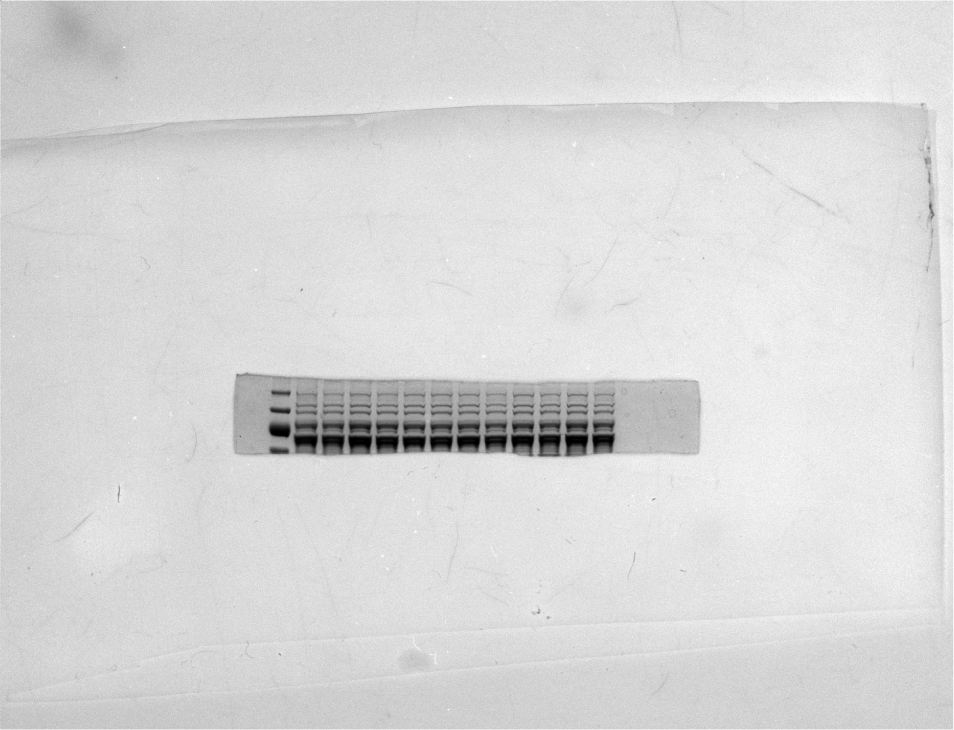

Supplement: Figure 5—figure supplement 1—source data 2. [file elife-90293-fig5-figsupp1-data2.zip › Figure 5, figure supplement 1 raw unedited/Figure 5, Supplement 1I loading.tif]

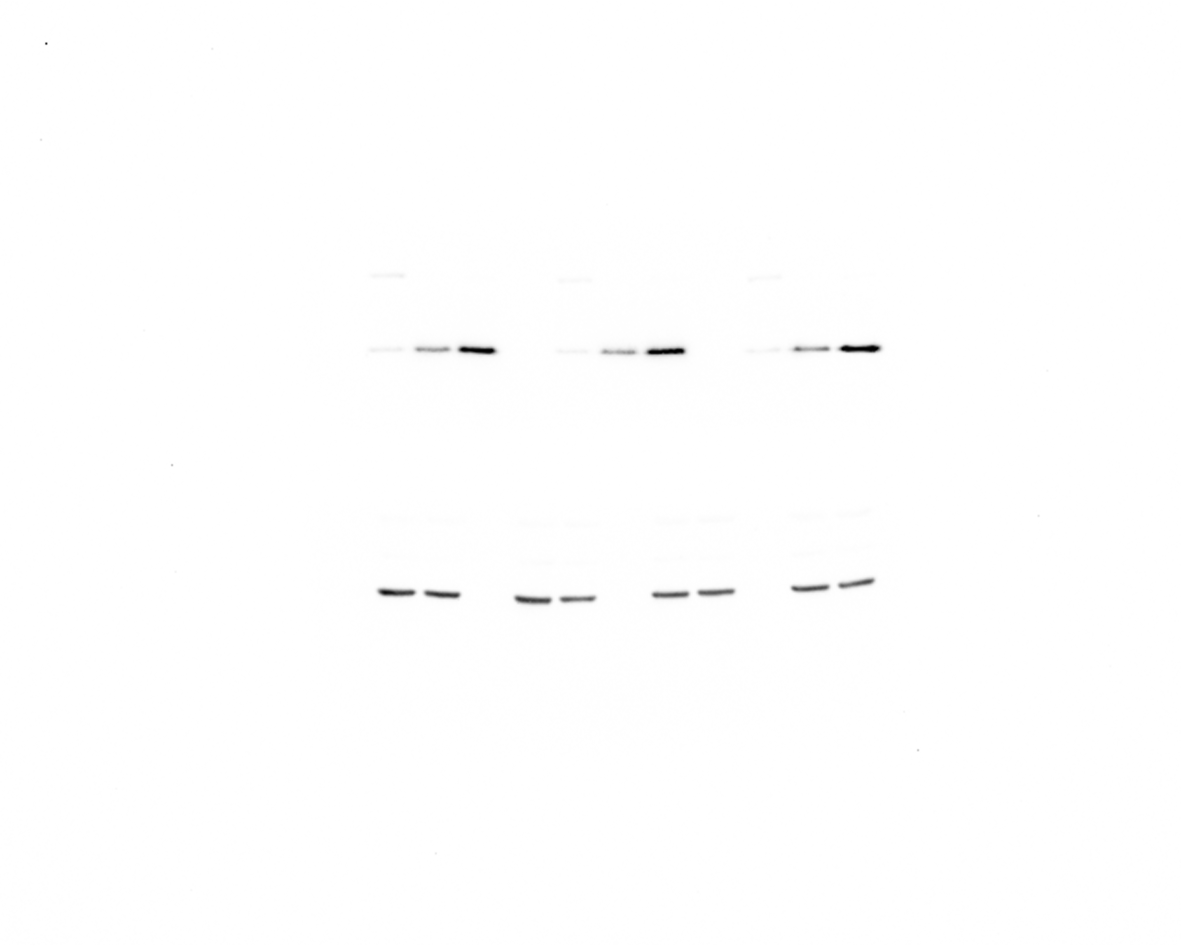

Supplement: Figure 5—figure supplement 1—source data 2. [file elife-90293-fig5-figsupp1-data2.zip › Figure 5, figure supplement 1 raw unedited/Figure 5, Supplement 1H.tif]

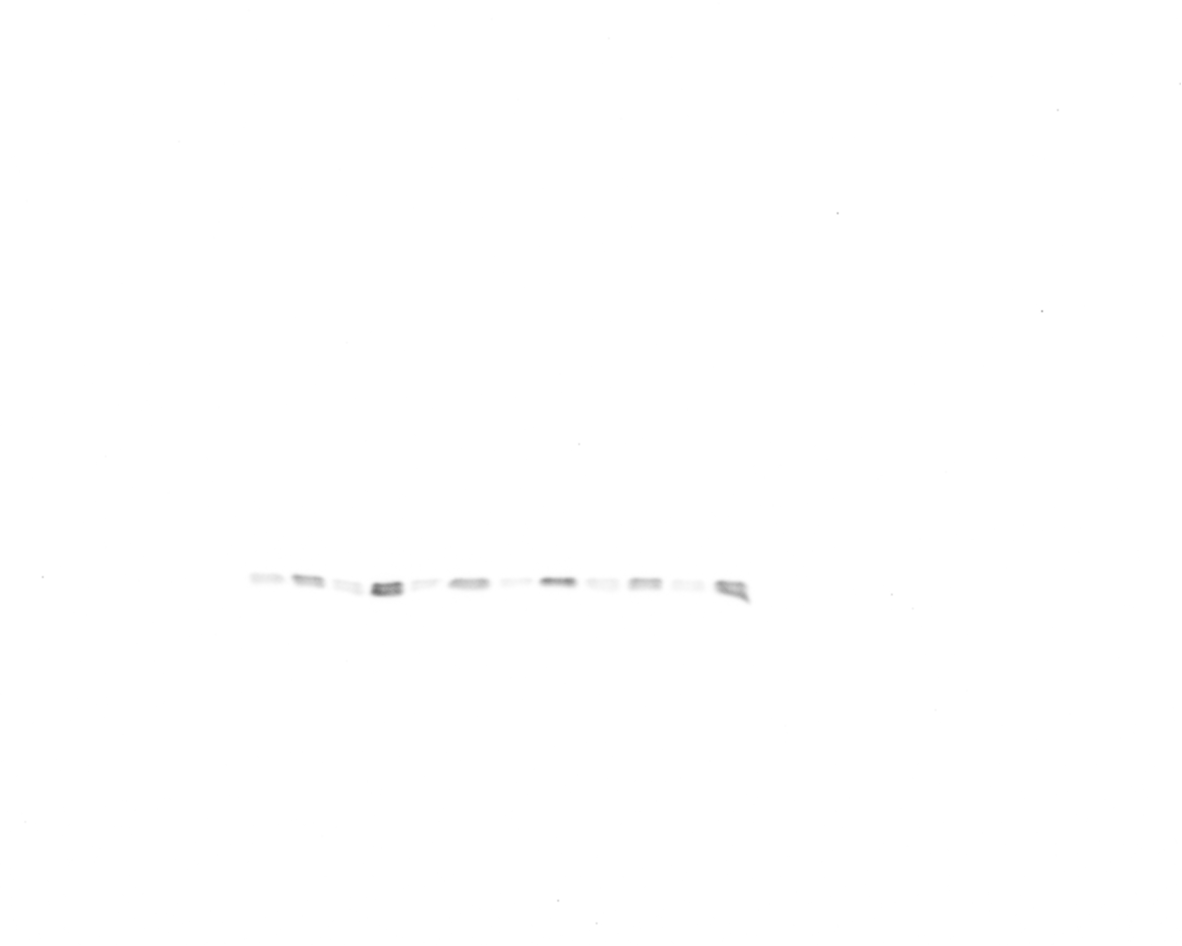

Supplement: Figure 5—figure supplement 1—source data 2. [file elife-90293-fig5-figsupp1-data2.zip › Figure 5, figure supplement 1 raw unedited/Figure 5, Supplement 1I.tif]

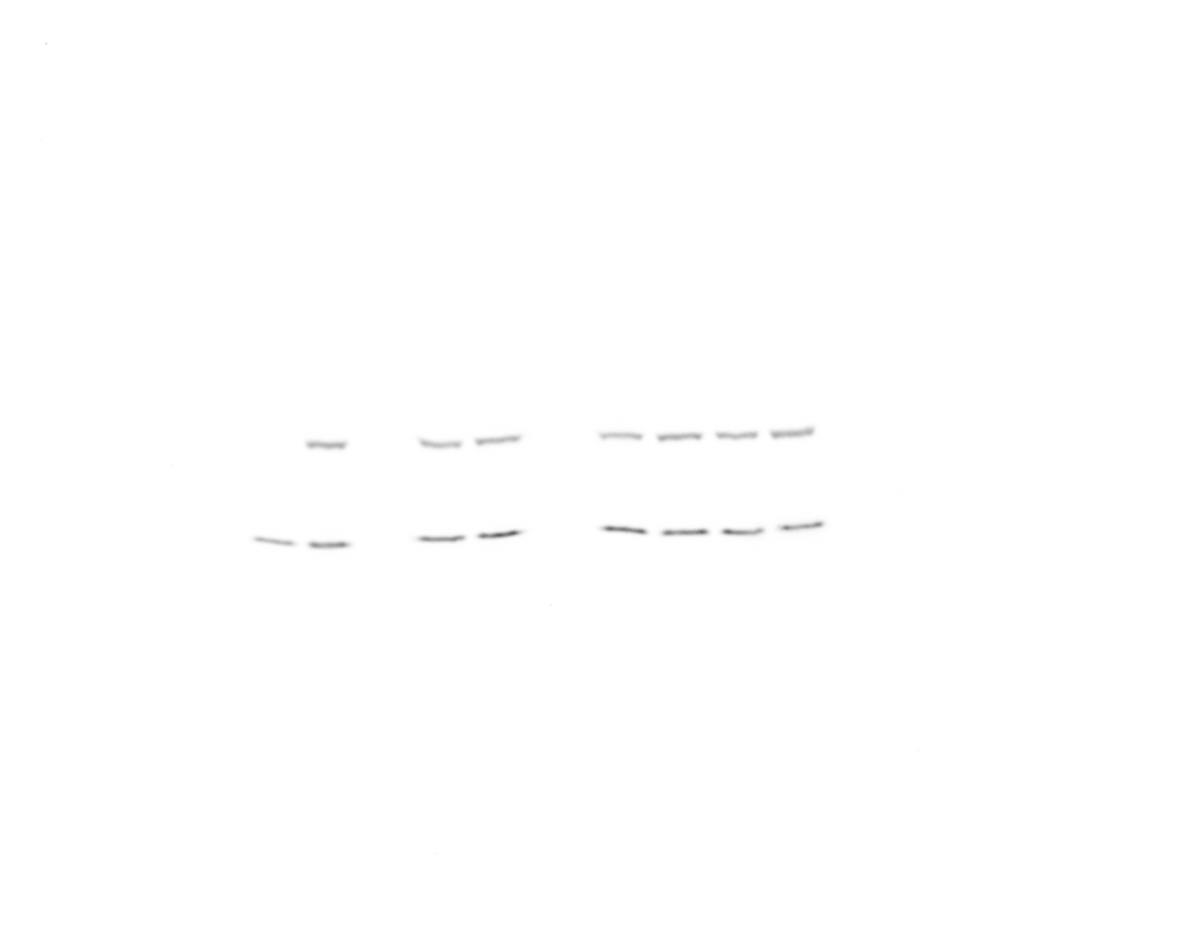

Supplement: Figure 5—figure supplement 1—source data 2. [file elife-90293-fig5-figsupp1-data2.zip › Figure 5, figure supplement 1 raw unedited/Figure 5, Supplement 1K.tif]

Figure 6B- Cox2 levels in WT vs ubp3Δ

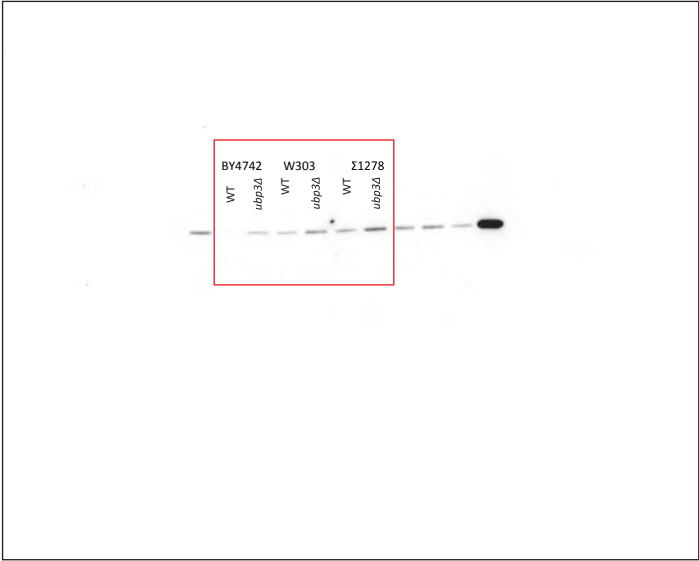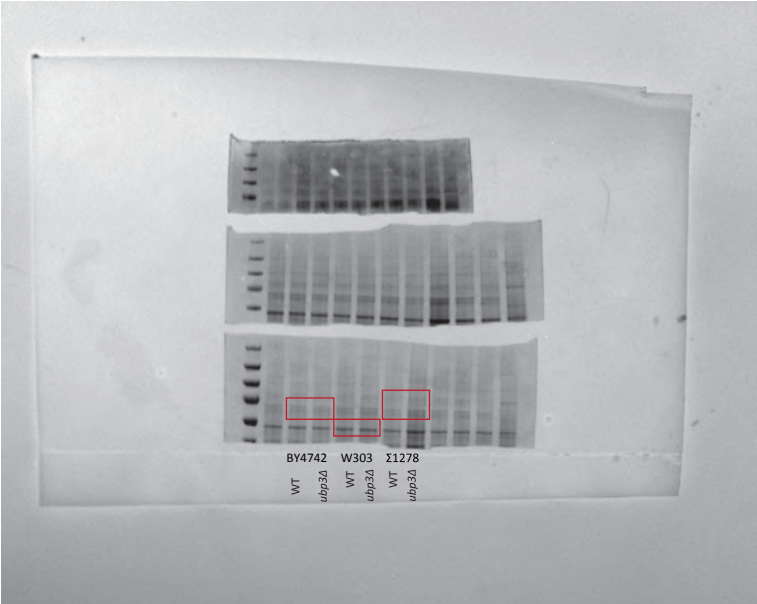

Supplement: Figure 6—source data 1. [file elife-90293-fig6-data1.zip › Figure 6/Figure 6-source data 1, uncropped and labelled gels.pdf]

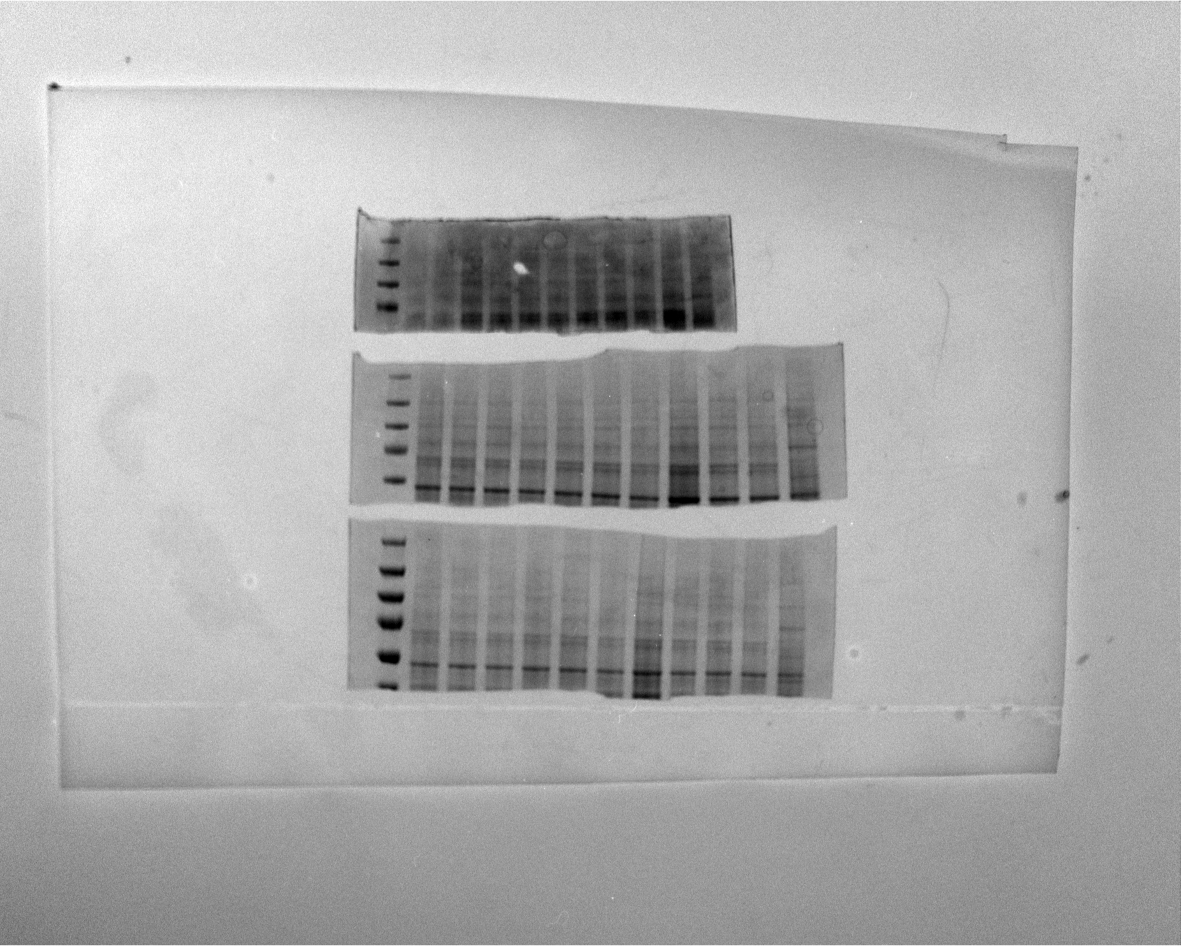

Supplement: Figure 6—source data 2. [file elife-90293-fig6-data2.zip › Figure 6 raw unedited/Figure 6B loading.tif]

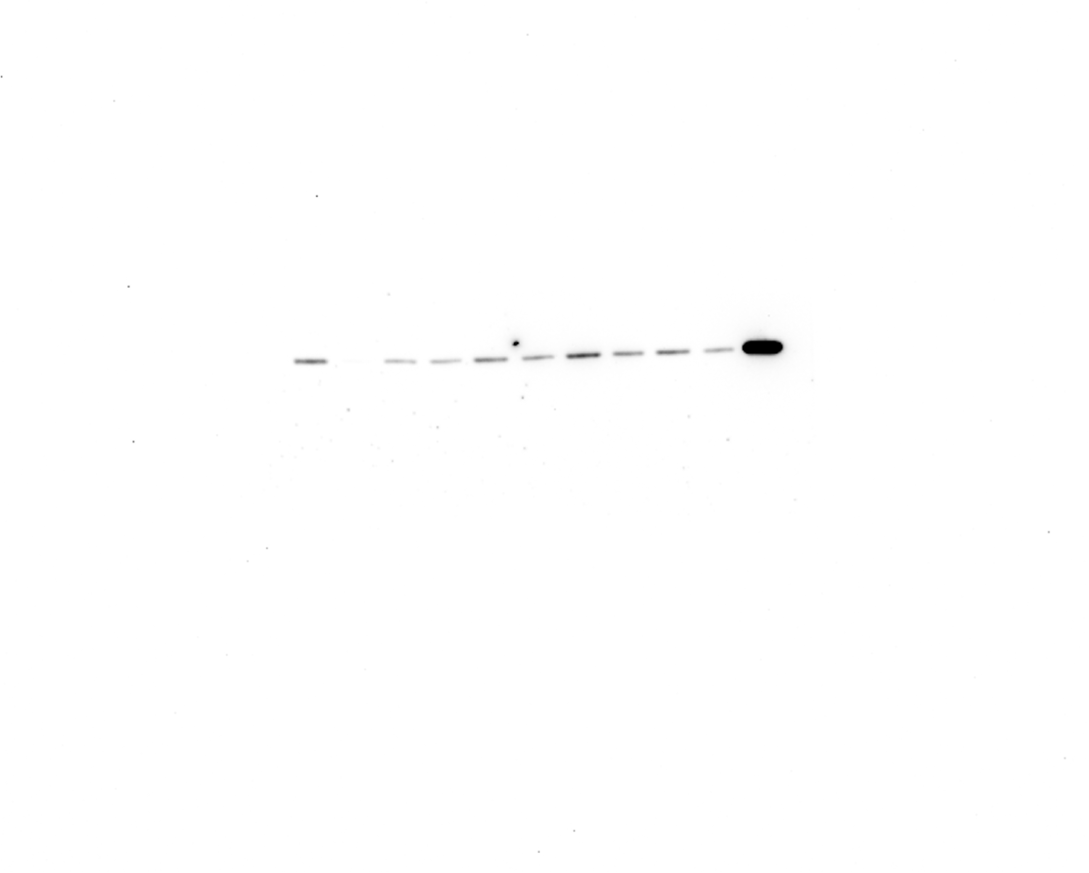

Supplement: Figure 6—source data 2. [file elife-90293-fig6-data2.zip › Figure 6 raw unedited/Figure 6B.tif]
