# Supplementary material for: The deubiquitinase Ubp3/Usp10 constrains glucose-mediated mitochondrial repression via phosphate budgeting
Source: eLife. 2024 Sep 26;12:RP90293. doi: 10.7554/eLife.90293 (PMC11426969; doi:10.7554/eLife.90293)

Figure 2-figure supplement 1B-Pfk1, Tdh2 and Tdh3 levels in WT and ubp3 cells with/without MG132

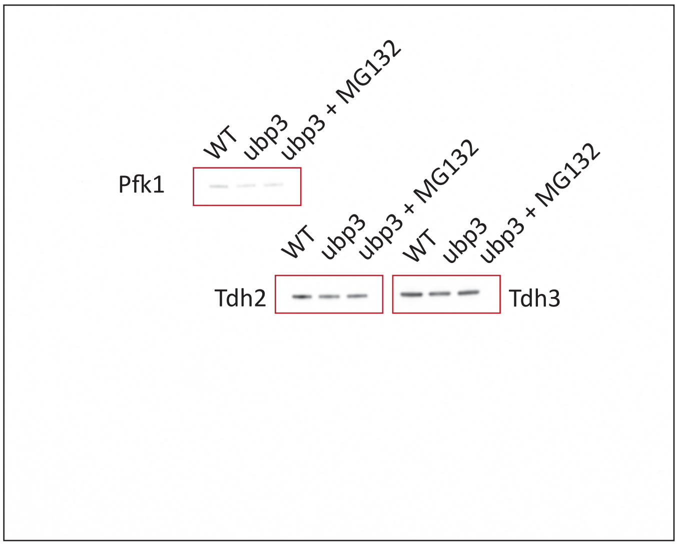

High exposure

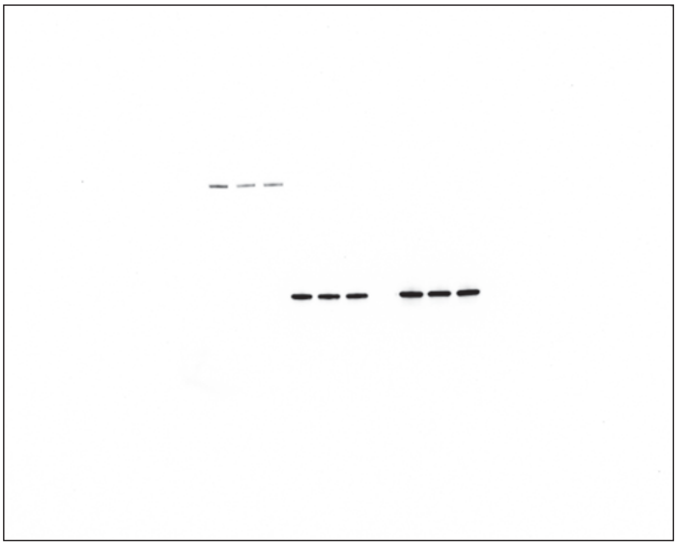

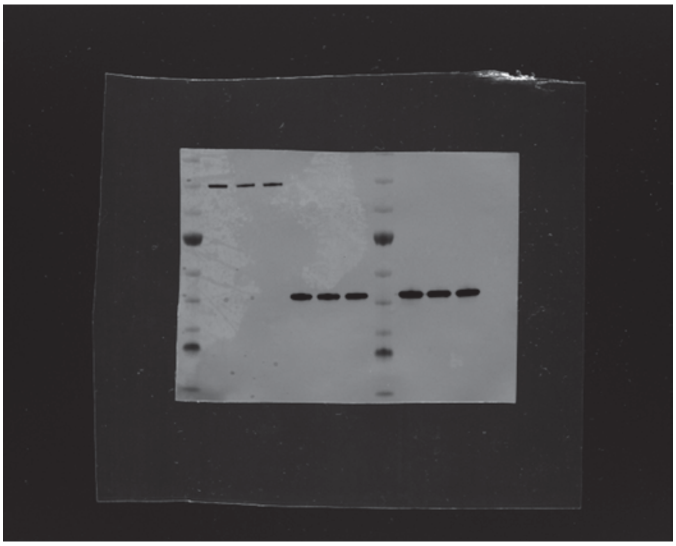

Loading

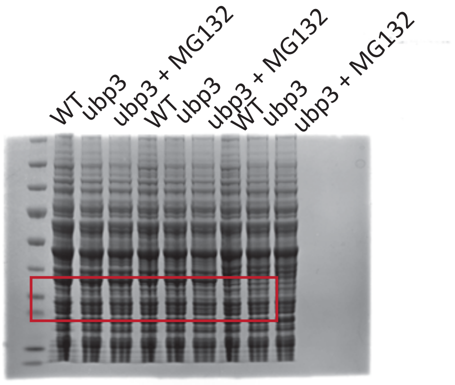

Supplement: Figure 2—figure supplement 1—source data 1. [file elife-90293-fig3-figsupp3-data3.zip › Figure 2, figure supplement 1/Figure 2, figure supplement 1-source data 3, uncropped and labelled gels.pdf]
