# Supplementary material for: The deubiquitinase Ubp3/Usp10 constrains glucose-mediated mitochondrial repression via phosphate budgeting
Source: eLife. 2024 Sep 26;12:RP90293. doi: 10.7554/eLife.90293 (PMC11426969; doi:10.7554/eLife.90293)
Supplement: Figure 4—source data 1. [file elife-90293-fig4-data1.zip › Figure 4/Figure 4-source data 1, uncropped and labelled gels.pdf]

Figure 4C and 4E-Cox 2 levels in WT,*ubp3Δ* and *tdh2Δtdh3Δ* cells

Loading control

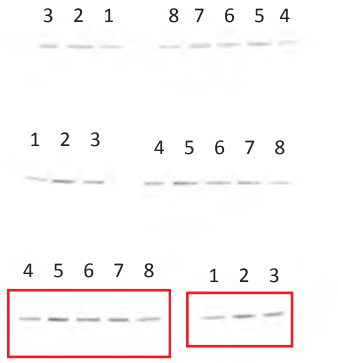

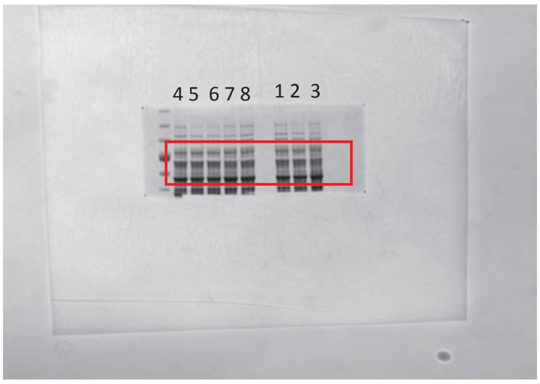

1-WT, 2- *ubp3Δ, 3 - tdh2Δtdh3Δ,* 4- WT, 5-*ubp3Δ, 6- ubp3Δ*-Pi,
7-*tdh2Δtdh3Δ,* 8-*tdh2Δtdh3Δ-Pi*
