# Supplementary material for: The deubiquitinase Ubp3/Usp10 constrains glucose-mediated mitochondrial repression via phosphate budgeting
Source: eLife. 2024 Sep 26;12:RP90293. doi: 10.7554/eLife.90293 (PMC11426969; doi:10.7554/eLife.90293)

Figure 4-Figure supplement 1A Eno1 and Cox2 in cytosolic fraction

1-WT, 2- WT+Pi, 3- *ubp3Δ*

Lysate

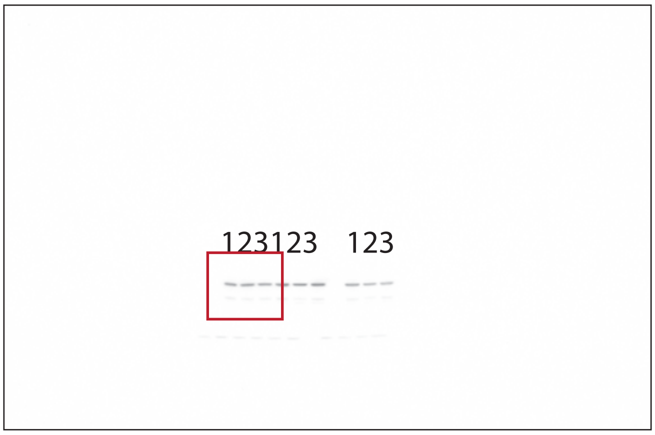

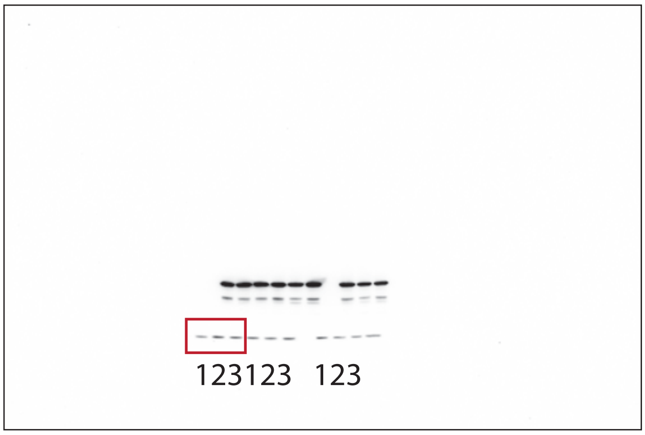

Cytosolic fraction

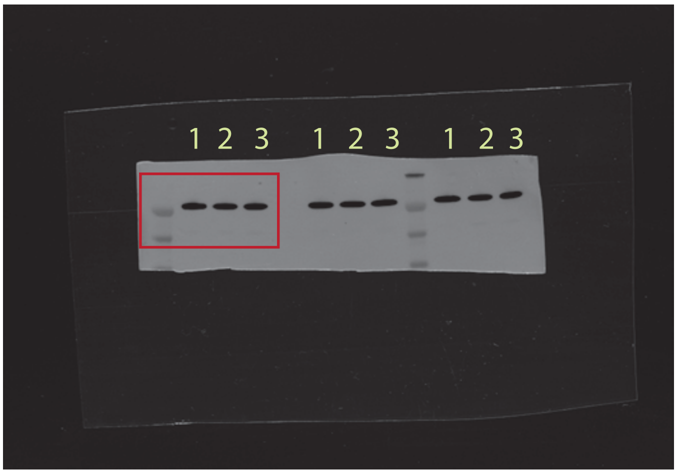

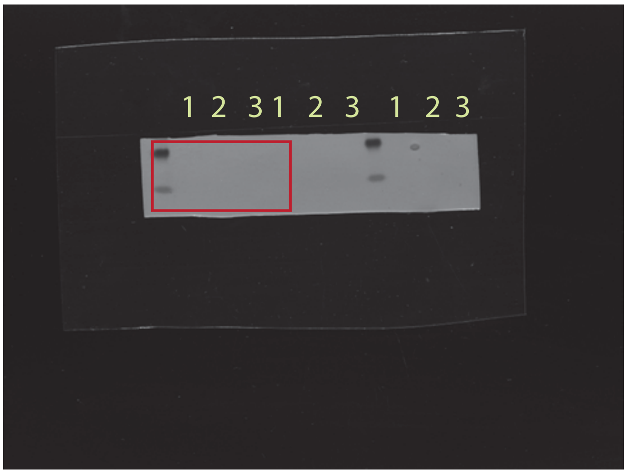

Supplement: Figure 4—figure supplement 1—source data 1. [file elife-90293-fig5-figsupp5-data5.zip › Figure 4,figure supplement 1/Figure 4, figure supplement 1-source data 1, uncropped and labelled gels.pdf]
